# The burden of chronic diseases and patients' preference for healthcare services among adult patients suffering from chronic diseases in Bangladesh

**DOI:** 10.1111/hex.13634

**Published:** 2022-10-20

**Authors:** Rashidul A. Mahumud, Marufa Sultana, Satyajit Kundu, Md. A. Rahman, Sabuj K. Mistry, Joseph K. Kamara, Mostafa Kamal, Mohammad A. Ali, Md. G. Hossain, Cristy Brooks, Asaduzzaman Khan, Khorshed Alam, Andre M. N. Renzaho

**Affiliations:** ^1^ Health Research Group, Department of Statistics University of Rajshahi Rajshahi Bangladesh; ^2^ NHMRC Clinical Trials Centre, Faculty of Medicine and Health The University of Sydney Camperdown New South Wales Australia; ^3^ School of Business, Faculty of Business, Education, Law and Arts, and Centre for Health Research University of Southern Queensland Toowoomba Queensland Australia; ^4^ Deakin Health Economics, School of Health and Social Development Deakin University Geelong Victoria Australia; ^5^ Department of Biochemistry and Food Analysis Patuakhali Science and Technology University Patuakhali Bangladesh; ^6^ Development Studies Discipline Khulna University Khulna Bangladesh; ^7^ Centre for Primary Health Care and Equity University of New South Wales Sydney New South Wales Australia; ^8^ ARCED Foundation Mirpur Dhaka Bangladesh; ^9^ Department of Public Health Daffodil International University Dhaka Bangladesh; ^10^ Regional Director, Humanitarian & Emergency Affairs, World Vision International East Africa Regional Office Karen Nairobi Kenya; ^11^ BL Deakin Business School, Faculty of Business and Law Deakin University Geelong Victoria Australia; ^12^ Quality Use of Medicines and Pharmacy Research Centre (QUMPRC), Clinical and Health Sciences University of South Australia Adelaide South Australia Australia; ^13^ Translational Health Research Institute Western Sydney University Sydney New South Wales Australia; ^14^ School of Medicine Western Sydney University Sydney New South Wales Australia; ^15^ School of Health and Rehabilitation Science The University of Queensland Brisbane Queensland Australia; ^16^ Maternal, Child and Adolescent Health Program Burnet Institute Melbourne Victoria Australia

**Keywords:** Bangladesh, chronic diseases, healthcare services, patient preferences

## Abstract

**Background:**

Low‐ and middle‐income countries (LMICs) have a disproportionately high burden of chronic diseases, with inequalities in health care access and quality services. This study aimed to assess patients' preferences for healthcare services for chronic disease management among adult patients in Bangladesh.

**Methods:**

The present analysis was conducted among 10,385 patients suffering from chronic diseases, drawn from the latest Household Income and Expenditure Survey 2016–2017. We used the multinomial logistic regression to investigate the association of chronic comorbid conditions and healthcare service‐related factors with patients' preferences for healthcare services.

**Results:**

The top four dimensions of patient preference for healthcare services in order of magnitude were quality of treatment (30.3%), short distance to health facility (27.6%), affordability of health care (21.7%) and availability of doctors (11.0%). Patients with heart disease had a 29% significantly lower preference for healthcare affordability than the quality of healthcare services (relative risk ratio [RRR] = 0.71; 0.56–0.90). Patients who received healthcare services from pharmacies or dispensaries were more likely to prefer a short distance to a health facility (RRR = 6.99; 4.80–9.86) or affordability of healthcare services (RRR = 3.13; 2.25–4.36). Patients with comorbid conditions were more likely to prefer healthcare affordability (RRR = 1.39; 1.15–1.68). In addition, patients who received health care from a public facility had 2.93 times higher preference for the availability of medical doctors (RRR = 2.93; 1.70–5.04) than the quality of treatment in the health facility, when compared with private service providers.

**Conclusions:**

Patient preferences for healthcare services in chronic disease management were significantly associated with the type of disease and its magnitude and characteristics of healthcare providers. Therefore, to enhance service provision and equitable distribution and uptake of health services, policymakers and public health practitioners should consider patient preferences in designing national strategic frameworks for chronic disease management.

**Patient or Public Contribution:**

Our research team includes four researchers (co‐authors) with chronic diseases who have experience of living or working with people suffering from chronic conditions or diseases.

## 1 BACKGROUND

Chronic diseases have become a global challenge, imposing an enormous economic and health burden on society.^1^ Chronic diseases are defined as health conditions lasting 12 months or more, which require ongoing medical intervention and may result in the limitation of activities of daily living.^1^ The epidemiological burden of chronic diseases^2^ and exposure to their risk factors are increasing worldwide,^3^ particularly in low‐ and middle‐income countries (LMICs), such as Bangladesh.^4^, ^5^, ^6^ Chronic diseases account for around 41 million deaths each year, representing about 71% of all deaths globally.^7^ The most common chronic diseases include cardiovascular diseases (e.g., coronary heart diseases, stroke and peripheral vascular diseases), diabetes, cancers, chronic obstructive pulmonary disease, mental illness and arthritis^7^; approximately 77% of all yearly deaths are related to chronic diseases occur in LMICs, of which 85% occur in the most productive age groups (30–69 years).^7^ It has been estimated that chronic diseases will account for an accumulated global economic loss of 47 trillion US dollars by 2030, approximately 75% of the global gross domestic product.^8^ In Bangladesh, an LMIC with a substantial social and economic burden, about 886,000 deaths (i.e., 59% of total deaths) occur due to chronic diseases each year.^3^, ^9^ The burden due to chronic diseases has increased in Bangladesh from 43.4% in 2000 to 66.9% in 2015.^3^ It is anticipated that chronic diseases will exceed the combined burden of communicable, maternal, perinatal and nutrition‐related diseases by 2030 globally, including in Bangladesh.^10^, ^11^

Despite the high burden of chronic diseases, Bangladesh, like many LMIC countries, does not have a national integrated chronic diseases management policy, strategy or action plan.^9^, ^11^ For instance, the prevalence of undiagnosed chronic diseases is high, and the proportion of unmanaged chronic diseases is even higher in many LMICs,^10^ including Bangladesh.^12^ This highlights the frequent inadequacies in the diagnosis, prevention and management of chronic diseases among the healthcare systems of LMICs.^9^, ^10^ Efforts in chronic disease management in Bangladesh continue to be inadequate. Little attention has been given to addressing the contributing behaviours associated with chronic diseases, including unhealthy dietary patterns, lack of physical activity and exposure to factors potentially detrimental to health such as alcohol, drug and tobacco use.^6^ However, it is possible to counteract the rising prevalence of the chronic disease by implementing effective prevention strategies, population‐based screening, reduction of risk factors, early detection and appropriate treatments.^10^, ^11^ If such actions are not taken, the burden of chronic diseases, which is referred to as an emerging prevalence of chronic diseases globally imposing an enormous economic and health burden will likely continue to rise,^10^, ^11^ which is alarming especially among the vulnerable and marginalized populations of Bangladesh with limited affordability for health services.^11^ In addition, the emerging prevalence of chronic disease may also lead to a health system burden in terms of increasing healthcare utilization, treatment costs and chronic disease management.

It is well established that chronic diseases are increasingly associated with the over‐utilization of healthcare services and a higher financial burden.^13^ For example, a previous study documented that a higher number of chronic diseases was linked to an increased number of outpatient visits.^14^ Therefore, adequate preventative services must be in place to reduce the social‐economic burden of chronic disease, thereby ensuring optimal use of health resources. However, the major challenges to ensure effective prevention and management of chronic diseases include social status, power gradients, racial/ethnic differences, poor accessibility and affordability of healthcare services.^2^, ^6^, ^15^, ^16^, ^17^ Previous research has identified several factors associated with healthcare utilization, such as demographic and socioeconomic characteristics, type of healthcare providers and presence of chronic illnesses.^18^ In addition, patients' preference for healthcare services depends on several factors, including personal preference, disease severity, economic capacity, the reputation of healthcare providers^18^ as well as affordable costs associated with treatment.^19^ Furthermore, short travel time to healthcare facilities, effective interactions with healthcare providers,^19^, ^20^ respectful service provider attitudes^21^ and short waiting time^22^ were positively associated with the patient preference for healthcare services. A recent discrete choice experiment study found that the availability of medicine and transport to the health facility were significant attributes of patient preference for healthcare services.^23^ Notably, optimum healthcare utilization among chronically ill patients has a significant role in preventing and managing chronic diseases.

In Bangladesh, a family spends an average of 11% of their total household budget on health care and half of the population spends 7% of their monthly per capita consumption expenditure on illness.^24^ Understanding patients' preferences could help medical professionals and healthcare providers restructure the healthcare delivery model and ensure the quality of services. In addition to clinical guidelines, patients' preferences may also provide guidelines for the selection of treatment options. Patient preferences also help to inform clinical decisions where science has not yet been able to provide effective solutions to healthcare problems.^25^ Therefore, information on patients' preferences for healthcare utilization in terms of healthcare expenditure is critical; without this knowledge, an effective national healthcare policy cannot be formulated.^26^ Moreover, there is a current lack of evidence on the patients' preferences for healthcare services in chronic disease management in Bangladesh. Since no such study exists, the present study aimed to investigate patients' preference for healthcare services among adult patients in Bangladesh. Understanding patients' preferences are important in designing interventions aimed at reducing the burden of chronic diseases, which undermine economic growth and development and are increasingly becoming the leading cause of death.

## 2 METHODS

### 2.1 Study design and data sources

This study followed an observational, cross‐sectional design. The data was extracted from the most recent nationally representative Household Income and Expenditure Survey (HIES), conducted during 2016–2017 by the Bangladesh Bureau of Statistics (BBS) (Supporting Information: Document 1). The HIES is a cross‐sectional survey conducted every 5 years since 1973 in Bangladesh with the objective of providing national estimates on income, expenditure and consumption, poverty, standard of living, health status and education.^27^

In the HIES survey, a semistructured questionnaire (Supporting Information: Appendix Table A1: HIES 2016–2017 Questionnaire) was used to collect information from the survey participants (adults aged 18 years or above) under nine modules: (1) household information, (2) education, (3) health‐illnesses and injuries, (4) economic activities and wage employment, (5) nonagricultural enterprises, (6) housing, (7) agriculture, (8) other assets and income and (9) consumption (Supporting Information: Appendix Table A1: HIES 2016–2017 Questionnaire). However, our analysis is based on the chronic disease‐related questions included in Module‐3: Health (Illnesses and Injuries) of the HIES (Supporting Information: Appendix Table A2). Therefore, we only used the indicators pertaining to chronic disease and health service utilization along with the sociodemographic characterizes of the participants (Supporting Information: Appendix Table A2). All health‐related information was self‐reported in the HIES. Respondents were asked to prioritize the chronic diseases they were suffering from in order of their importance. We selected the primary disease (i.e., principal diagnosis) based on the patients' experience of diseases in order of their importance. For instance, if one patient was diagnosed with three chronic conditions, they reported three diseases in order of importance (i.e., first, second and third importance). Therefore, a patient's first importance of disease was considered as the primary disease in the present study.

### 2.2 HIES survey sampling and sample size calculation

The sample frame used in the selection of Primary Sample Units (PSUs) for the HIES 2016–2017 was based on the Census of Population and Housing 2011.^27^ In this survey, eight administrative divisions (Barisal, Chittagong, Dhaka, Khulna, Mymensingh, Rajshahi, Rangpur and Sylhet) were included and stratified by three basic localities, being rural, urban and metropolitan areas (city corporations). Thus, these should be 8 divisions × 3 localities totalling 24 strata (8 × 3 = 24). However, the sampling frame (Census of Population and Housing 2011) did not contain three administrative divisions (i.e., Rangpur, Barisal and Sylhet). Additionally, the BBS included only four main city corporations (Dhaka, Chittagong, Khulna and Rajshahi) in the city corporation locality. PSUs were randomly selected from 20 strata (eight rural divisions, eight urban divisions and four metropolitan areas) for national representation. As the PSUs of HIES 2016–2017 were allocated at the district level, the sample design includes a total of 132 substrata: 64 rural, 64 urban and 4 metropolitan areas.^27^ Sample size was calculated using the prevalence rate of the main indicator (poverty rate) or the coefficient of variation of per capita consumption or household consumption, which are the core indicators of the HIES 2016–2017. Each one was treated as a target variable for determining the sample size (Supporting Information: Document 1). The required sample size was calculated for each district and explained elsewhere,^27^ using the following formula

(1) $$n={\left(\frac{Z_{\frac{\alpha }{2}}\times {\mathrm{CV}}_{\mathrm{SRS}\left(\bar{y}\right)}}{r\left(\bar{Y}\right)}\right)}^{2}\times \mathrm{DEFF},$$

where, *n* was the required sample for allocation to each district to achieve a certain level in the accuracy statistic ($r\left(\overset{®}{Y}\right)$ = 10% relative standard error desired for the mean total household expenditure estimated at the district level associated with the targeted variable $\left(\overset{®}{y}\right);{\mathrm{CV}}_{\mathrm{SRS}\left(\overset{®}{y}\right)}$ was the coefficient of variation of the targeted variable [i.e., total household expenditure estimated at the national level] estimated under the assumption of simple random sampling; DEFF was the design effect of the target variable [i.e., the average design effect of the target variable across all districts]; and $Z_{\frac{\alpha }{2}}$ [=1.96] was the critical value of a standard normal distribution with an *α*% (5%) significance level). Substituting all values in Equation (1), the required sample was 715 households for each district, nonetheless, 720 households were allocated to each district for practical consideration and to facilitate fieldwork and survey implementation management. A stratified, two‐stage cluster sampling technique was used in this survey. In the first stage, a total of 36 PSUs were drawn from each district by applying the probability proportional to size systematic sampling technique, using the number of households in each PSU as the measure of size. The 36 PSUs were randomly selected from rural, urban and city corporation substratum. The total number of PSUs included in the analysis was 2304 (64 districts × 36 PSU per district). In the second stage, 20 households were selected per PSU. Using this sampling technique, 46,076 households (2304 PUSs × 20 households per PSU) were included in the study analysis of HIES 2016–2017 data. Among the selected households, a total of 186,076 individuals were included.

## 3 DATA COLLECTION

Data collection was accomplished between early April 2016 and late March 2017 through face‐to‐face interviews with the participants. A total of 128 enumeration teams, each comprised of five members (one supervising officer, two interviewers and two female facilitators) who were thoroughly trained, completed the data collection. A multilayered quality control measure was employed to ensure the quality of the collected data. The supervising officer of each team was responsible to verify all the questionnaires completed by the field staff before they were sent to the headquarter. Moreover, Deputy Directors of the District Statistical Offices as well as senior officials from the headquarter, frequently visited the sampled areas to ensure data quality (Supporting Information: Document 1).

The survey team formulated an operational definition of each variable used in the questionnaire. For example, while collecting chronic disease‐related information, the enumerators were instructed to ask the respondents, ‘Have you suffered from any chronic illness/disability in the last 12 months or more?’ (if yes); then, participants were asked a second question: ‘What chronic illness/disability are you suffering from?’ with response options: (1) chronic fever, (2) Injuries/disability, (3) chronic heart disease, (4) respiratory diseases/asthma/bronchitis, (5) diarrhoea/dysentery, (6) gastric/ulcer, (7) blood pressure, (8) arthritis/rheumatism, (9) skin problem, (10) diabetes, (11) cancer, (12) kidney diseases, (13) liver diseases, (14) mental health, (15) paralysis, (16) ear/ENT problem, (17) eye problem or (18) other (specify) (Supporting Information: Appendix Table A1: HIES 2016–2017 questionnaire). Participants responded based on their disease diagnosis, experiences, symptoms of illness and course of treatment. To get valid information, the enumerators also probed where necessary, asked the respondents to show any relevant documents such as prescriptions and test reports, or explained to the respondents about chronic diseases using various case scenarios as outlined in the survey guidelines and instructions.

### 3.1 Study population

**Figure 1:**
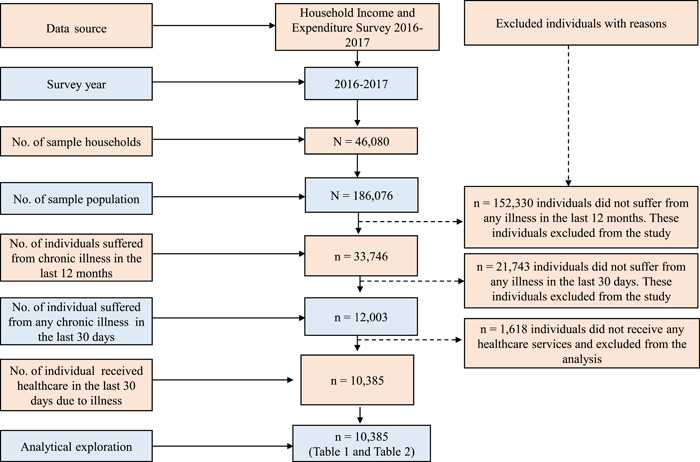
Distribution of study participants

All selected household members were included in the survey. The participants were selected based on the HIES 2016–2017 survey protocol and following the same inclusion criteria: (1) an individual who had suffered from any chronic disease for the last 12 months or more and (2) patients who received any treatment due to chronic disease in the last 30 days. Based on these inclusion criteria, a total of 10,385 patients were selected for the present analysis (Figure 1).

## 4 DEFINITIONS OF STUDY VARIABLES

### 4.1 Outcome measures

Patients' preference for chronic disease healthcare utilization was the primary outcome measure for this study. Participants responded to questions that asked them about their preference for selecting a healthcare provider: ‘why did you choose this provider?’ Response options were recoded as the quality of healthcare services, availability of medical doctors (e.g., availability of male or female doctors in a health facility), affordable healthcare services (i.e., acceptable costs), short distance to health facility (i.e., nearby) and others (e.g., referred by other providers).

### 4.2 Chronic diseases and comorbid conditions

Participants were asked about chronic illness, ‘have you suffered from any chronic illness/disability in the last 12 months or more?’ The prevalence of chronic diseases was assessed based on this question. Individuals who suffered from chronic disease for the last 12 months (or more) and received any treatment due to chronic diseases were the only population considered in this study. The study population was diagnosed with chronic illnesses and conditions such as chronic heart disease, respiratory diseases, chronic gastric or ulcers, high blood pressure, arthritis or rheumatism, diabetes, chronic fever and other diseases. There is no gold‐standard method for measuring comorbidity status among patients with chronic diseases.^28^ A previous review study identified that 21 separate approaches were executed to measure comorbidity status.^28^ The selection of the approach depends on the study research questions, study design, data availability and population studied. The most straightforward approach to measuring comorbidity status is to investigate the distribution of individual comorbid conditions and to treat them independently and/or to combine them by summing the total number of conditions.^29^, ^30^ A single condition count approach was performed to measure comorbidity status in this study. The count of chronic health condition(s) was measured for each patient based on the number of disease exposures and who had been prescribed medication for their illness. It was counted as multiple responses if the patients had multiple chronic conditions. In addition, the principal diagnosis was defined based on patients diagnosed based on their reported first importance. Chronic comorbid conditions (principal diagnosis plus one, two or three or more comorbid conditions) were assessed in this study.

### 4.3 Covariates

Based on the ongoing literature and the authors' own expertise, we selected variables to address the study objective, including healthcare service‐related factors (e.g., type of healthcare service‐inpatient or outpatient, type of health facilities, waiting time for receiving healthcare services, out‐of‐pocket payment and location of consulted healthcare provider) and patients' sociodemographics factors (e.g., gender, age, marital status, education and employment) (Supporting Information: Appendix Table A2).

## 5 STATISTICAL ANALYSIS

The adjusted multinomial logistic regression model was used to identify the potential factors that had an association with the patient's preference for healthcare service. The dependent variable (chronic illness patient's preference for healthcare services) was characterized as a categorical measure in the regression model. An unadjusted analysis was performed using each of the explanatory variables for the following reasons: (1) primary screening of the selection of qualified variables, which were added in the adjusted model, (2) the *χ* ^2^ tests (or one‐way analysis where appropriate) were used to find the association between outcome and explanatory variables. However, the majority of the explanatory variables were categorical with two or more labels; therefore, an unadjusted analysis was performed to find the association between the outcome variable and different categories of explanatory variables. The explanatory variables were included in the adjusted model only if any label of the predictor was significant at a 5% or less risk level in the unadjusted model, which in turn was used to adjust for the associations of other potential confounders. For the explanatory variables, the category found to be least at risk of having patients' preferences for healthcare services related to chronic illness in the analysis was considered the reference category for constructing the relative risk ratio (RRR). Statistical significance was considered at a 5% risk level. All data analyses were undertaken using the statistical software Stata/SE 14 (StataCorp).

## 6 RESULTS

Table 1 shows the participant's characteristics. Of the 10,385 patients with chronic diseases, approximately 50% were female, 51% were married and 70% were unemployed. The majority of the patients were aged between 18 and 45 (81%) years, and around one‐third (37%) had no formal education.

**Table 1:** Distribution of participant's characteristics

Variables	Number of patients	Chronic illness patient's demand for healthcare services					*p* Value
		Short facility distance	Affordable healthcare costs	Availability of doctor	Quality of treatment	Others	
	*n* (%)/mean (SD)	% (95% CI)	% (95% CI)	% (95% CI)	% (95% CI)	% (95% CI)	
*Illness related factors*							
Chronic illness							<.001
Chronic heart disease	869 (8.37)	22.21 (19.57, 25.10)	17.84 (15.43, 20.53)	12.66 (10.61, 15.04)	36.48 (33.34, 39.74)	10.82 (8.92, 13.06)	
Respiratory diseases/asthma/bronchitis	1318 (12.69)	28.30 (25.93, 30.80)	22.15 (19.99, 24.48)	9.86 (8.37, 11.60)	31.26 (28.81, 33.82)	8.42 (7.04, 10.05)	
Gastric/ulcer	1652 (15.91)	34.02 (31.77, 36.34)	23.49 (21.50, 25.59)	9.20 (7.90, 10.69)	25.73 (23.67, 27.89)	7.57 (6.39, 8.95)	
Blood pressure	891 (8.58)	30.64 (27.70, 33.75)	21.89 (19.29, 24.72)	11.45 (9.52, 13.71)	28.84 (25.96, 31.91)	7.18 (5.66, 9.08)	
Arthritis/rheumatism	1439 (13.86)	27.73 (25.47, 30.10)	23.91 (21.77, 26.18)	10.35 (8.88, 12.04)	29.26 (26.96, 31.66)	8.76 (7.40, 10.33)	
Diabetes	654 (6.30)	25.38 (22.19, 28.86)	14.83 (12.31, 17.77)	12.23 (9.93, 14.98)	35.02 (31.45, 38.76)	12.54 (10.21, 15.31)	
Chronic fever	556 (5.35)	26.62 (23.11, 30.45)	18.88 (15.84, 22.36)	12.41 (9.92, 15.42)	31.29 (27.57, 35.28)	10.79 (8.47, 13.66)	
Others	3006 (28.95)	24.92 (23.40, 26.50)	22.52 (21.06, 24.05)	11.78 (10.67, 12.98)	30.14 (28.52, 31.81)	10.65 (9.59, 11.80)	
Number of chronic comorbid conditions, *n* (%)							
Only principal diagnosis	6782 (65.31)	28.59 (27.53, 29.68)	21.13 (20.17, 22.12)	10.53 (9.82, 11.28)	30.49 (29.41, 31.60)	9.26 (8.59, 9.97)	.001
Principal diagnosis + one chronic condition	2455 (23.64)	26.88 (25.17, 28.67)	22.36 (20.76, 24.05)	12.14 (10.90, 13.49)	29.61 (27.84, 31.45)	9.00 (7.93, 10.20)	
Principal diagnosis + two chronic conditions	1148 (11.05)	23.00 (20.65, 25.52)	23.61 (21.24, 26.15)	11.67 (9.94, 13.66)	30.14 (27.55, 32.86)	11.59 (9.86, 13.57)	
*Healthcare service‐related factors*							
Type of healthcare services							
Inpatient care	976 (9.40)	17.73 (15.45, 20.25)	14.24 (12.19, 16.58)	14.86 (12.76, 17.23)	37.70 (34.71, 40.79)	15.47 (13.33, 17.88)	<.001
Outpatient care	9409 (90.60)	28.59 (27.69, 29.51)	22.47 (21.64, 23.32)	10.64 (10.03, 11.28)	29.47 (28.56, 30.40)	8.83 (8.28, 9.42)	
Healthcare facilities							
Pharmacy/dispensary	2461 (23.70)	49.05 (47.07, 51.02)	32.14 (30.32, 34.01)	07.27 (06.31, 08.37)	07.31 (06.35, 08.41)	04.23 (03.50, 05.10)	.001
Doctor's chamber	4040 (38.90)	27.95 (26.58, 29.35)	20.92 (19.69, 22.20)	09.06 (08.21, 09.98)	35.89 (34.43, 37.38)	06.19 (05.49, 06.97)	
Public facility	2024 (19.49)	16.85 (15.28, 18.54)	19.42 (17.75, 21.20)	16.4 (14.85, 18.08)	34.54 (32.49, 36.64)	12.80 (11.41, 14.32)	
Private facility	1405 (13.53)	7.54 (6.27, 9.05)	7.19 (5.95, 8.66)	17.94 (16.02, 20.03)	51.89 (49.27, 54.49)	15.44 (13.65, 17.43)	
Others	455 (4.38)	17.58 (14.35, 21.36)	27.03 (23.15, 31.31)	03.74 (02.33, 05.93)	18.24 (14.95, 22.07)	33.41 (29.22, 37.88)	
Waiting time for receiving healthcare services, average days (SD)	19.75 (13.16)	15.02 (14.63, 15.40)	19.38 (18.87, 19.88)	21.88 (21.13, 22.63)	22.79 (22.29, 23.29)	22.15 (21.23, 23.06)	.002
Average out‐of‐pocket payment, BDT (SD)	3848 (18,513)	1480 (1236, 1724)	1998 (1462, 2535)	4049 (3384, 4714)	6183 (5315, 7051)	7301 (5391, 9210)	.005
Location of consulted healthcare provider							
Rural	5463 (52.60)	40.05 (38.76, 41.36)	26.80 (25.64, 27.99)	09.21 (08.47, 10.00)	16.95 (15.98, 17.97)	06.99 (06.35, 07.70)	<.001
Urban	4922 (47.40)	13.71 (12.78, 14.70)	16.03 (15.03, 17.08)	13.06 (12.15, 14.03)	45.00 (43.62, 46.40)	12.19 (11.31, 13.13)	
*Patients characteristics*							
Age group (years)							
<18	3895 (37.51)	27.29 (25.91, 28.71)	21.44 (20.18, 22.76)	11.27 (10.32, 12.30)	30.04 (28.62, 31.50)	09.96 (09.06, 10.94)	.832
18–35	3240 (31.20)	28.55 (27.02, 30.13)	22.13 (20.73, 23.59)	10.68 (09.66, 11.79)	29.60 (28.05, 31.19)	09.04 (08.10, 10.08)	
36–45	1279 (12.32)	26.90 (24.54, 29.40)	20.33 (18.21, 22.62)	11.42 (09.78, 13.28)	31.67 (29.17, 34.27)	09.70 (08.19, 11.44)	
46–64	1452 (13.98)	26.72 (24.51, 29.06)	22.18 (20.11, 24.39)	11.29 (09.77, 13.03)	31.27 (28.93, 33.70)	08.54 (07.21, 10.09)	
65 or more	519 (5.00)	27.55 (23.88, 31.56)	22.93 (19.51, 26.75)	09.83 (07.54, 12.70)	29.48 (25.71, 33.55)	10.21 (07.88, 13.13)	
Sex							
Male	5189 (49.97)	27.27 (26.07, 28.50)	21.82 (20.71, 22.96)	11.14 (10.31, 12.02)	30.80 (29.55, 32.07)	08.98 (08.23, 09.79)	.403
Female	5196 (50.03)	27.87 (26.66, 29.10)	21.57 (20.48, 22.71)	10.93 (10.11, 11.81)	29.70 (28.47, 30.95)	09.93 (09.15, 10.77)	
Educational status							
No education	3790 (36.49)	27.07 (25.68, 28.51)	21.90 (20.61, 23.25)	10.74 (09.79, 11.77)	31.74 (30.28, 33.24)	08.55 (07.70, 09.48)	.286
Up to primary	3039 (29.26)	27.54 (25.98, 29.16)	22.15 (20.70, 23.66)	11.29 (10.21, 12.46)	29.19 (27.60, 30.83)	09.84 (08.83, 10.95)	
Secondary education	2779 (26.76)	28.64 (26.99, 30.35)	20.98 (19.50, 22.53)	11.16 (10.04, 12.38)	29.36 (27.70, 31.08)	09.86 (08.81, 11.03)	
Higher	777 (7.48)	26.25 (23.28, 29.47)	21.49 (18.74, 24.52)	11.07 (09.05, 13.48)	30.24 (27.11, 33.57)	10.94 (08.93, 13.34)	
Marital status							
Currently married	5302 (51.05)	28.14 (26.95, 29.37)	21.35 (20.27, 22.47)	10.81 (10.00, 11.67)	30.55 (29.33, 31.81)	09.15 (08.40, 09.95)	.681
Never married	3369 (32.44)	26.57 (25.10, 28.08)	22.32 (20.95, 23.76)	11.58 (10.54, 12.70)	29.86 (28.34, 31.43)	09.68 (08.72, 10.72)	
Others (widowed/divorced/separated)	1714 (16.50)	27.77 (25.70, 29.94)	21.53 (19.65, 23.54)	10.68 (09.30, 12.23)	30.05 (27.92, 32.26)	09.98 (08.64, 11.49)	
Employment status							
Yes	3164 (30.47)	27.65 (26.12, 29.24)	21.24 (19.85, 22.70)	11.16 (10.11, 12.30)	31.61 (30.01, 33.25)	08.34 (07.43, 09.36)	.055
No	7221 (69.53)	27.53 (26.51, 28.57)	21.89 (20.96, 22.86)	10.98 (10.28, 11.72)	29.65 (28.61, 30.71)	09.94 (09.27, 10.66)	
Religion status							
Islam	9024 (86.89)	27.67 (26.76, 28.60)	21.93 (21.09, 22.80)	10.84 (10.21, 11.50)	30.35 (29.41, 31.31)	09.21 (08.63, 09.82)	.026
Hinduism	1034 (9.96)	28.14 (25.48, 30.97)	20.79 (18.43, 23.38)	11.51 (9.70, 13.60)	29.11 (26.42, 31.96)	10.44 (08.72, 12.46)	
Others	327 (3.15)	22.94 (18.69, 27.81)	18.04 (14.24, 22.60)	14.98 (11.51, 19.28)	30.89 (26.11, 36.11)	13.15 (09.89, 17.27)	
Total sample	10,385 (100.00)	27.57 (26.72, 28.44)	21.69 (20.91, 22.49)	11.02 (10.43, 11.64)	30.27 (29.40, 31.16)	09.45 (08.91, 10.03)	

*Note*: *p* value was derived using *χ* ^2^ test or one‐way analysis of variance where appropriate.

Abbreviations: BDT, Bangladeshi Taka; CI, confidence interval; SD, standard deviation.

### 6.1 The distribution of chronic disease and utilization of healthcare services

The most prevalent chronic diseases reported by the patients included gastric/ulcer (16%) followed by arthritis/rheumatism (14%) (Table 1). Most of the patients (65%) reported one diagnosed chronic condition, while approximately 24% of the patients had two, and 11% had three or more chronic conditions. Most of the patients (91%) reported utilizing outpatient healthcare services in the past 30 days. A high proportion of the patients visited general practitioner clinics (~40%), followed by pharmacy/dispensary (24%) and public hospitals (20%) for healthcare services. Furthermore, approximately 53% of the patients received services at rural health facilities. The average out‐of‐pocket healthcare expenditure for chronic illness in the last 30 days was 3848 BDT (~47.40 USD; 2017 price year).

### 6.2 Preferences for healthcare services for chronic illnesses

Approximately 30% of patients reported that they preferred quality healthcare services, whereas 28% preferred a short distance to the health facility. Furthermore, 22% of the patients preferred affordable healthcare costs as the main driver, and 11% expressed a preference for the doctor's availability. However, the scenario varied among different chronic illnesses and healthcare services (Table 1). For example, among the patients who received healthcare services from a pharmacy or dispensary, 49% preferred short distances to health facilities, and 32% reported their preference for healthcare affordability.

### 6.3 Correlates of patient preference for chronic disease‐related healthcare services

Table 2 highlights the factors that influence a patient's choice of healthcare services. Among patients with heart disease, quality of healthcare services was 29% more preferred than affordability (RRR = 0.71; 95% confidence interval [CI]: 0.56–0.90; *p* < .001).

**Table 2:** Influencing factors on patient's preference of healthcare services

	Short distance to health facility vs. quality of treatment		Affordable healthcare costs vs. quality of treatment	
Variables	Unadjusted RRR (95% CI)	Adjusted RRR (95% CI)	Unadjusted RRR (95% CI)	Adjusted RRR (95% CI)
*Illness related factors*				
Chronic illness				
Heart disease	0.74*** (0.60, 0.90)	0.79** (0.63, 0.89)	0.65*** (0.53, 0.81)	0.71*** (0.56, 0.9)
Respiratory diseases/asthma/bronchitis	1.10 (0.92, 1.30)	1.03 (0.85, 1.25)	0.95 (0.79, 1.14)	0.9 (0.74, 1.09)
Gastric/ulcer	1.60*** (1.36, 1.87)	1.26*** (1.05, 1.51)	1.22*** (1.03, 1.45)	1.01 (0.84, 1.22)
Blood pressure	1.28*** (1.06, 1.56)	1.19 (0.95, 1.49)	1.02 (0.82, 1.25)	0.97 (0.77, 1.22)
Arthritis/rheumatism	1.15 (0.97, 1.36)	0.92 (0.76, 1.12)	1.09 (0.92, 1.30)	0.92 (0.76, 1.12)
Diabetes	0.88 (0.70, 1.09)	1.00 (0.77, 1.29)	0.57*** (0.44, 0.73)	0.62*** (0.47, 0.81)
Chronic fever	1.03 (0.81, 1.31)	0.89 (0.68, 1.17)	0.81** (0.62, 0.95)	0.70*** (0.53, 0.93)
Others (=ref)	1.00	1.00	1.00	1.00
Number of chronic comorbid conditions, *n* (%)				
Only principal diagnosis (=ref)	1.00	1.00	1.00	1.00
Principal diagnosis + one chronic condition	0.97 (0.86, 1.09)	1.02 (0.89, 1.17)	1.09 (0.96, 1.24)	1.11 (0.97, 1.28)
Principal diagnosis + at least two chronic conditions	0.81*** (0.69, 0.97)	1.18 (0.97, 1.43)	1.13** (1.25, 1.34)	1.39*** (1.15, 1.68)
Number of days after symptoms began did first consultation	0.96*** (0.95, 0.97)	0.99*** (0.98, 0.99)	0.97*** (0.96, 0.98)	0.99*** (0.98, 0.99)
*Healthcare service‐related factors*				
Type of healthcare services				
Inpatient care (ref = outpatient care)	0.48*** (0.40, 0.59)	1.24 (0.96, 1.60)	0.50*** (0.40, 0.61)	0.96 (0.74, 1.23)
Type of healthcare facilities				
Pharmacy/dispensary	6.96*** (4.93, 9.82)	6.88*** (4.80, 9.86)	2.97*** (2.15, 4.09)	3.13*** (2.25, 4.36)
Doctor's chamber	0.81 (0.59, 1.11)	1.01 (0.72, 1.40)	0.39*** (0.29, 0.53)	0.46*** (0.34, 0.62)
Public facility	0.51*** (0.36, 0.71)	0.99 (0.70, 1.41)	0.38*** (0.28, 0.51)	0.59*** (0.43, 0.82)
Private facility	0.15*** (0.10, 0.22)	0.40*** (0.27, 0.58)	0.09*** (0.07, 0.13)	0.17*** (0.12, 0.25)
Others (=ref)	1.00	1.00	1.00	1.00
Waiting time for receiving healthcare services	0.95*** (0.94, 0.96)	0.96*** (0.96, 0.97)	0.98*** (0.97, 0.99)	0.98*** (0.97, 0.98)
Out‐of‐pocket payment	0.98*** (0.97, 0.99)	1.00 (1.00, 1.00)	0.98*** (0.97, 0.99)	1.02*** (1.01, 1.03)
Location of consulted healthcare provider				
Rural (ref = urban)	0.13*** (0.11, 0.14)	0.18 (0.16, 0.21)	0.23*** (0.20, 0.25)	0.32*** (0.28, 0.36)
*Patients characteristics*				
Age of the patients (years)				
<18 (=ref)	1.00	‐	1.00	‐
18–35	1.06 (0.94, 1.20)	‐	1.05 (0.92, 1.19)	‐
36–45	0.93 (0.79, 1.10)	‐	0.90 (0.75, 1.08)	‐
46–64	0.94 (0.80, 1.10)	‐	0.99 (0.84, 1.18)	‐
65 or more	1.03 (0.81, 1.31)	‐	1.09 (0.84, 1.41)	‐
Sex of the patients				
Male (ref = female)	0.94 (0.85, 1.04)		0.98 (0.88, 1.09)	
Educational status				
No education	0.98 (0.80, 1.21)	0.84 (0.66, 1.06)	0.97 (0.78, 1.21)	0.89 (0.70, 1.12)
Up to primary	1.09 (0.88, 1.34)	1.00 (0.78, 1.27)	1.07 (0.85, 1.33)	1.03 (0.81, 1.31)
Secondary education	1.12 (0.91, 1.39)	1.05 (0.83, 1.34)	1.01 (0.80, 1.26)	0.99 (0.77, 1.26)
Higher (=ref)	1.00		1.00	1.00
Marital status				
Currently married (=ref)	1.00	‐	1.00	‐
Never married	0.97 (0.86, 1.08)	‐	1.07 (0.95, 1.21)	‐
Others (widowed/divorced/separated)	1.00 (0.87, 1.16)	‐	1.03 (0.88, 1.20)	‐
Employment status				
No (ref = yes)	1.06 (0.95, 1.18)	1.07 (0.95, 1.22)	1.10 (0.98, 1.24)	1.10 (0.97, 1.25)
Religion status				
Islam	1.23 (0.91, 1.66)	‐	1.24 (0.89, 1.71)	‐
Hinduism	1.30 (0.93, 1.83)	‐	1.22 (0.85, 1.76)	‐
Others (=ref)	1.00	‐	1.00	‐

	Availability of medical doctors vs. quality of treatment		Other attributes vs. quality of treatment	
Variables	Unadjusted RRR (95% CI)	Adjusted RRR (95% CI)	Unadjusted RRR (95% CI)	Adjusted RRR (95% CI)
*Illness related factors*				
Chronic illness				
Heart disease	0.89 (0.69, 1.14)	0.90 (0.70, 1.16)	0.84 (0.65, 1.09)	0.98 (0.75, 1.28)
Respiratory diseases/asthma/bronchitis	0.81 (0.64, 0.92)	0.79*** (0.63, 0.81)	0.76** (0.60, 0.97)	0.85 (0.66, 1.10)
Gastric/ulcer	0.92 (0.73, 1.14)	0.86 (0.68, 1.07)	0.83 (0.66, 1.06)	0.99 (0.77, 1.26)
Blood pressure	1.02 (0.78, 1.32)	1.02 (0.78, 1.32)	0.71*** (0.52, 0.95)	0.85 (0.62, 1.16)
Arthritis/rheumatism	0.91 (0.72, 1.13)	0.85 (0.68, 1.07)	0.85 (0.67, 1.07)	0.95 (0.74, 1.21)
Diabetes	0.89 (0.67, 1.19)	0.89 (0.66, 1.18)	1.01 (0.76, 1.34)	1.11 (0.83, 1.48)
Chronic fever	1.01 (0.75, 1.38)	0.9 (0.66, 1.24)	0.98 (0.71, 1.34)	1.12 (0.80, 1.56)
Others (=ref)	1.00	1.00	1.00	1.00
Number of chronic comorbid conditions, *n* (%)				
Only principal diagnosis (=ref)	1.00	1.00	1.00	1.00
Principal diagnosis + one chronic condition	**1.19** ** **(1.01, 1.39)**	**1.18** * **(1.05, 1.39)**	1.00 (0.84, 1.19)	0.99 (0.82, 1.18)
Principal diagnosis + at least two chronic constitutions	1.12 (0.90, 1.39)	1.13 (0.90, 1.41)	1.27*** (1.02, 1.58)	1.20 (0.95, 1.50)
Number of days after symptoms began did first consultation	0.99 (0.99, 1.00)	1 (0.99, 1.00)	1.02*** (1.01, 1.03)	1.02*** (1.01, 1.04)
*Healthcare service‐related factors*				
Type of healthcare services				
Inpatient care (ref = outpatient care)	1.09 (0.89, 1.34)	1.25 (1.00, 1.72)	1.37*** (1.12, 1.68)	1.48*** (1.17, 1.88)
Type of healthcare facilities				
Pharmacy/dispensary	4.86*** (2.77, 8.51)	5.11*** (2.90, 8.98)	0.32*** (0.22, 0.45)	0.33*** (0.23, 0.48)
Doctor's chamber	1.23 (0.72, 2.10)	1.37 (0.80, 2.35)	0.09*** (0.07, 0.13)	0.10*** (0.07, 0.14)
Public facility	2.32*** (1.35, 3.97)	2.93*** (1.70, 5.04)	0.20*** (0.15, 0.27)	0.22*** (0.16, 0.30)
Private facility	1.69** (1.38, 2.90)	2.38*** (1.38, 4.12)	0.16*** (0.12, 0.22)	0.18*** (0.13, 0.25)
Others (=ref)				
Waiting time for receiving healthcare services	0.99* (0.98, 0.99)	1.00 (0.99, 1.00)	0.99 (0.99, 1.01)	1.00 (0.99, 1.00)
Out‐of‐pocket payment	0.98*** (0.96, 0.99)	1.03*** (1.01, 1.05)	1.00 (0.99, 1.00)	1.00 (1.00, 1.00)
Location of consulted healthcare provider				
Rural (ref = urban)	0.53*** (0.46, 0.61)	0.50*** (0.43, 0.58)	0.66*** (0.57, 0.76)	0.66*** (0.56, 0.78)
*Patients characteristics*				
Age of the patients (years)				
<18 (=ref)	1.00	‐	1.00	‐
18–35	0.96 (0.82, 1.13)	‐	0.92 (0.77, 1.10)	‐
36–45	0.96 (0.77, 1.20)	‐	0.92 (0.73, 1.16)	‐
46–64	0.96 (0.78, 1.19)	‐	0.82 (0.65, 1.04)	‐
65 or more	0.89 (0.64, 1.24)	‐	1.04 (0.75, 1.46)	‐
Sex of the patients				
Male (ref = female)	0.98 (0.86, 1.12)	‐	0.87 (0.76, 1.01)	‐
Educational status				
No education	0.92 (0.70, 1.21)	0.87 (0.66, 1.14)	0.74** (0.56, 0.98)	0.71*** (0.53, 0.94)
Up to primary	1.06 (0.80, 1.39)	1.00 (0.75, 1.32)	0.93 (0.70, 1.23)	0.87 (0.65, 1.16)
Secondary education	1.04 (0.78, 1.37)	1.01 (0.76, 1.34)	0.93 (0.70, 1.23)	0.89 (0.66, 1.18)
Higher (=ref)	1.00	1.00	1.00	1.00
Marital status				
Currently married (=ref)	1.00	‐	1.00	‐
Never married	1.10 (0.94, 1.27)	‐	1.08 (0.92, 1.27)	‐
Others (widowed/divorced/separated)	1.00 (0.83, 1.22)	‐	1.11 (0.91, 1.36)	‐
Employment status				
No (ref = yes)	1.05 (0.91, 1.21)	1.05 (0.90, 1.21)	1.27*** (1.08, 1.49)	1.24*** (1.05, 1.46)
Religion status				
Islam	0.74 (0.52, 1.04)	‐	0.71 (0.49, 1.03)	‐
Hinduism	0.81 (0.55, 1.22)	‐	0.84 (0.55, 1.28)	‐
Others (=ref)	1.00	‐	1.00	‐

*Note*: ***, ** and * denoted significance level at 0.1%, 1% and 5%, respectively.

Abbreviations: CI, confidence interval; ref, reference group; RRR, relative risk ratio.

Similarly, diabetic patients exhibited a 38% higher preference for quality rather than affordability of healthcare services (RRR = 0.62; 0.47–0.81; *p* < .001). However, patients with three or more chronic comorbid conditions were 1.39 times more likely to prefer affordability over the quality of healthcare services (RRR = 1.39; 1.15–1.68; *p* < .001). Patients who received healthcare services from a public facility reported a higher preference for the availability of medical doctors or consultants (RRR = 2.93; 1.70–5.04; *p* < .001) than those receiving health care from private service providers. In addition, patients who received healthcare services from pharmacies or dispensaries were significantly more likely to prefer a short distance to a healthcare facility (RRR = 6.99; 4.80–9.86; *p* < .001) or affordability of healthcare services (RRR = 3.13; 2.25–4.36; *p* < .001), rather than the quality of the healthcare services and availability of doctors.

## 7 DISCUSSION

Chronic diseases among adults are becoming a significant health concern in many LMICs, including Bangladesh. This is the first study to focus on patient preferences for healthcare services for chronic disease management, using a recent nationally representative HIES. This study provided evidence of patient preference for the utilization of healthcare services in chronic disease management. The top four dimensions of patient preference for health care in order of preference were quality of treatment (30.3%), short distance to health facilities (27.6%), affordability of health care (21.7%) and availability of doctors (11.0%). The severity of the disease and the characteristics of healthcare providers were the most important contributing factors in patients' preferences and decisions to seek healthcare services.

This study showed that patients with heart disease were more likely to prefer quality health care than healthcare affordability or a short distance to a health facility. This is in agreement with previous studies, which have also documented that chronic heart disease patients are more likely to prefer quality healthcare services.^31^, ^32^, ^33^ Several attributes might influence a patient's preference for quality healthcare services. For instance, heart disease may be associated with both acute episodes and high levels of long‐term adverse events (e.g., mortality and disability).^34^, ^35^, ^36^ Such patients may require curative care as a component of a continuous, coordinated care model, covering both primary and postacute hospital care.^34^ Moreover, due to the high disease burden and prolonged treatment, continuous use of healthcare resources (e.g., specialist consultation, diagnostic and medicine) may lead to a higher economic burden for households, individuals and society.^35^, ^36^, ^37^ From a healthcare service providers' perspective, examining patients' experience and satisfaction with healthcare services could identify whether services are of an acceptable standard or highlight areas for potential quality improvement.^38^ Existing research has revealed that a positive experience with healthcare professionals or other medical staff is directly related to patient preference for healthcare quality during hospitalization or course of treatment.^39^ Other studies have indicated the importance of having confidence in the expertise and attentiveness of doctors or nurses,^40^, ^41^ healthcare provider's interpersonal communication skills and behaviours,^42^ although shorter waiting times have also been mentioned/raised.^43^

In the present study, one of the most influential aspects of a patient's healthcare‐seeking behaviour was the healthcare provider's location; the shorter the distance to the healthcare facility the more likely the patient uptake of the healthcare services. It is plausible that patients may be unwilling or unable to travel long distances to access medical expertise or treatment, particularly if the nature of the chronic illness requires frequent appointments. This finding is supported by previous studies which reported that distance to healthcare facilities was a potential barrier to accessing healthcare services in LMICs.^44^, ^45^ Patients' educational level, employment status and chronic comorbid conditions were also identified as significant determinants influencing their healthcare‐seeking preferences. This may be due to patients with chronic illnesses being more likely to prioritize treatment, management and quality of life over other life choices they may face.^46^ In addition, patients with multiple chronic conditions may require medical care from several healthcare providers across various locations throughout the year. This may be attributed to seeking quality care from experienced or specialist medical professionals, and superior diagnosis and treatment facilities, which has also been identified elsewhere.^47^

Although this is the first study to investigate patient preference for healthcare services to manage chronic diseases among adults in Bangladesh, the extension and transferability of our findings to contexts beyond the study population should be handled with caution because of our study limitations. For instance, due to the cross‐sectional nature of the study, causality cannot be inferred. Furthermore, these findings may be subjected to some level of bias as data on the main variables of interest were self‐reported (i.e., illness, utilization and expenditure), thereby risking recall bias. However, the relatively short recall period (i.e., the last 30 days) of the household income and expenditure survey strives to reduce this potential bias.

### 7.1 Implications for policy and practice

Our study highlights the significance of patient preferences for healthcare utilization. This study provides timely findings to address health inequities linked to sociocultural and economic factors in Bangladesh. Understanding patients' preferences in chronic disease management is critical to achieving the Sustainable Development Goal (SDG) target 3.4, which focuses solely on reducing premature mortality from noncommunicable diseases by a third by 2030 relative when compared to 2015 as a baseline. Bangladesh is a signatory to the SDGs and the Colombo declaration (strengthening health systems to accelerate delivery of NCD‐related services at the primary healthcare level),^48^ providing evidence that informs culturally competent NCD prevention and treatment approaches through tailored and responsive health financing, and expenditure policies will contribute significantly to achieving the SDG target 3.4. However, progress in implementing strategies to meet international targets related to NCDs remains slow.^9^, ^11^, ^49^

Additionally, Bangladesh lacks a national surveillance programme focused on chronic diseases and does not have any integrated community public health programme that regularly monitors chronic diseases.^11^ An established disease management plan should consider the adequacy, accessibility, affordability and quality of services.^48^ Most importantly, the factors influencing patient preferences for healthcare services may not have been considered when developing the chronic disease management policy.^11^ Therefore, policymakers and public health practitioners should consider patient preferences regarding healthcare utilization in managing chronic diseases.

## 8 CONCLUSION

Our study findings highlighted that patient preferences for healthcare services in chronic disease management were significantly associated with disease severity and healthcare providers' attributes. Therefore, policymakers and public health practitioners should consider patient preferences for managing chronic conditions within the national strategic frameworks to improve service provision, equitable distribution and uptake of the services.

## AUTHOR CONTRIBUTIONS

Rashidul A. Mahumud conceptualized the study, directed the data analysis and wrote most of the manuscript. Rashidul A. Mahumud performed the statistical analysis under the supervision of Andre M. N. Renzaho, Khorshed Alam and Asaduzzaman Khan. Marufa Sultana, Satyajit Kundu, Md. A. Rahman and Mostafa Kamal wrote portions of the manuscript. Rashidul A. Mahumud, Khorshed Alam, Andre M. N. Renzaho, Sabuj K. Mistry, Joseph K. Kamara, Md. G. Hossain, Mohammad A. Ali and Asaduzzaman Khan contributed to the study design, data interpretation and edited the manuscript. All named authors contributed to critically reviewing the revised initial draft of the manuscript. All authors read and approved the final draft.

## CONFLICT OF INTEREST

The authors declare no conflict of interest.

## ETHICS STATEMENT

This study has been prepared using a secondary de‐identified and fully anonymized cross‐sectional data set from the HIES conducted during 2016–2017 by the Bangladesh Bureau of Statistics. Therefore, no ethical approval was not required for this study.

## Supporting information

Supporting information.

Supporting information.

Supporting information.

## Data Availability

The data that support the findings of this study are available on request from the corresponding author.
