# Supplementary material for: The burden of chronic diseases and patients' preference for healthcare services among adult patients suffering from chronic diseases in Bangladesh
Source: Health Expect. 2022 Oct 20;25(6):3259–73. doi: 10.1111/hex.13634 (PMC9700186; doi:10.1111/hex.13634)
Supplement: Supplementary file 1 — Supporting information. [file HEX-25--s001.docx]

HIES Q-1

**Appendix Table A1: HIES Survey Instruments (Word file created from pdf file)**

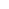


T A B L E O F C O N T E N T S

Order of Sections

Page No.

Order of Sections

Page No.

1

HOUSEHOLD INFORMATION ROSTER

8

OTHER ASSETS AND INCOME

PART A:

PART B:

HOUSEHOLD INFORMATION (with DISABILITY)

EMP LOY MENT INFORMATION

1

2

PART A: OTHER PROPERTY AND ASSETS

PART B: OTHER INC O ME

25

26

PART C: SOCIAL SAFETY NETS PROGRAMME

3-4

PART C: MIGRATION AND REMITTANCES

PART D: MICRO CREDIT

27-28

29

2

EDUCATION

PART A: LITERACY AND EDUCATIONAL ATTAINMENT

PART B: CURRENT ENROLLMENT

5

9

CONS UMP TION

6-7

PART A: DAILY CONS UMP TION

DAY 1

DAY 2

DAY 3

DAY 4

DAY 5

DAY 6

DAY 7

30-31

32-33

34-35

36-37

38-39

40-41

42-43

3

4

HEALTH

PART A: ILLNESSES AND INJ URIES

8-11

ECONOMIC ACTIVITIES AND WAGE EMPLOYMENT

PART A: ACTIVITIES

12

13

PART B: WAGE EMPLOYMENT

5

6

NON-AGRICULTURAL ENTERPRISES

14-15

PART B: WEEKLY CONSUMPTION (DAY 1-7)

44

45-46

47-48

49-50

51-52

53-54

55-56

57-58

59

PART A: DAY 8

HOUSING

DAY 9

PART A: HOUSING INFORMATION

PART B: SHOCKS AND COPING

16

17

DAY 10

DAY 11

DAY 12

7

AGRICULTURE

DAY 13

PART A: LANDHOLDING

18

DAY 14

PART B: CROP PRODUCTION

PART C: NON- CROP ACTIVITIES

LIVESTOCK AND POULTRY

LIVESTOCK PRODUCTS

19-20

PART B: WEEKLY CONSUMPTION (DAY 8-14)

PART C: MONTHLY NON-FOOD EXPENDITURE

PART D: ANNUAL NON-FOOD EXPENDITURE

PART E: INVENTORY OF CONSUMER DURABLE GOODS

60-61

62-65

66

21

21

22

22

23

24

FISH FARMING AND FISH CAPTURE

FARMFORESTRY

PART D: EXPENSES ON AGRICULTURAL INPUTS

PART E: AGRICULTURAL ASSETS


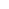

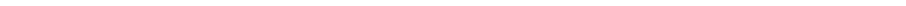

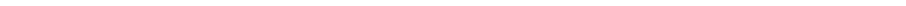

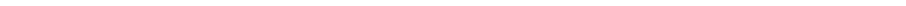

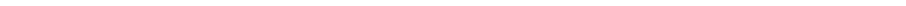

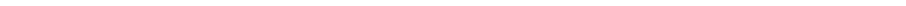

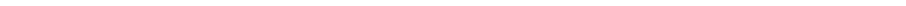

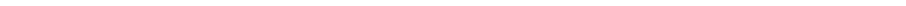

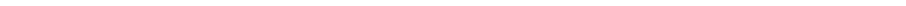

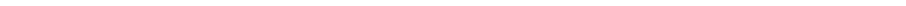

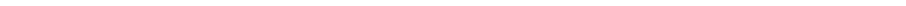

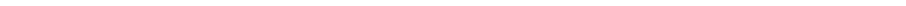

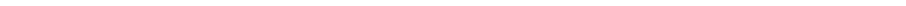

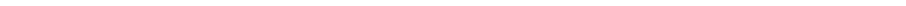

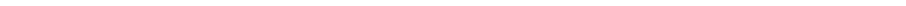

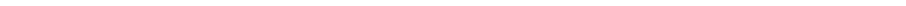

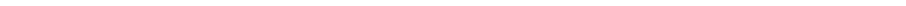

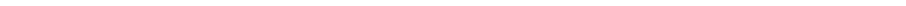

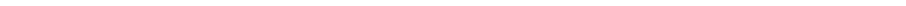

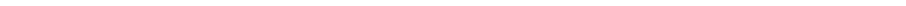

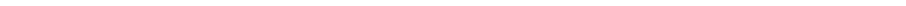

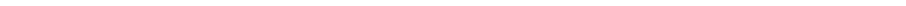

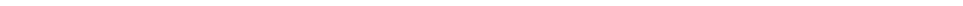

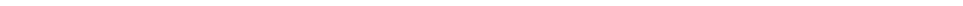

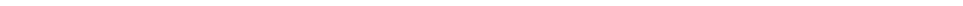

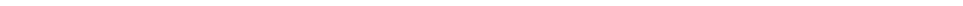

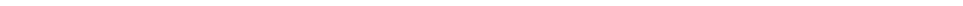

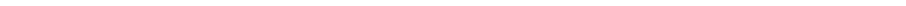

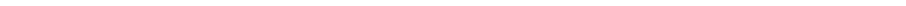

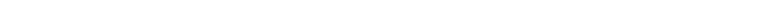

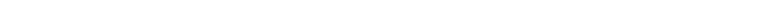

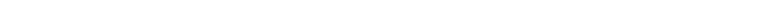

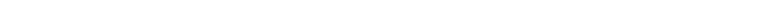

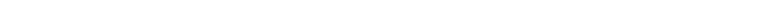

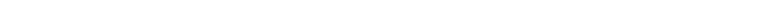

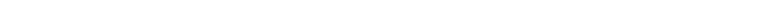

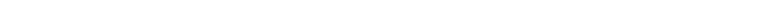

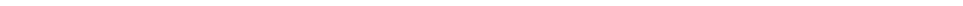

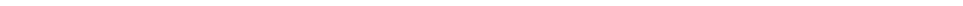

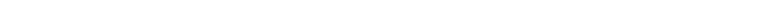

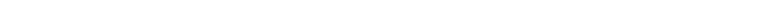

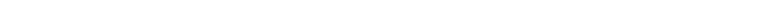

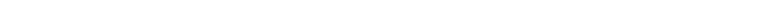

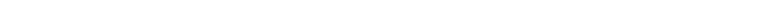

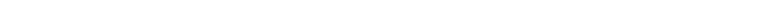

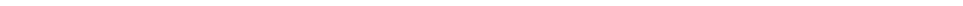

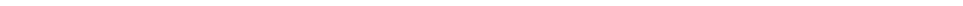

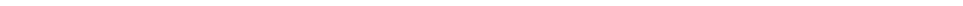

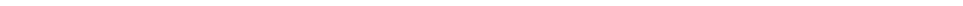

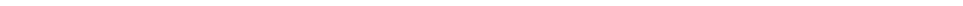

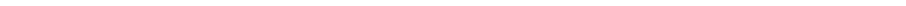

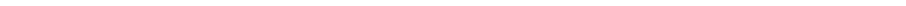

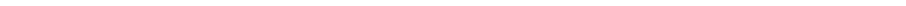

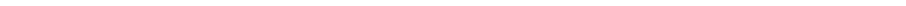


HIES 2016

SECTION 1: HOUSEHOLD INFORMATION ROSTER

PART A: HOUSEHOLD INFORMATION(all members listed in the roster)

DISABILITY

1

2

3

4

5

6

7

8

9

10

11

12

13

14

15

16

17

Sex

Relationship of members with the head of

the household

Age

Religion

Marital status If ever Earner Whether Why did (name)

Whether Main use of the

(name) mobile

has a

Does

(name)

have

difficulty difficulty

for

Does

(name)

have

Does

(name)

have

Does

(name)

have

Does (name) Does (name)

married

(name)

was

abroad

more than

6 months

during

return to the

householdfrom

abroad?

have

difficulty(with difficulty

self care communicati

ng; for

washing all example

have

mobile?

difficulty

difficulty

hearing,

even if

he/she is or any

forwalking remember- such as)

orclimbing ing or

01 Head

09 Father/Mother- WRITE AGE 1 Islam

1 Currently

Married

seeing,

even if

02 Husband/ wife

03 Son/Daughter

in- law

IN FULL

YEARS

2 Hinduism

Age at

1st

marriage

1 Lose job

concent-

rating?

overor

understandin

10 Brother/Sister- in

law

3 Buddhism 2 Never

1 Yes last 5

2 No years?

2 Due to illness

1 Communicating he/she is wearing a other

dressing,

feeding,

g or being

under-stood?

1 Male

04 Spouse of

Son/Daughter

4 Christianity

Married

3 End of

wearing hearing

glasses? aid?

physical

move-

ment?

>>Q 7

employment

toiletingetc?

2 Female

11 Otherrelative,

specify

5 Other

(specify)

2 Getting

information

related to work

05 Grandchild

3 Widowed

4 Divorced

5 Separated

4 Disagreement

withauthorities

1

2

Yes

No

06 Father/Mother

07 Brother/Sister

12 Servant

WRITE "00"

FOR LESS

THAN 1

1

2

Yes

No

13 Employee

5 Homesick

>>Q 12 3 Transferring

08 Niece/Nephew 14 Other(specify)

>>Q 10 6 Due to

Economic

money

(one) YEAR

4 Accessingthe

internet

>10 y/o only

Recession

7 Other(specify)

Code

Code

Code

Code

Code

Code

01

02

03

04

05

06

07

08

09

10

11

12

13

14

15

Codes of Question No: 12, 13, 14, 15, 16 and 17

1-No Difficulty

2-Yes, Some Difficulty

3-Yes, Severe Difficulty

4-Yes, Can'tsee/hear/walk/remember/

selfcare/communicate at all

1A

Page 1


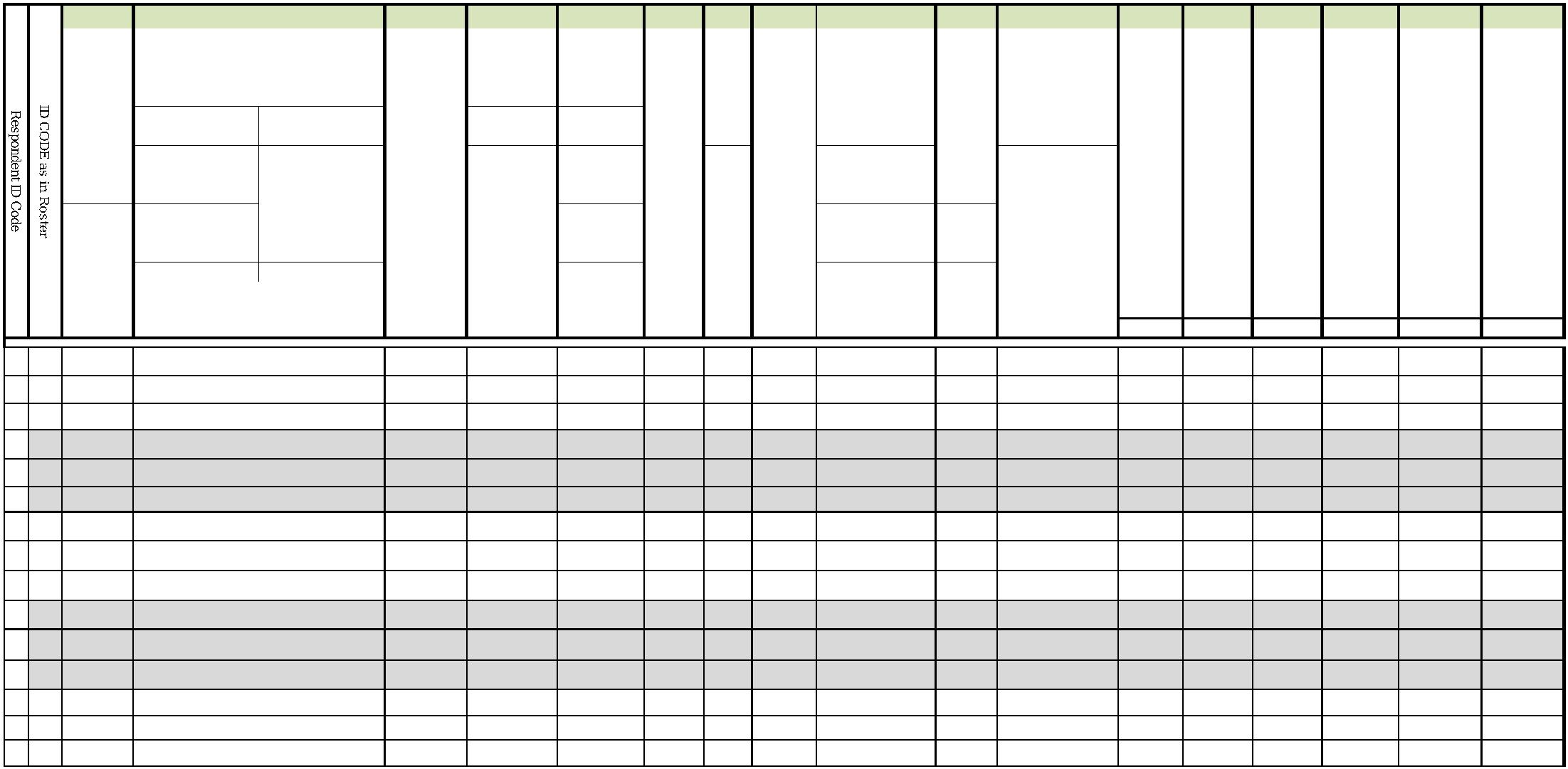

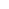

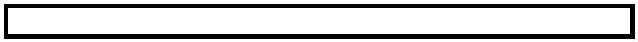

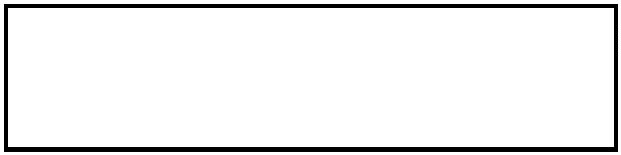


HIES 2016

SECTION 1: HOUS EHOLD INFORMATION ROSTER

PART B: EMP LOY MENT INFORMATION (ALL PERSONS 5 YEARS AND OLDER)

1

2

3

4

Did you work for livelihood Were you available Did you looking for work

Why were you not available/ did you not looking for work?

during the past 7 days?

for work during the during the past 7 days?

past 7 days?

01 Engaged in domestic work

02 Housewife

03 Student

04 Too old/ retired

05 Too young

1

2

Yes >>Next person

No

1

2

Yes

1

2

Yes >>Next person

No

No >>Q4

06 Temporarily sick

07 Disabled

08 Waiting to start new job

09 No work available

10 On leave/looking forjob/business

11 Other(Specify)

01

02

03

04

05

06

07

08

09

10

11

12

13

14

15

1B

Page 2


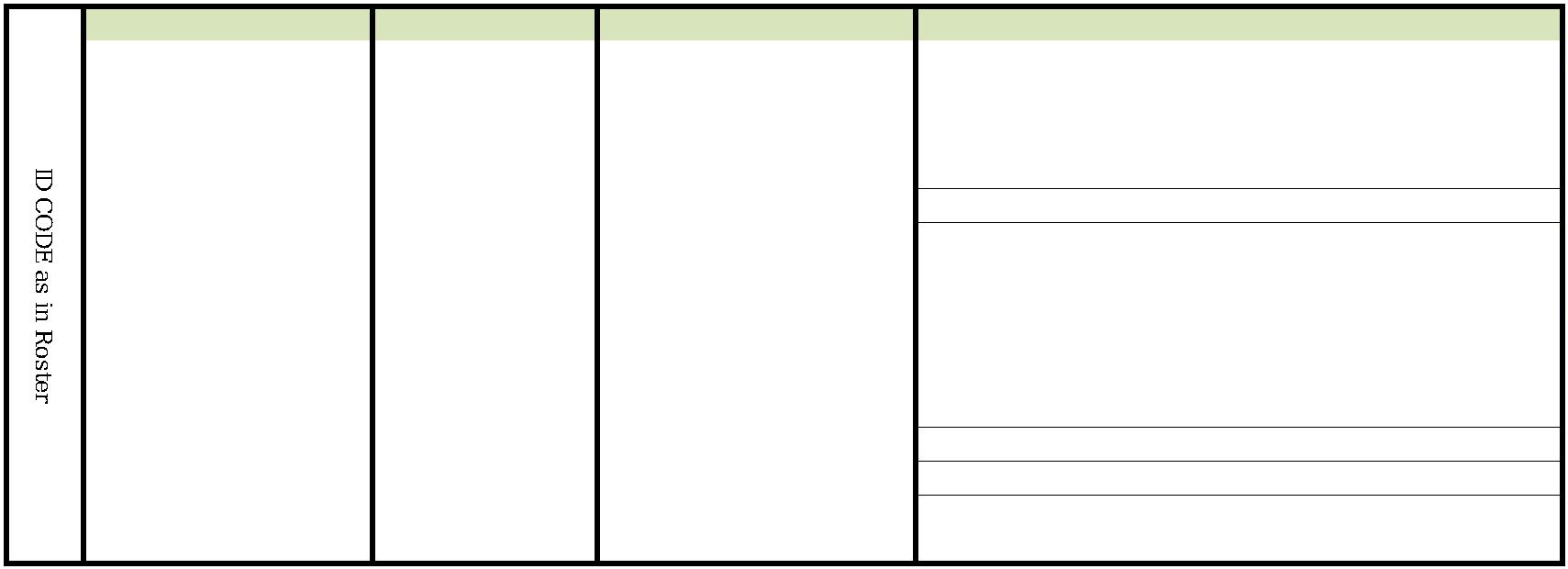

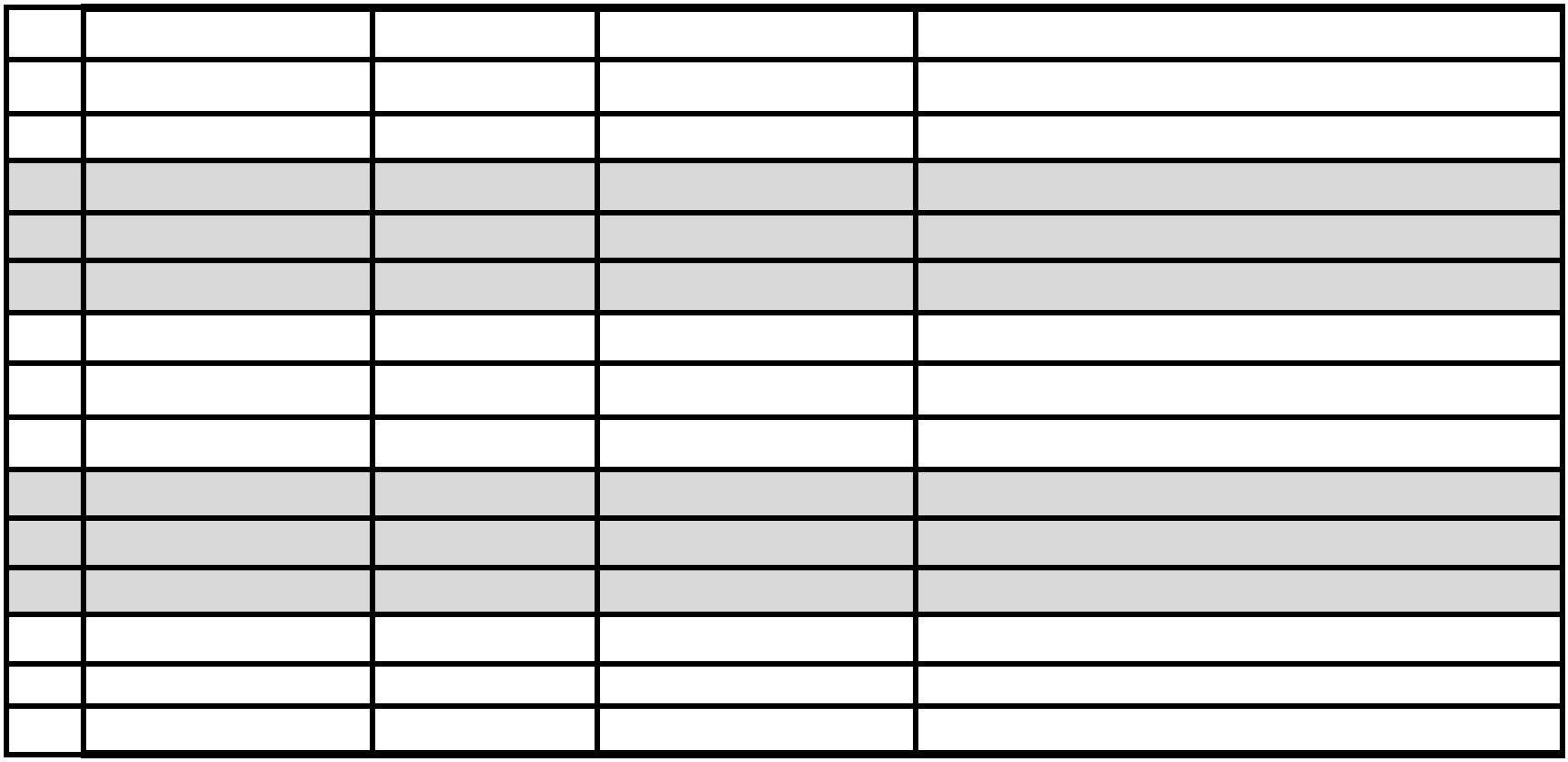


SECTION 1: HOUSEHOLD INFORMATION ROSTER

^PART C: SOCIAL SAFETY NETS PROGRAMME (ALL PERSONS 5 YEARS & OLD^H^ER^IE^)^ S 2016

Now we would like to know from which Social Safety Net Programs, yourhousehold or any member of yourhousehold received assistance during the last 12 months. We would also like to know which benefits

yourhousehold have received from Social Safety Net Programs and quantity of that assistance etc.

Collect data for last one year. In case any memberparticipates in multiple programs report in multiple row for each program the member is participating and carry over the respondent ID as in roster.

1

2

3

4

5

6

7

8

Is (name)

currently

enrolled /

has

received

any

assistance months?

from any

safety net

If yes, in

which

program

(name) has programme? last

Included in

the last 12

When did

(name) enlist (name)

in this received

When did

How much was (name)

receive in cash/in kinds in last (name)

Did

In-kind goods

What was/were

the payment

method(s)?

Which goods?

payment?

received

payment

in-kind?

E

F

COLUMN A: look for the item code at the bottom of the page (CODE 2)

For Time unit

Cash

payment

from this

program?

how

many

months

or

1

2

3

Cash

For how Time unit

many

COLUMN B: write the quantity

1

kg

1. Day

1 yes

2 no

COLUMN C: Code of unit of

measurement:

days?

months

2

3

liter

1. Bank A/C

2. Post office

3. BKASH

program in

last 12

months?

or days?

ENUMERATO

1. Day

number

R: LIST ALL

PROGRAMS

RECEIVEDBY

THE PERSON

FIRST

IF NOT

RECEIVE

D YET

WRITE

"99" IN MM

AND

"9999" IN

YY AND

>> Q.11

2. Month

>>Q.8 COLUMN D: Total value in Takas of the in-kind good

2. Month

IF

NOTHIN

G

WRITE

"0" AND

>>6

1

2

3. One-

shot

3. One-

shot

4. MOBILE

BANKING

1 Yes

2 No

A

B

C

D

A

B

C

D

5. Hand to hand

6. Other

>> Next

person

CODE 1

1st

2nd

MM

YY

MM YY

TAKA Number

Code

Nb

Code

method method

01

02

03

04

05

06

CODE 1: Safety Net Programme code

CODE 2: In-Kind good code

1 Ananda School (ROSC) [Cash/kind]

2 Stipend for Primary Students

3 School Feeding Program

13 Ration for Shaheed Family and Injured Freedom Fighters

14 AllowancesforDistressedCulturalPersonalities/Activists

15 Allowances for the Financially Insolvent Disabled

16 Vulnerable Group Development (VGD)

26 Rural Employment and Road Maintenance Programme 1 Rice

(RERMP)

12 Biscuit

2 Fortifiedrice

13 Clothes/blanket

14 Cook-stove

27 HousingSupport

3 Wheat

4 Stipend for Secondary and higher students (boys/girls)

5 Stipend for Dropout Students

28 AgricultureRehabilitation

4 Maize

15 Cow/ox/bull/buffalo

16 Goat/sheeps

17 Chicken/duck(Poultry)

18 Boat/engineboat

19 Rickshaw/van/cart

20 Tin

17 Vulnerable Group Feeding (VGF)

29 One Household One Farm

30 Targeted Ultra Poor (TUP) (BRAC)

31 CharLivelihoodProject

5 Flour

6 StipendforDisabledStudents

18 General Relief Activities

6 Fortifiedflour

7 Old Age Allowance

19 Gratuitous Relief (GR)- Food/ Cash

7 Parchedrice,

flattenedrice

8 Widow/Deserted/DestituteWomenAllowances

20 Allowance for Beneficiaries in CTG-Hill Tracts Area

21 Food Assistance in CTG-Hill Tracts Area

22 Employment Generation Programme for the Ultra Poor

23 Food/ Cash For Work (FFW/CFW)

32 Economic Empowerment for the Poor/Shiree

33 Urban Partnership for Poverty Reduction (UPPR)

34 ShouhardoProgram

9 Maternity Allowance Programme for the Poor Lactating Mothers

10 Maternal Health Voucher Scheme

8 Pulse

9 Edible oil

10 Powder milk

21 Latrine

11 HonorariumforInsolventFreedomFighters

35 Nabojibon Program (Save the Children)

36 ProsharProgram(ACDIVOCA)

88 Other(specify)

22 Housing

12 Honorarium &Medical Allowances for Injured Freedom Fighters

24 Test Relief (TR) Food (cash)

88 Other(specify)

11 Packagedliquid

milk

25 Rural Employment Opportunity for Public Asset (REOPA)

1C1

Page 3


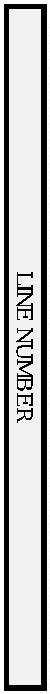

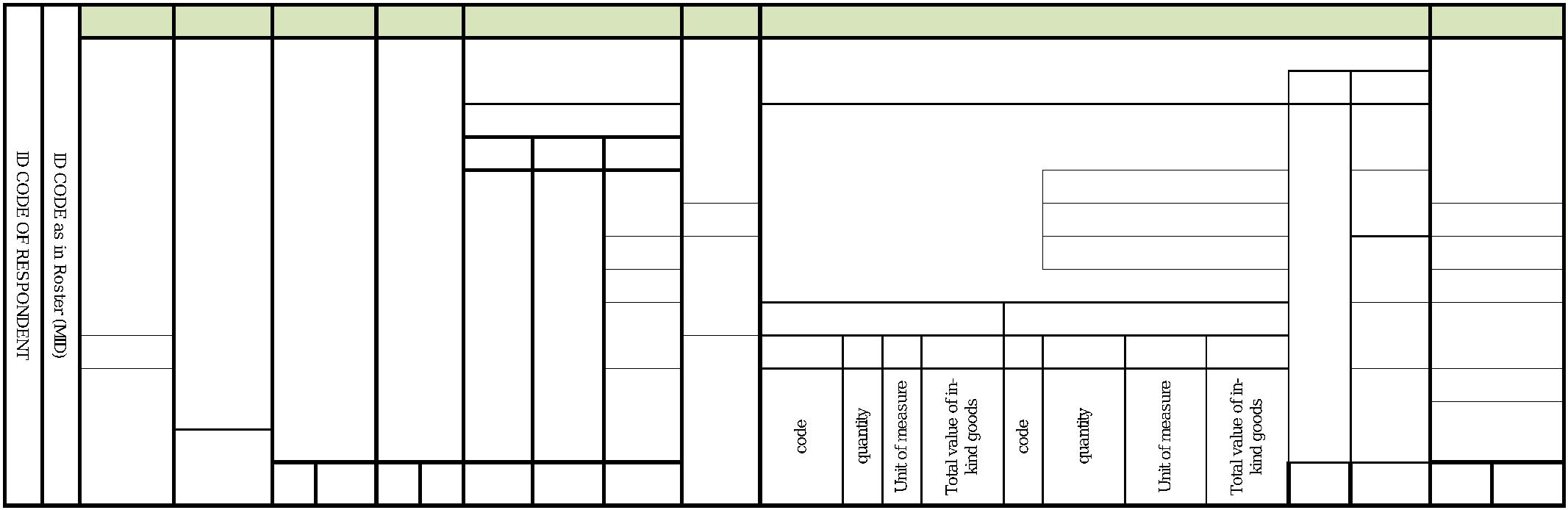

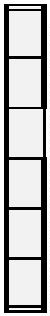

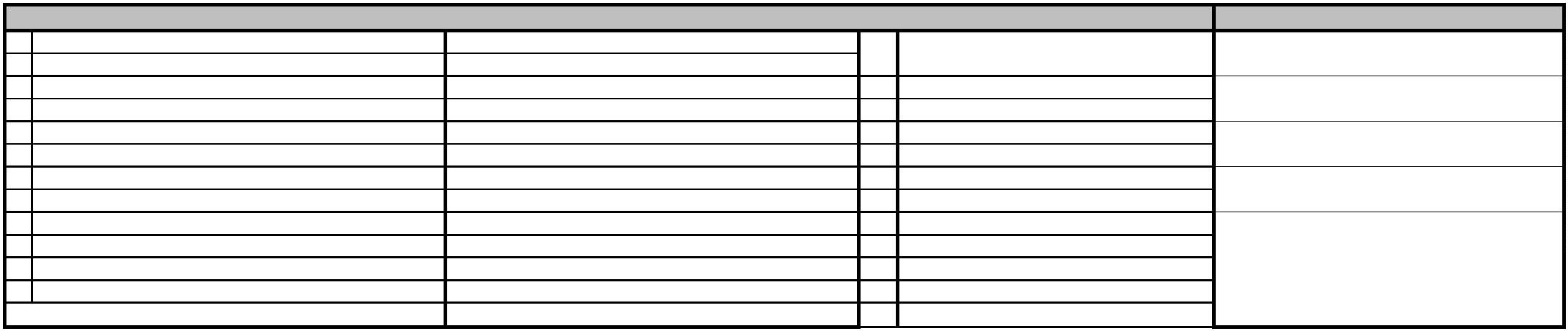

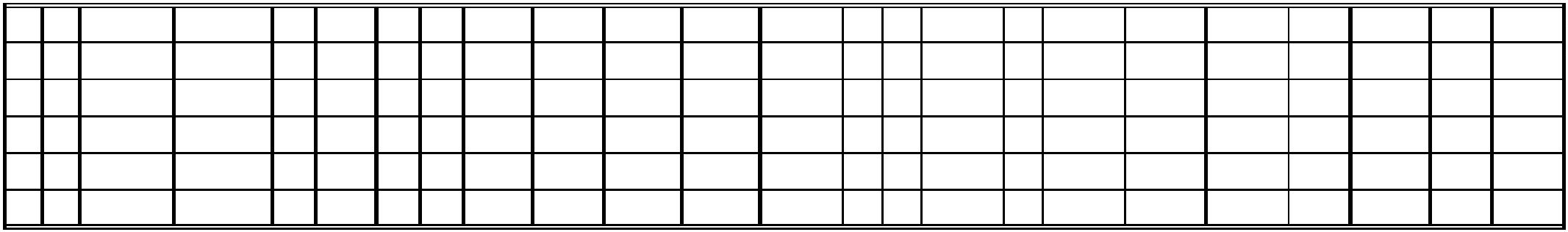


SECTION 1: HOUSEHOLD INFORMATION ROSTER

PART C: SOCIAL SAFETY NETS PROGRAMME (ALL PERSONS 5 YEARS &OLDER)

HIES 2016

9

10

11

12

13

14

15

16

How much was (name) entitlement in cash/in kinds for the lastpayment How much was (name) receive in cash/in kinds in last 12 months?

you have received?

For what

criteria your knowabout

(name)/

Did (name)

How did What was Did (name) How

(name) the had to pay much did

this safety net know selection any money (name)

household program

is selected before

for this

aboutthis process: to be

program? How did

participation? (FIRST (name)

had to

included in pay?

this

COLUMN A: look for the itemcode at the bottom of the

page (CODE 2)

COLUMN A: look for the itemcode at the bottom of the page (CODE 2)

Cash

Cash

A-1

A-1

Amount

received

program?

SOURCE) selected in program?

COLUMN B: write the quantity

1

kg

COLUMN B: write the quantity

1

kg

this

program?

COLUMN C: Code of unit of

measurement:

COLUMN C: Code of unit of

measurement:

2

3

liter

2

3

liter

number

number

COLUMN D: Total value in Takas of the in-kind good

COLUMN D: Total value in Takas of the in-kind good

1

2

Yes

No

1

2

1

2

1

2

Yes

No

WRITE "0"

IF

WRITE "0"

IF

A

B

C

D

A

B

C

D

A

B

C

D

A

B

C

D

NOTHING

NOTHING

>>Q 14

>>Next row

Code 3

TAKA

TAKA

1st 2nd

Code

Code 4

Code 5

Code

TAKA

01

02

03

04

05

06

CODE 2: In-Kind good code

Code 3: Criteria of selection

Code 4: Source of info

Code 5: How selected

01 Appliedandselected

1 Rice

12 Biscuit

01 Householdheadisfemale/widow/divorced/separatedorhusband 11 The household members cannot have 3 square meals per day.

isphysically/mentallyhandicapped.

01 Miking

2 Fortifiedrice

3 Wheat

4 Maize

13 Clothes/blanket

02 Mobile message

03 Newspaper

02 Persued/someonereferredandselected

03 Selectioncommitteeselected

04 NGOselected

14 Cook-stove

02 Householdheadisphysically/mentally

12 The household is suffering food insecurity due to disaster.

15 Cow/ox/bull/buffalo

16 Goat/sheeps

03 Household has no capable/able bodied adult male.

04 Household head‘s main occupation is ag./non-ag. day labor.

04 Radioprogram

05 TVprogram

5 Flour

13 The household has school going children.

88 Other(specify)

6 Fortifiedflour

7 Parchedrice,flattenedrice

17 Chicken/duck(Poultry)

18 Boat/engineboat

05 Any Household member (other than HHhead) has main

occupationofag./non-agdaylabororbeggar.

14 The household has school drop-out children going to school

now.

06 Chairman/secretary/ Member of the union parishad

committee/guard

99 Don’tknow

19 Rickshaw/van/cart

20 Tin

06 Any child of the household engaged in child labor.

15 The person is a insolvent freedomfighter.

07 Teacher, member of school Committee

8 Pulse

07 The household has land less than 50 decimal of land or land less. 16 The person is a injured freedomfighter.

17 The person is a distressed cultural personality/ activists.

08 Localinfluentialperson

09 Friend/family/relative/neighbor

9 Edible oil

21 Latrine

10 Powdermilk

11 Packagedliquidmilk

22 Housing

08 Thehouseholddoessharecropping.

18 Beneficiaryisaphysically/mentallyhandicappedperson.

19 Beneficiary is a distressed &old aged person.

10 NGO warkers

88 Other(specify)

09 The household has almost no productive

88 Others(specify)

10 Household members are ineligible (extreme poor, no savings, etc) 20 Beneficiaryisawidow/divorced/separatedwomen.

to get credit.

88 Other(specify)

99 Don'tknow

1C2

Page 4


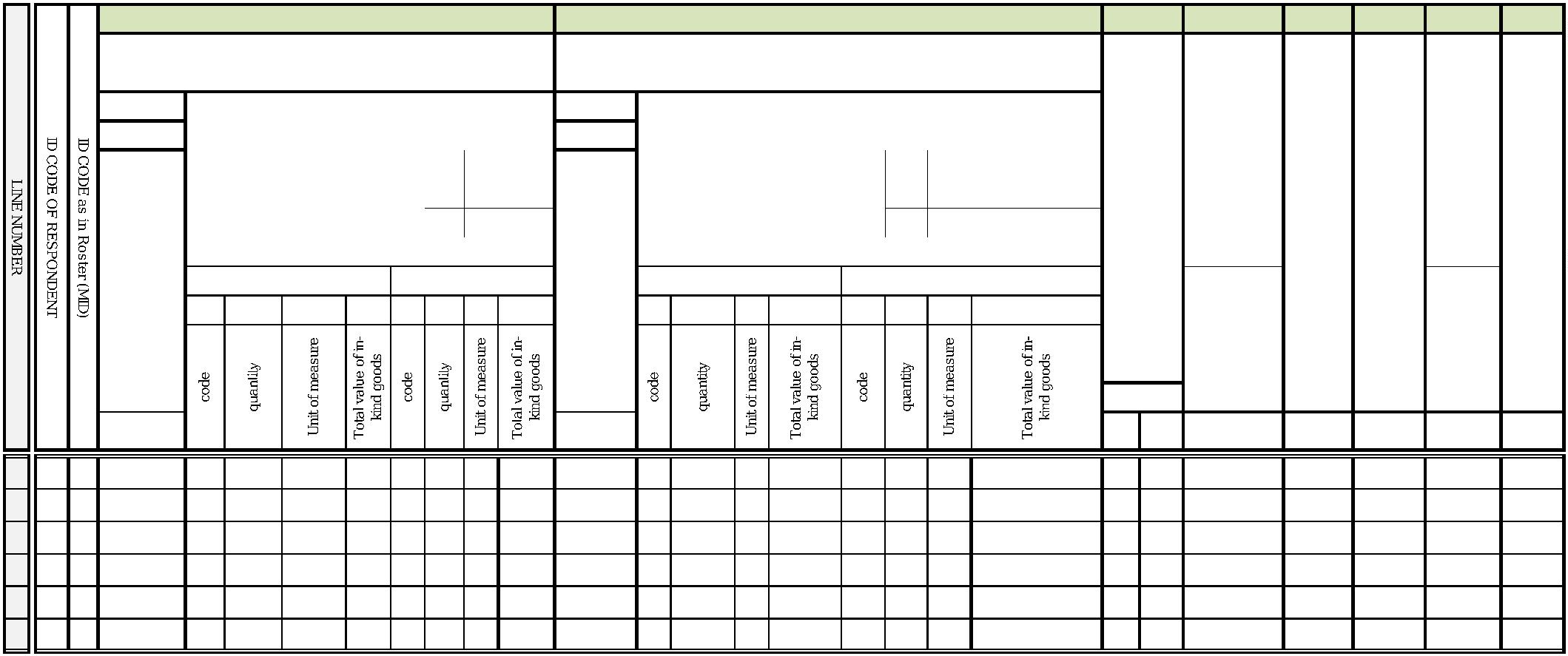

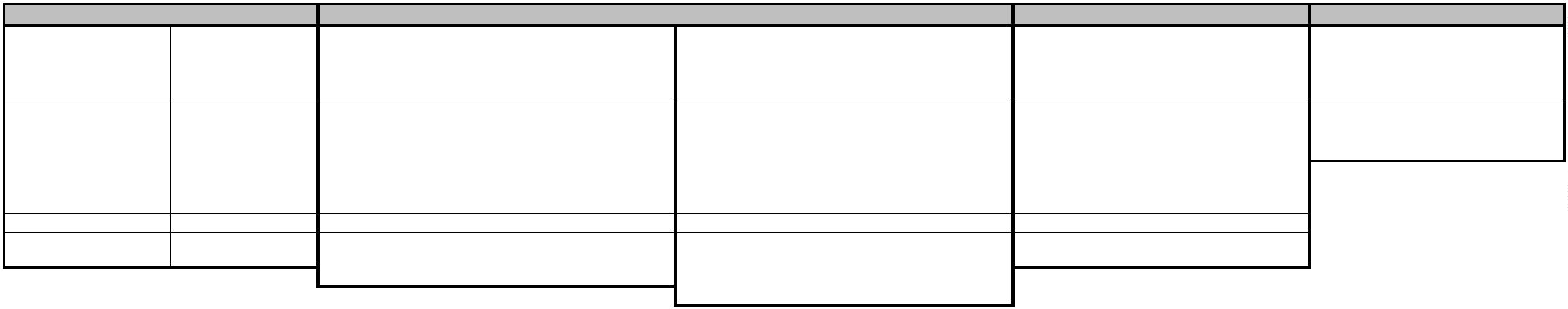

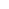


HIES 2016

SECTION 2: EDUCATION

PART A: LITERACY AND EDUCATION ATTAINMENT (ALL PERSONS 5 YEARS AND OLDER)

1

Can you

read a

letter?

2

Can you

write a

letter?

3

Have you ever

attended

4

5

Where did you do last study/are you currently

studying?

6

7

What was the highestclass that you completed?

What type of school/ institution did you Was the last studies abroad

lastattended/ are you currently

attending?

(outside Bangladesh)?

education?

00

01

02

03

04

05

06

07

08

09

10

11

No class passed/pre-schooling

1 Formal School

2 FormalCollege

3 FormalUniversity

4 Madrasha

Class 1

12 Vocational

Class 2

13 Nursing

Class 3

14 TechnicalEducation

15 Graduate/equivalent

16 Medical

1 Government

1 Yes

2 No

1 Yes

2 No

1 Yes

2 No

1 Yes

Class 4

5 Govt. informalliteracy

programme

>>Next Person

2 Private (Govt. grants)

3 Private (Not govt. grants)

4 NGO runinstitution

5 Madrasa (Govt. affiliated)

6 Madrasa (Kowmi)

2 No

PEC/equivalent

Class 6

>>Next Person

17 Engineering

6 NGO literacy course

7 Other(specify)

>>Next Person

>>Next Person

Class 7

18 Postgraduate/equivalent

19 Other(Specify)

JSC/equivalent

Class 9

SSC/equivalent

HSC/equivalent

01

02

03

04

05

06

07

08

09

10

11

12

13

14

15

2A

Page 5


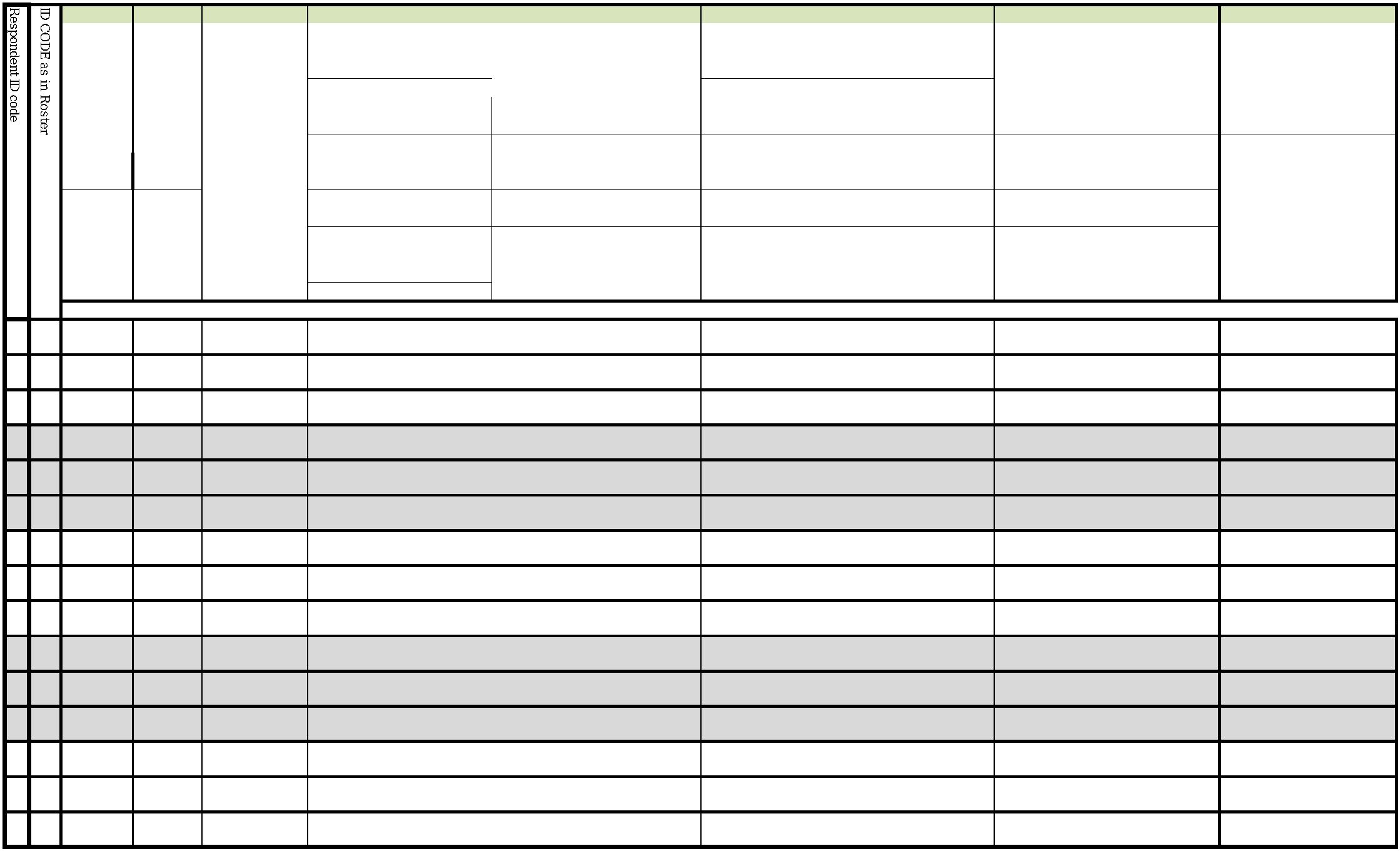


HIES 2016

SECTION 2: EDUCATION

PART B: CURRENT ENR OLLMENT (ALL PERSONS 5 YEARS AND OLDER)

ASK ONLY STUDENTS

1

2

3

4

5

6

7

Are you currently

attending school/

educational

Why aren't you attending

What class are you currently attending?

Are you receiving

any stipend for

education?

Which one?

How much did Do you

you receive in benefit

total in the past from the

school?

1 Do not want to study

more/completed studies

institution?

12 months?

tuition

waiver?

00 No class passed/pre-schooling

2 Too old to go back

01 Class 1

02 Class 2

03 Class 3

12 Vocational

3 No money/too expensive

13 Nursing

14 TechnicalEducation

15 Graduate/equivalent

16 Medical

1

2

3

4

5

6

PEC

JSC

SSC

HSC

1

2

Yes >>Q 3

No

4 No schools close to home 04 Class 4

05 PEC/equivalent

06 Class 6

1

2

Yes

1

2

Yes

No >>Q 7

No

5 Have to work

6 Attending family

chores

17 Engineering

07 Class 7

18 Postgraduate/equivalent

19 Other(Specify)

Graduate/equivalent

08 JSC/equivalent

09 Class 9

Post graduate/

equivalent

7 For marriage

>>Ne x t Person

10 SSC/equivalent

7

Other(Specify)

11 HSC/equivalent

TAKA

01

02

03

04

05

06

07

08

09

10

11

12

13

14

15

Continued……….

2B1

Page 6


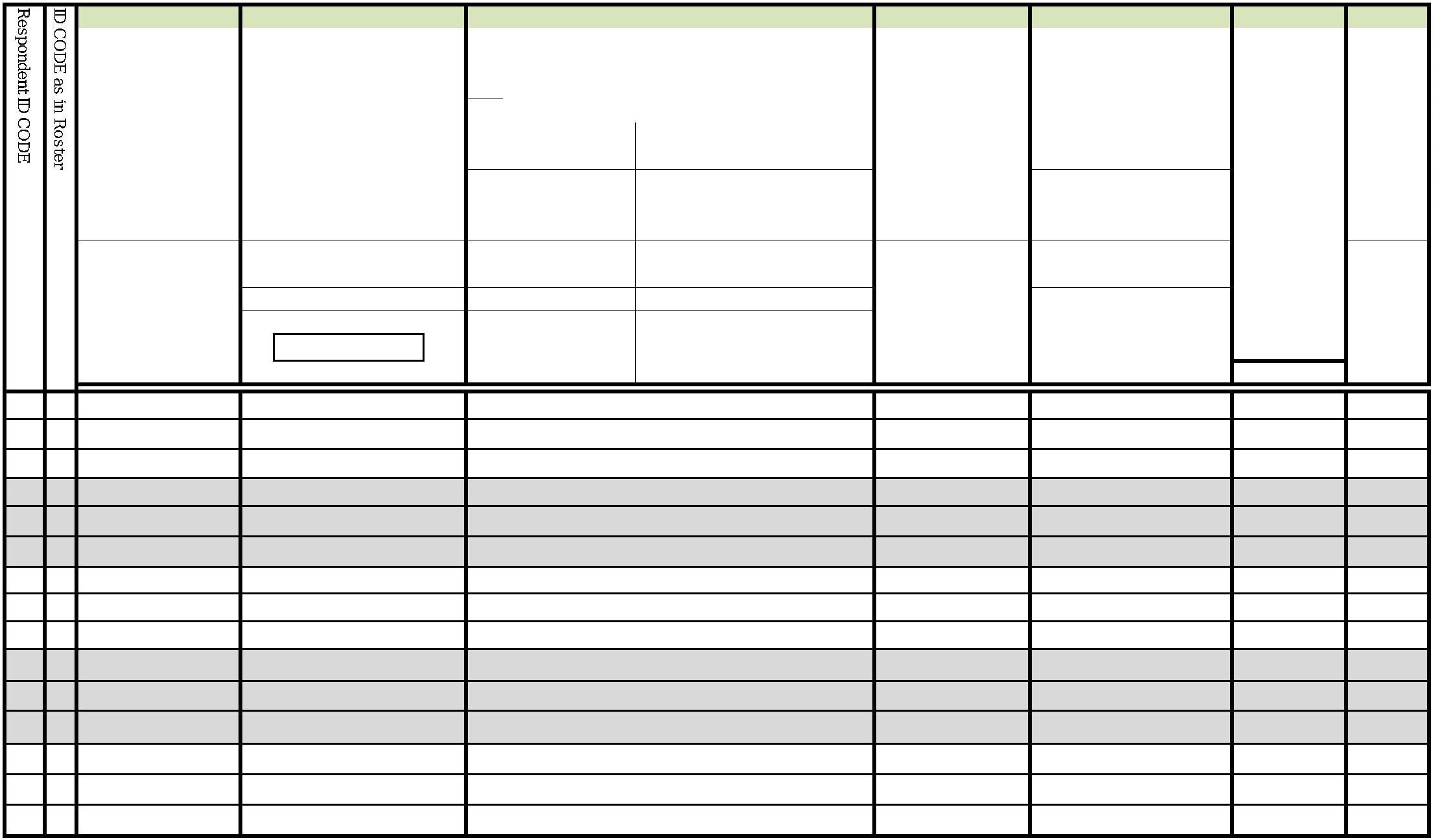

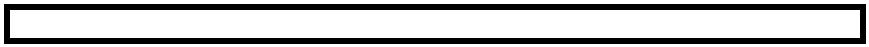


HIES 2016

SECTION 2: EDUCATION

PART B: CURRENT ENROLLMENT (ALL PERSONS 5 YEARS AND OLDER)

EXPENDITURE DURING THE PAST 12 MONTHS FOR EDUCATION

8

How much did your household spend during the past 12 months on your schooling?

WRITE THE EXPENSES IN TAKA

IF NOTHING WAS SPENT, WRITE "0" (ZERO) IF UNKNOWN, LEAVE BLANK

A

B

C

D

E

F

G

H

I

J

K

L

M

N

O

P

Q

Admission Annual

Institute Registration Examination Tuition Text books/ Exercise

Uniform Private Coaching Hostel expensed Transport Tiffin Internet/ e-

Total

expenses

Other edu-

related

expenses

fees

session

fees

fees/

fees

fees

fees Note books

books/

dress/

tutoring

fees

(including food)

cost

cost mail cost (edu-

related)

donation

stationary footwear

01

02

03

04

05

06

07

08

09

10

11

12

13

14

15

2B2

Page 7


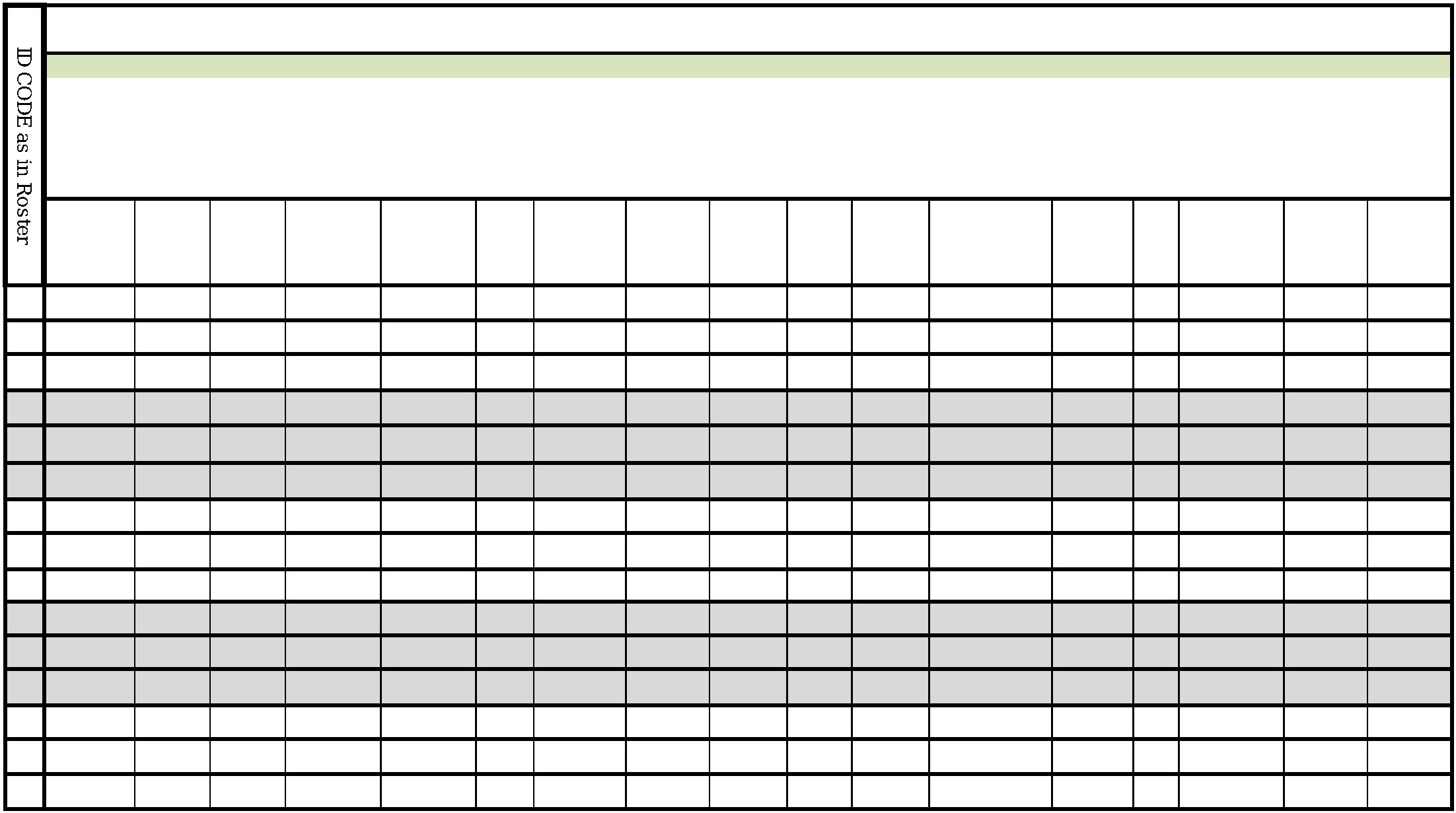


HIES 2016

SECTION 3: HEALTH

PART A: ILLNESSES AND INJ URIES (ALL HOUSEHOLD MEMBERS)

1

2

3

4

5

6

Have you suffered What chronic illness/ disability are you suffering from?

Have you suffered What Symptoms/Diseases did you suffer from?

Have you sought Why did you not seek any treatment?

from any chronic

illness/ disability in

the last 12

months?

from any

symptoms of

illness/injury in the

last 30 days?

any type of

Problem was not serious

1

Medical treatment

related to your

health problems

mentioned in

01 Chronic Fever

09 Skin problem

10 Diabetes

01 Diarrhoea

02 Fever

14 Malaria

02 Injuries / Disability

15 Jaundice

2

Treatment cost is too

much

03 Chronic Heart Disease 11 Cancer

03 Dysentery

04 Pain

16 Female diseases

17 Pregnancy related

18 Cancer

04 Respiratory Diseases/ 12 kidney Diseases

question 4(1)?

3

4

Distance is too long

Asthma/Bronchitis

Afraid of discovering serious

illness

13 Liver Diseases

05 Injury/Accident

05 Diarrhoea/Dysentery

06 Gastric/ ulcer

14 Mental Health

15 Paralysis

06 Blood pressure

07 Heart disease

19 Mental health

20 Paralysis

1 Yes

1 Yes

5

6

There was none to

accompany

2 No >>Q 3

07 Blood pressure

16 Ear/ENT problem

17 Eye problem

18 Other(specify)

2 No >>Q 15 08 Respiratory Diseases/

Asthma/Bronchitis

21 Epilepsy

08 Arthritis/ Rheumatism

22 Scabies/Skin diseases

23 Kidney Diseases

24 LiverDiseases

25 Ear/ ENT problems

26 Eye problem

1 Yes >>Q 7

2 No

Decision makerdoes not

think I should seek

treatment

09 Weakness

10 Dizziness

11 Pneumonia

12 Typhoid

7

8

Didn't know where to go

Other(specify)

13 Tuberculosis

27 Other(specify)

IN ORDER OF IMPORTANCE

IN ORDER OF IMPORTANCE

>>Q 15

1

2

1

2

3

01

02

03

04

05

06

07

08

09

10

11

12

13

14

15

Continued………………..

Page 8

3A1


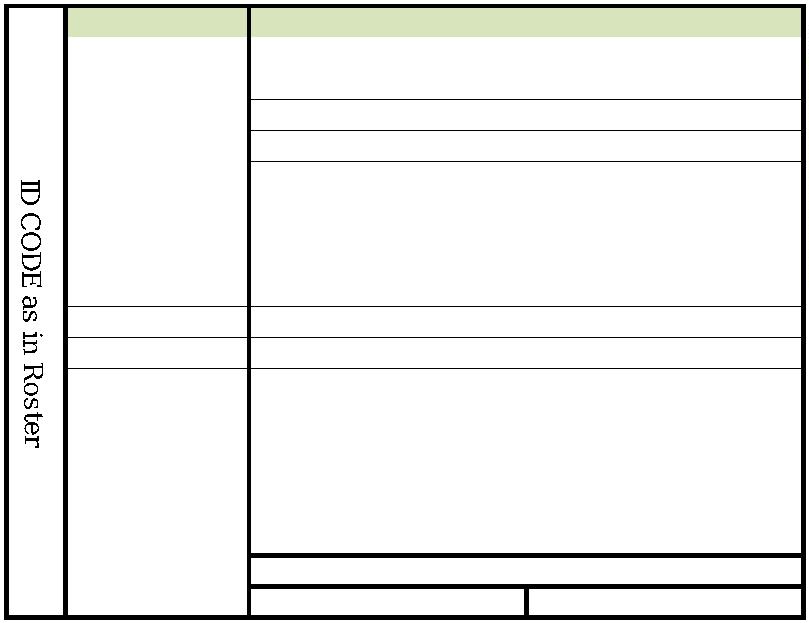

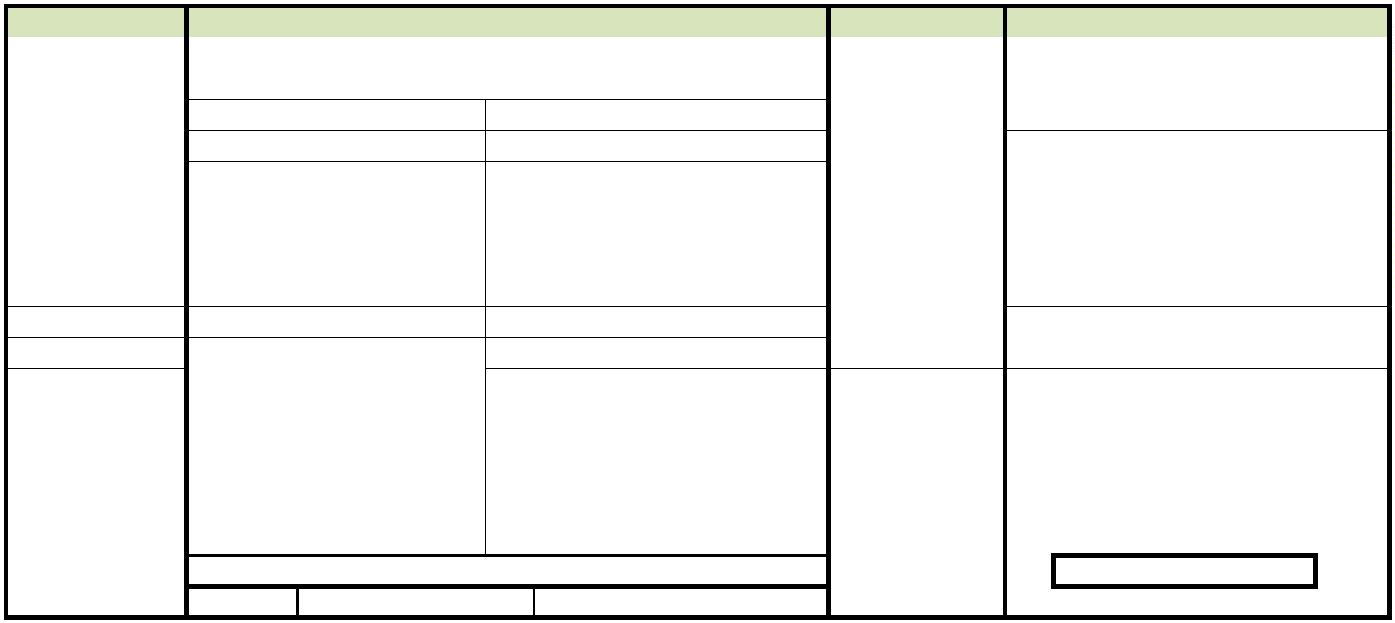

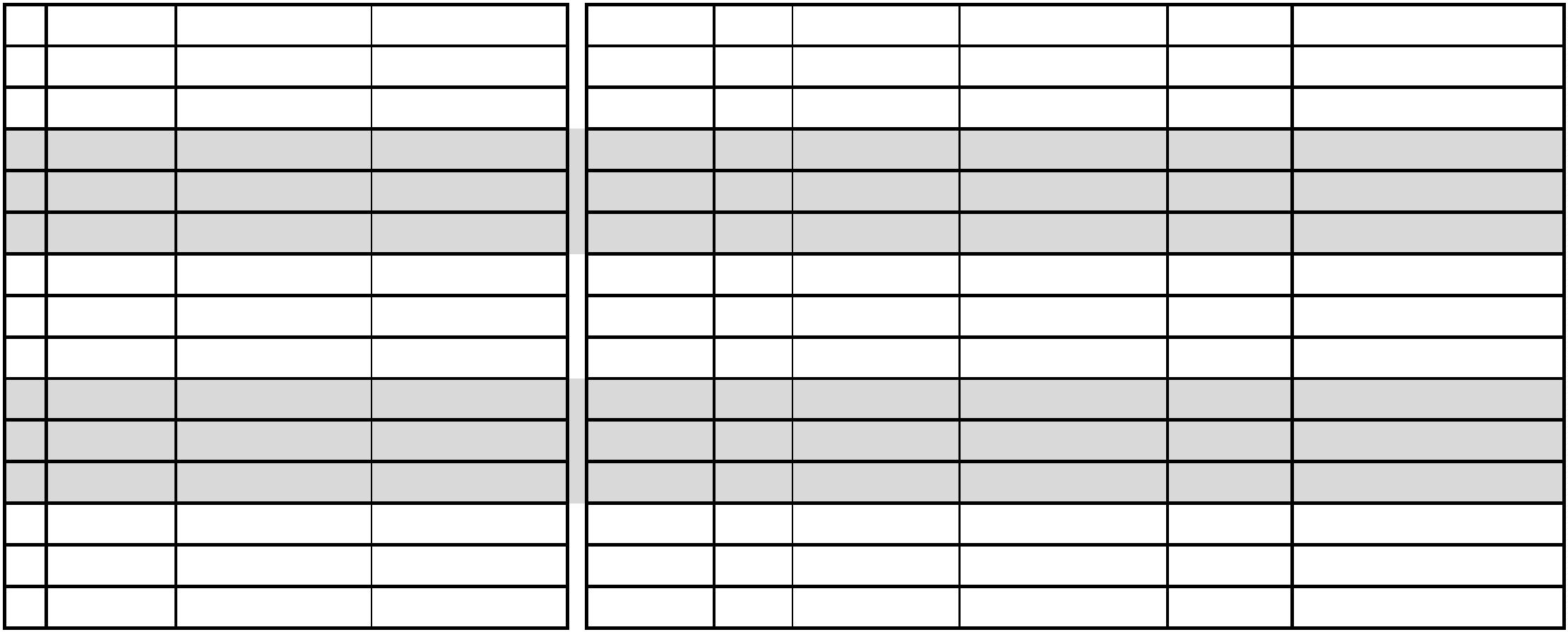


HIES 2016

SECTION 3: HEALTH

PART A: ILLNESSES AND INJ URIES (ALL HOUSEHOLD MEMBERS)

7

7B

8

9

10

Where did you get the

medicines from?

11

12

13

Which of the followingwere consultedforthis illness/injury(inthe order in which they

were consulted)?

Was the consulted

provider urban or rural?

How many days

aftersymptoms

began did you first

consultthis health

provider?

Why did you choose this provider?

How did you travel to the How much time

How long did you have

provider?

did it take to reach to wait at provider to be

the service

provider?

examined?

01 Govt. HealthWorker

10 NGO HealthWorker/Satellite Clinic

11 NGO Clinic/Hospitals

01 Private car

02 Taxi

02 Govt. Satellite Clinic/EPIOutreach

Center

01 Rural

02 Urban

01 Nearby

01 Govt. healthfacility

02 NGO healthfacility

03 Private healthfacility

04 Otherfacility, specify

05 Pharmacy/dispensary

06 Othershop

12 Govt. MedicalCollege/Specialized

Hospital

02 Acceptable cost

03 Bus

03 CommunityClinic

03 Availability of doctor

04 Availability of female doctor

05 Availability of equipment

06 Quality of treatment

07 Referred by other provider

04 Auto rickshaw

05 Rickshaw

06 Rickshawvan

07 Bullockcart

08 Countryboat

09 Engine boat

10 Ambulance

11 Walking

04 Union Health & Family Welfare

Centrer/UnionSubCenter

13 Private Clinic/Hospitals

14 Private MedicalCollege/Specialized

Hospital

05 Upazila HealthComplex

06 Maternal & Child Welfare Center

(MCWC)

15 QualifiedDoctor's Chamber

16 Non-qualifiedDoctor's Chamber

17 Pharmacy/Dispensary

18 Homeopath

07 Notavailable

IF IMMEDIATELY, 08 Referred by relatives/ friends

WRITE "00"

08 Could not afford

09 Other(specify)

07 Govt. District/Sadar/GeneralHospital

REFER TO THE FIRST

CONSULTEDPROVIDER

IN Q. 7

09 Reputation

08 Govt. MedicalCollege/Specialized

Hospital

10 Other(specify)

19 Ayurbed/Kabiraj/Hekim

20 OtherTraditional/Spiritual

21 Family/Self Treatment

22 Other(Specify)

12 Callingdoctorat

home

09 OtherGovt. Specify

1st

13 Other(specify)

2nd

No. of days

Code

Code

Code

HOUR MINUTES

HOUR

MINUTES

01

02

03

04

05

06

07

08

09

10

11

12

13

14

15

Continued……………

Page 9

3A2


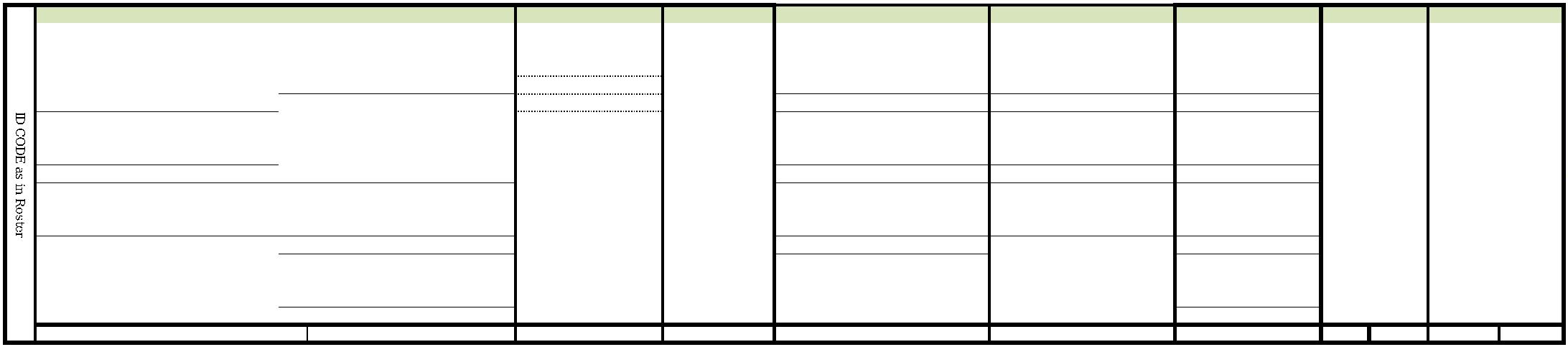

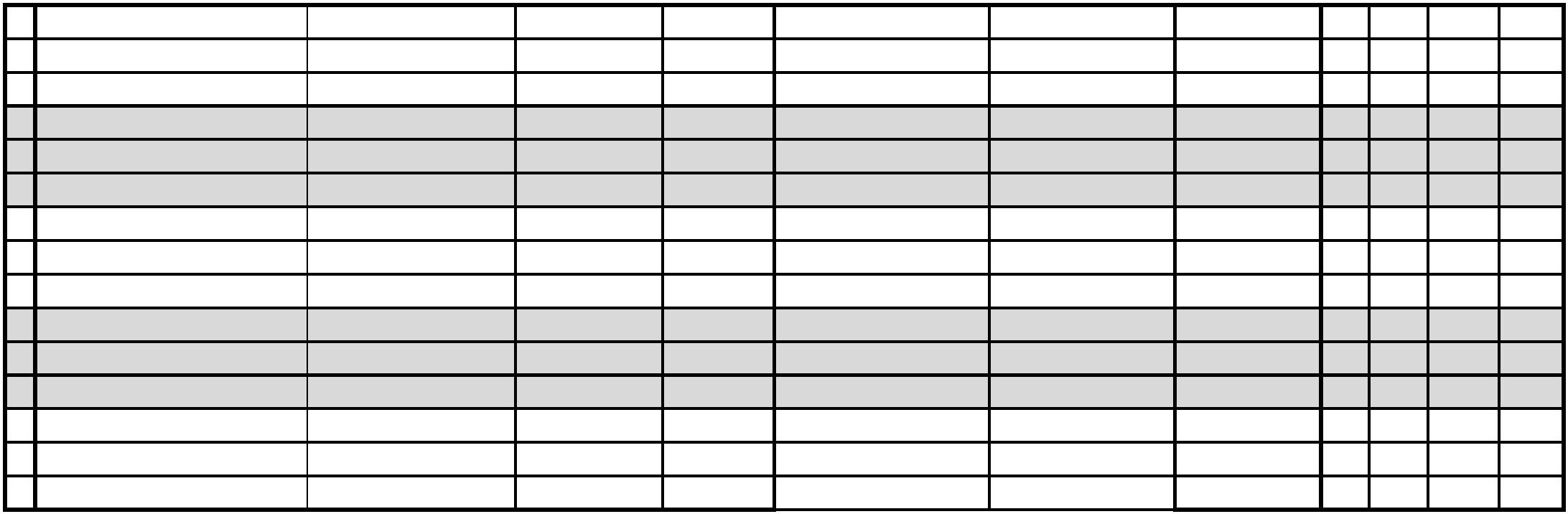


HIES 2016

SECTION 3: HEALTH

PART A: ILLNESSES AND INJ URIES (ALL HOUSEHOLD MEMBERS)

14

15

16

17

What was the total cost of outpatient treatment during the past 30 days?

How much did you spent in the following during the past 30 days?

Where you admitted to hospitaland stayed If yes, for how many

overnightduring the last 12 months?

nights did you stay at

the hospital?

WRITE THE VALUE IN TAKA

WRITE THE VALUE IN TAKA

IF NOTHING WAS SPENT, WRITE "0" (ZERO) IF UNKNOWN, LEAVE

BLANK

IF NOTHING WAS SPENT, WRITE "0" (ZERO) IF UNKNOWN, LEAVE BLANK

1

2

3

Yes

No

>>Next person

>>Next person

Don'tknow

A

B

C

D

E

A

B

C

D

E

Consultation

fees (visit)

Costof

Medicines

Cost of Test/

Investigation

Transport Totaloutpatient Immunization

cost cost

Contraceptives

ORS

Routine

medicines for

chronic illness

Routine

medical

check up

Code

Days

01

02

03

04

05

06

07

08

09

10

11

12

13

14

15

Continued………………..

Page 10

3A3


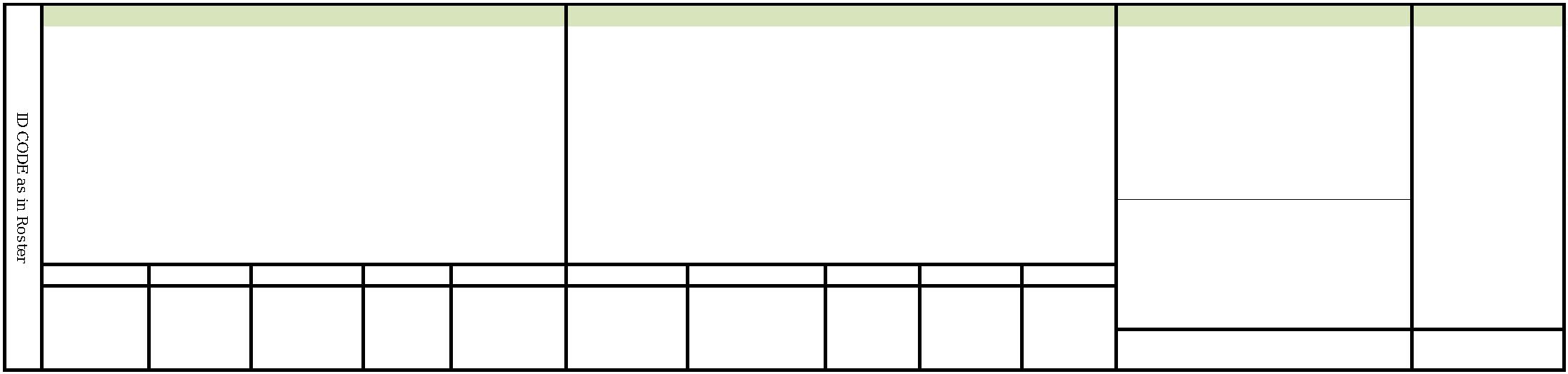

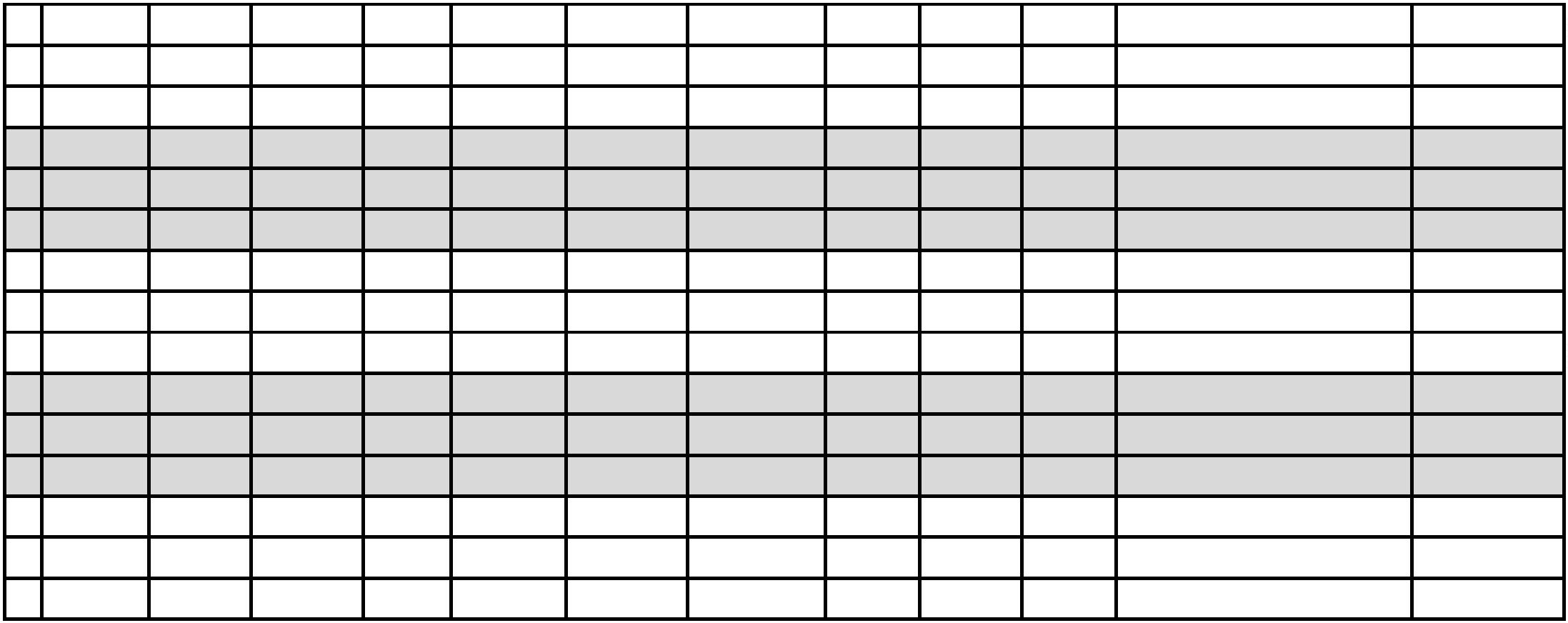


HIES 2016

SECTION 3: HEALTH

PART A: ILLNESSES AND INJ URIES (ALL HOUSEHOLD MEMBERS)

19

18

20

21

Reasons (symptom/disease/condition) for

Which hospitalyou were admitted in?

What was the total cost of inpatienttreatmentduring the past12 months?

How did you finance the costof

hospitalization?

outpatient/inpatient treatment?

01 Diarrhoea/Dysentry

02 Fever

14 Pregnancy related

01 Regularincome

15 Cancer

01 Govt. Upazila Health Complex

02 Household saving

03 Pain

16 Mentalhealth

17 Paralysis

02 Govt. Maternal&Child Welfare

Center(MCWC)

WRITE THE VALUE INTAKA

IF NOTHING WAS SPENT, WRITE "0" (ZERO) IF UNKNOWN, LEAVE BLANK

03 Sold personalbelonging

04 Sold Livestock

04 Injury/Accident

05 Blood pressure

06 Heartdisease

18 Skin diseases

19 Kidney Diseases

20 LiverDiseases

03 Gov district/Sadar/Generalhospital

04 Govtmedicalcollege hospital

05 Govtspecialized hospital

05 Sold Agriculturalproduct/Tree

06 Sold permanentassets

07 Mortgage of Assets/Land

08 Borrowed fromFriends/

Relatives/Office

07 Respiratory Diseases/

Asthma/Bronchitis

21 Ear/ ENT problems 06 OtherGovthospital(specify)

08 Weakness/Dizziness

09 Pneumonia

22 Eye problem

07 NGO generalhospital

23 Other(specify)

08 NGO Medicalcollege hospital

09 NGO specialized hospital

09 Borrowed from Money Lender

10 Assistance fromfriends &relatives

10 Tuberculosis

A

B

C

D

E

F

G

H

I

J

11 Malaria

12 Jaundice

10 Private generalhospital/clinic

11 Private medicalcollege hospital

Operation Consultation/ Bed/cabin Costof

alcost Doctorfees charges medicines investigations cost

Costof

Transport Informal Other

Maternity cost

Total

inpatient

Cost

11 Other(specify)

Tips

formal

(Mention three answers in order of

13 Female diseases

12 Private specialized hospital

charges

1

2

3

rank)

13 Otherprivate hospital(specify)

Clinic Midwife Others

1

2

3

01

02

03

04

05

06

07

08

09

10

11

12

13

14

15

3A4

Page 11


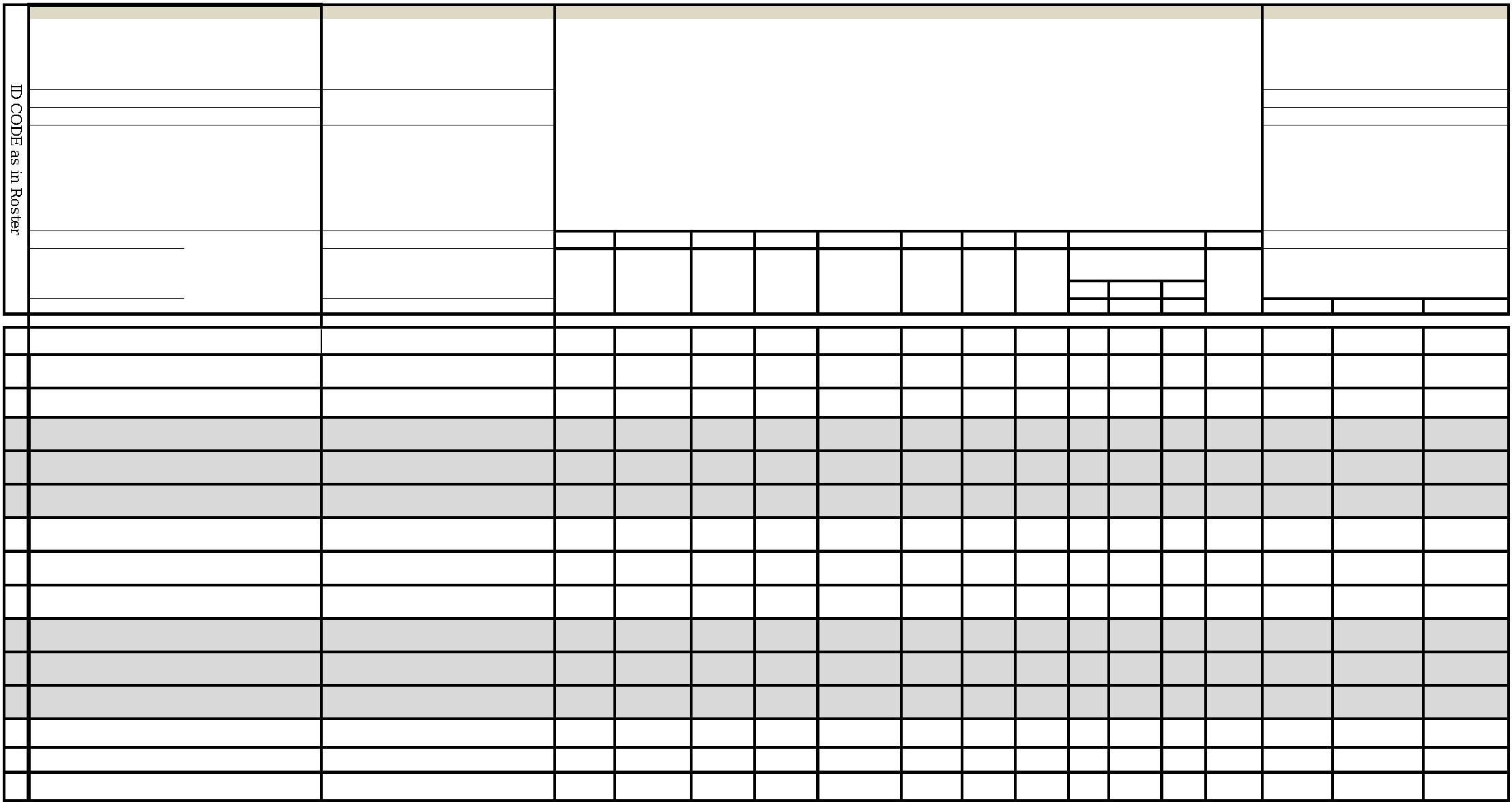

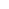


HIES 2016

SECTION 4: ECONOMIC ACTIVITIES AND WAGE EMP LOY MENT

PART A: ACTIVITIES (ALL PERSONS 5 YEARS AND OLDER)

A

C

T

I

V

I

1

2

3

4

5

6

7

8

Whateconomic activities did (name) do in the past 12

How many

months did

you do this

activity in the

last 12

Onaverage, how On

many days per

month?

Where did you do this activity? What was major If you were engaged in agricultural If you were engaged in non agriculture

months? Beside this,

average

how many

hours per

day?

field of economic sector, what was youremployment sector, what was your work status?

activities you

engaged in?

status?

What otheractivities did you do?

Activities: Service/Wage employment/Allactivities(agric/non-

agric) conducted under self or joint ownerships.

In which area?

months?

T

Y

1

2

Rural

1 Agriculture

1 Day labourer >>Part- B

1

2

3

4

Day labourer

>>Part- B

Urban

2 Non

Agriculture

2 Self employed >>Section-7

Self employed >>Section-5

S

E

R

I

A

L

WRITE DISTRICT CODE

FROMCODE LIST

3 Employer

>>Section-7

>>Part- B

Employer

Employee

>>Section-5

>>Part-B

>>Q 08 4 Employee

CODE

DESCRIPTIONOFACTIVITY OCCUPATIO INDUSTRY

DAYS/ MONTH HOURS/

DAY

N CODE

CODE

MONTH

R/U

DISTRICT CODE

CODE

CODE

A

B

C

D

E

F

G

H

I

J

K

L

M

N

O

Page 12

4A


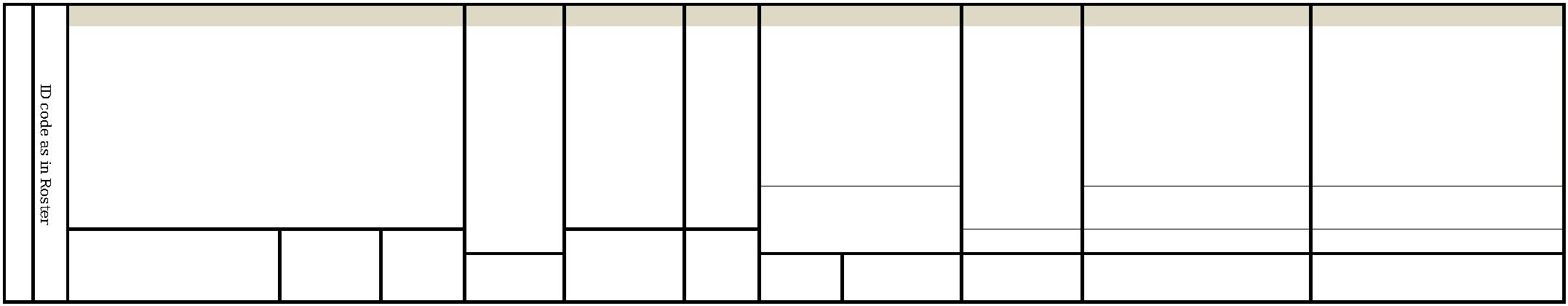

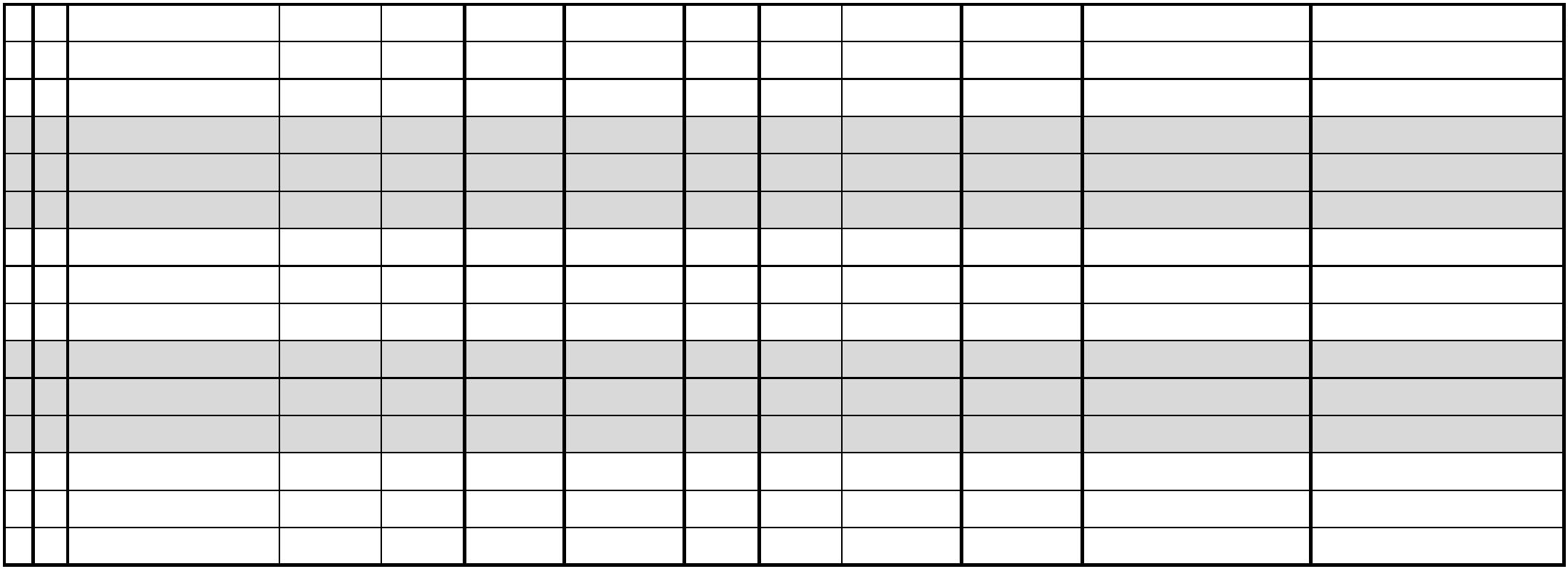


HIES 2016

SECTION 4: ECONOMIC ACTIVITIES AND WAGE EMP LOY MENT

PART B: WAGE EMPLOYMENT

DAILY WAGE

SALARIED WAGE

C

T

I

V

I

1

2

3

4

5

6

7

8

9

Were you paid on

a daily basis?

What was the daily wage in cash in Did you receive payments in-

the past 12 months?

What type of in-

kind paymentdid

you receive?

How much did you

receive perday?

What type of org. do/ did you What is your

work for? gross

remuneration per remuneration afterall overthe past 12 months (tips,

What is your total net What is the total value of in-kind

take-home monthly orotherbenefits you received

kind?

1

2

3

4

5

6

7

8

9

Govt. organization

month?

deduction atsource? bonuses ortransport) for this job?

Autonomous body

Private office/institution

Public mill/ factory

Private mill/ factory

Localgovt

T

Y

1

2

Yes

No

1 Paddy

2 Rice

WRITE THE

QUANTITY IN KG AND

THE TOTAL VALUE IN

TAKA

1

2

Yes

No

>>Q 6

>>Next Activity/Next person 3 Wheat

4 Meal

S

E

R

I

>> Next Activity/Next Person

5 Other(specify)

NGO

Highest

1

Lowest

2

Average

3

QUANTITY

KG

VALUE

TAKA

Household

A

CODE

CODE

CODE

Other(specify)

TAKA

TAKA

TAKA

A

B

C

D

E

F

G

H

I

J

K

L

M

N

O

Page 13

4B


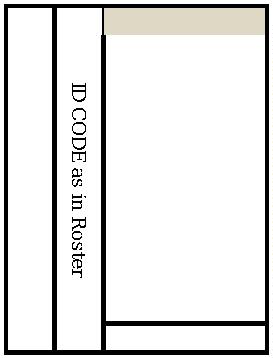

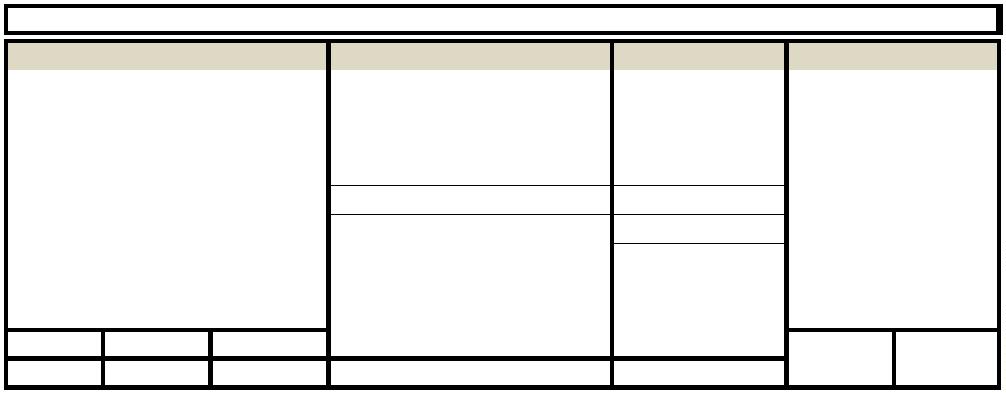

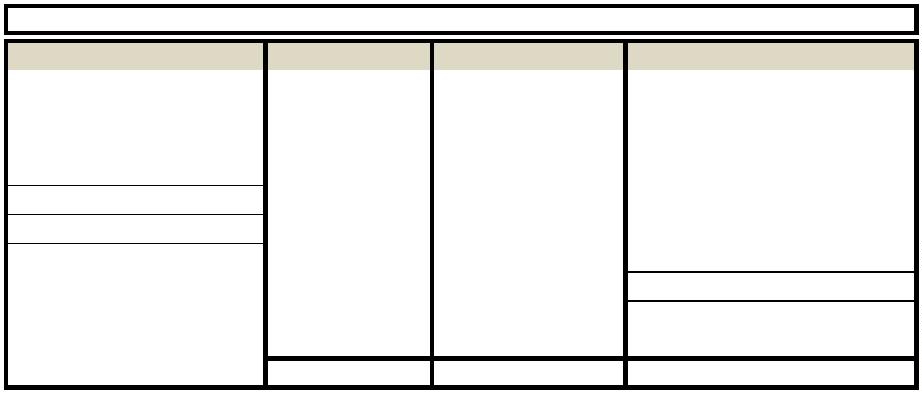

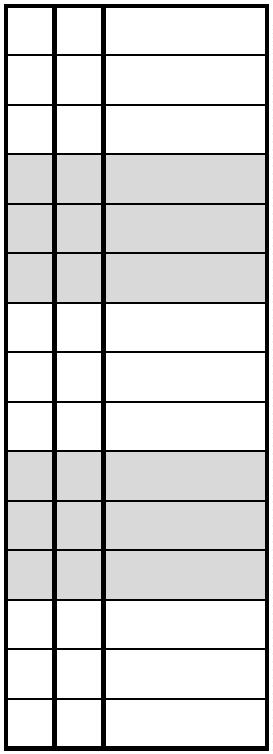

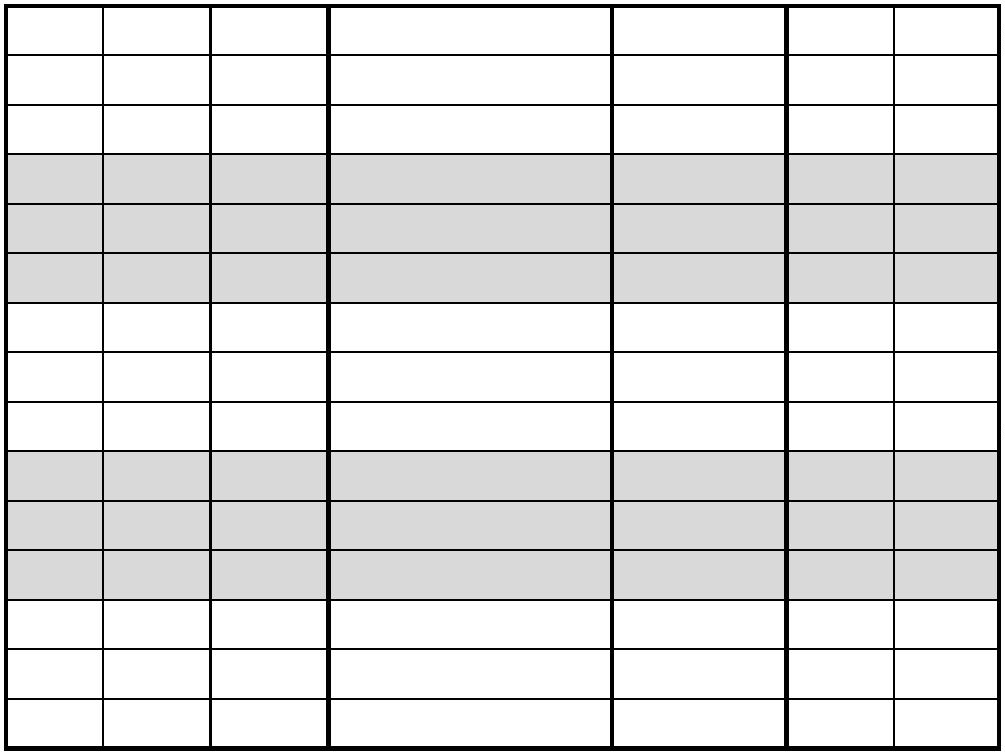

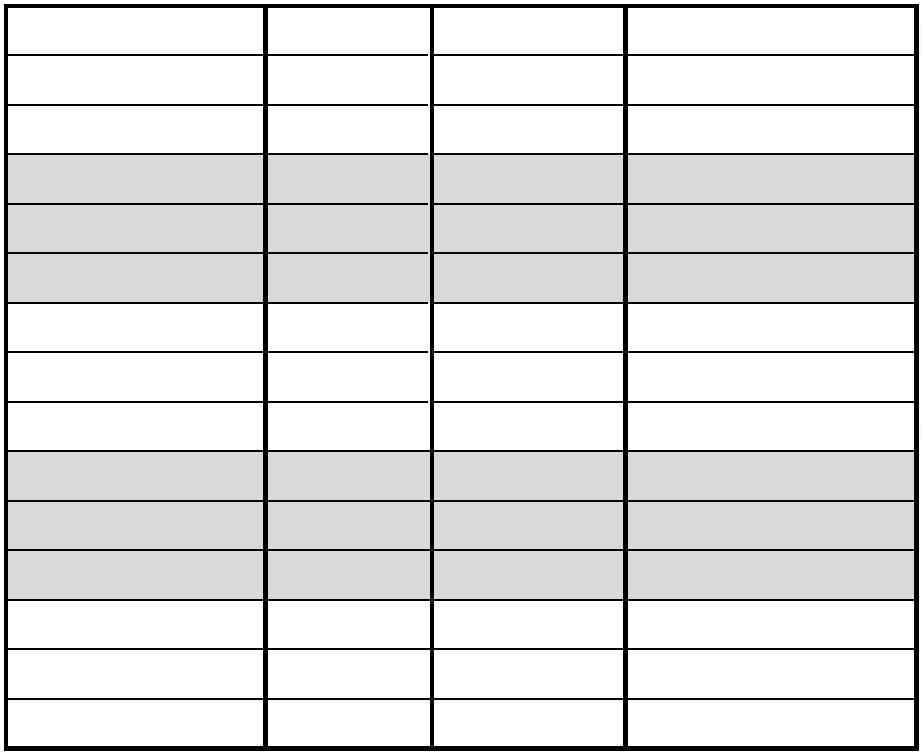


HIES 2016

SECTION 5: NON-AGRICULTURAL ENTERPRISES

E

1

2

3

4

5

6

7

8

9

10

N What kind of enterprise did/ do yourhousehold solely or Which people in the

For how long has Where do you operate the How many

What is the

household's

Whatshare of

profit is owned customers?

by household?

Who are your

Is the enterprise/ What was yourmain source of

activity registered finance forsetting up the

by the govt. or

localgovt.?

jointly own and operate in the past 12 months?

household work in this

enterprise/activity?

this enterprise/

activity been

operating?

enterprise/activity?

months did

the enterprise share of this

/activity enterprise/

operate in the activity?

past 12

months?

T

E

R

P

R

I

business?

1 Households/

Individuals

01

Inherited/ through gift

1

2

3

4

Own house

2 Govt. or other public

firm

02 Own savings

CROSS CHECK ACTIVITIES

WRITE ID CODE FROM

ROSTER

Rented house

Govtland/house

03 Borrowing from relatives/

friends

REPORTED IN SECTION 4 PART A

3 Private enterprises

1

2

3

Yes

No

S

E

Fixed location outside

house

04 Agriculturaldev. bank

05 Commercialbank

06 Grameen bank

4 Foreign individual

/organization

Not

applicable

WRITE DESCRIPTIONINFULL

5

Variable location

N

U

M

B

E

R

5 Others (specify)

07 OtherfinancialInst.

08 NGO/ Reliefagency

09 Sale of assets

WRITE IN

ACCORDING TO

IMPORTANCE

10 Supplier's credit

11 Other(specify)

INDUSTRY

CODE

ID CODE

DESCRIPTIONOF ACTIVTITY

1

2

3

4

5

YEAR MONTH

CODE

MONTHS

PERCENTAGE PERCENTAGE

1st

2nd

CODE

1st

2nd

1

2

3

4

5

Continued…………..

Page 14

5A


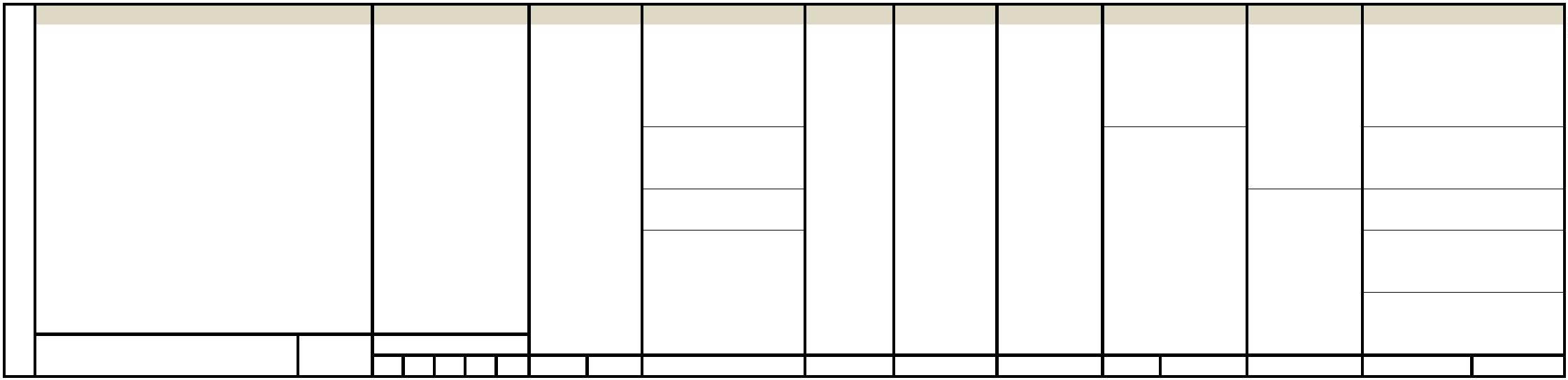

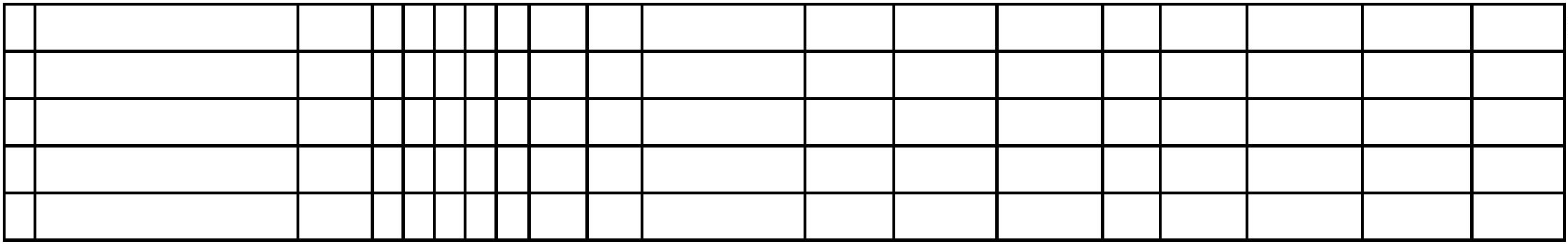


HIES 2016

SECTION 5: NON-AGRICULTURAL ENTERPRISES

INCOME FROM THE ENTERPRISE [WRITE THE VALUE IN TAKA]

16 17 18 19 20

11

How many

employees

were engaged months?

in the past 12 01 No problem

months?

12

13

Gross

revenues

over the

past 12

months

14

15

21

22

23

E

N

T

E

R

P

R

I

Whatproblems, if any, have you had in

running yourbusiness in the last 12

Expenditures on Expenditure on Expenditure on Expenditure on Expenditure on Other

Net

Expenditure on Sales of

capitalgoods in assets in

past 12 months past 12

months

If someone wanted

to buy this

enterprise today,

how much would he

have to pay?

wages in the last rent in the past raw materials Fuel,

finished goods operating

revenues

12 months

12 months

in the past 12 Kerosene,

purchased for expenses in

months

Electricity, etc. reselling in the the past 12

in the past 12 past 12 months months

months

02 Inadequate capital or credit

03 Inadequate tech. knowledge

04 Lack of required no. of expertise

05 High-cost of running ent.

06 Watersupply problem

07 Powersupply problem

08 Problems with equipment/ spare

parts

09 Governmentregulations

10 Lack of raw materials

11 Lack of customers

BOTHCASH

AND IN KIND

S

E

N

U

M

B

12 Transportproblems

13 Telecom and Internetaccess

E

R

14 Other(specify)

NUMBER

1

2

TAKA

TAKA

TAKA

TAKA

TAKA

TAKA

TAKA

TAKA

TAKA

TAKA

TAKA

1

2

3

4

5

Page 15

5B


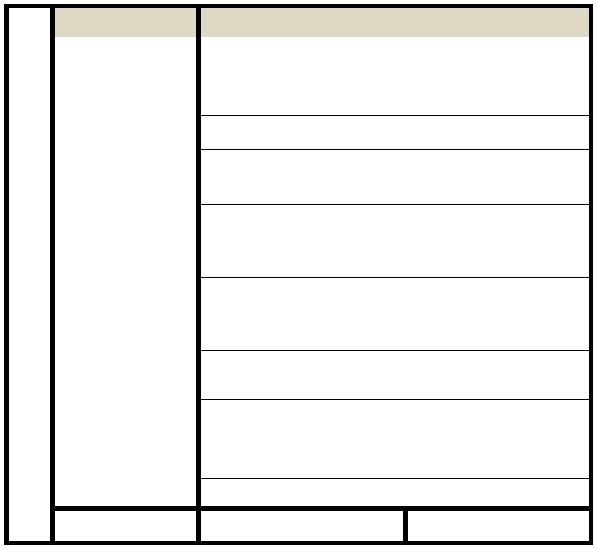

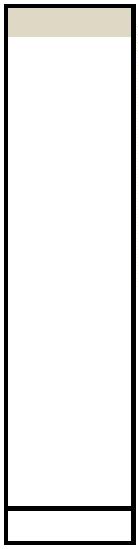

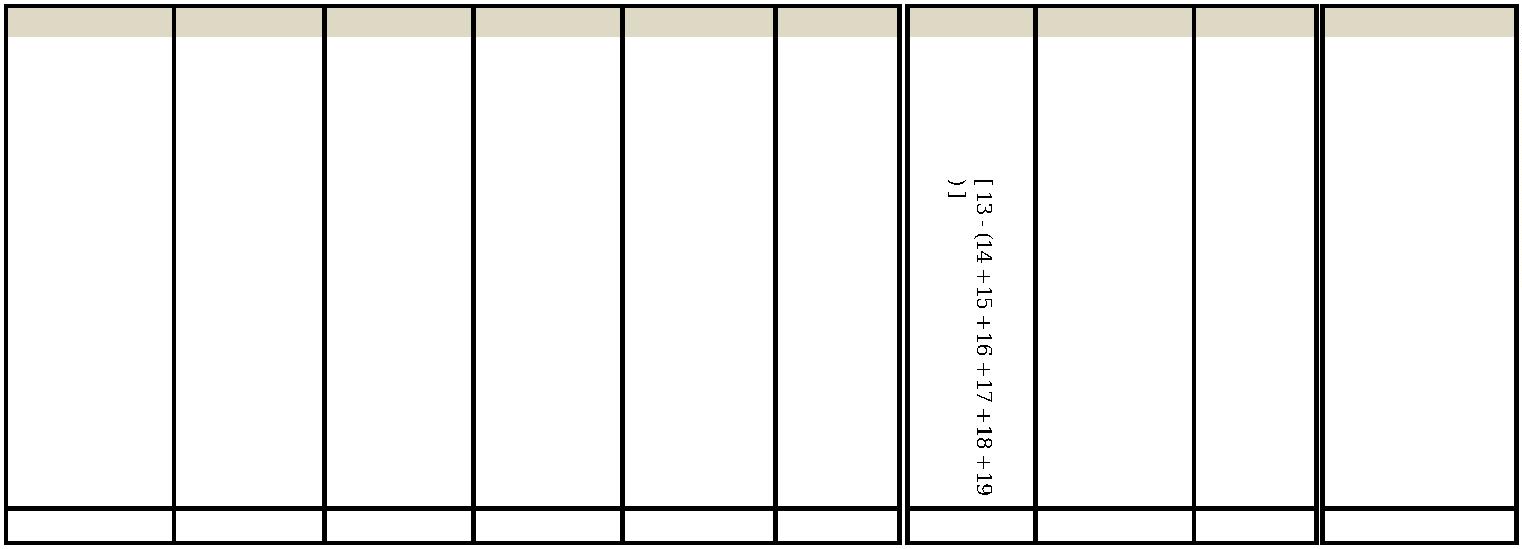

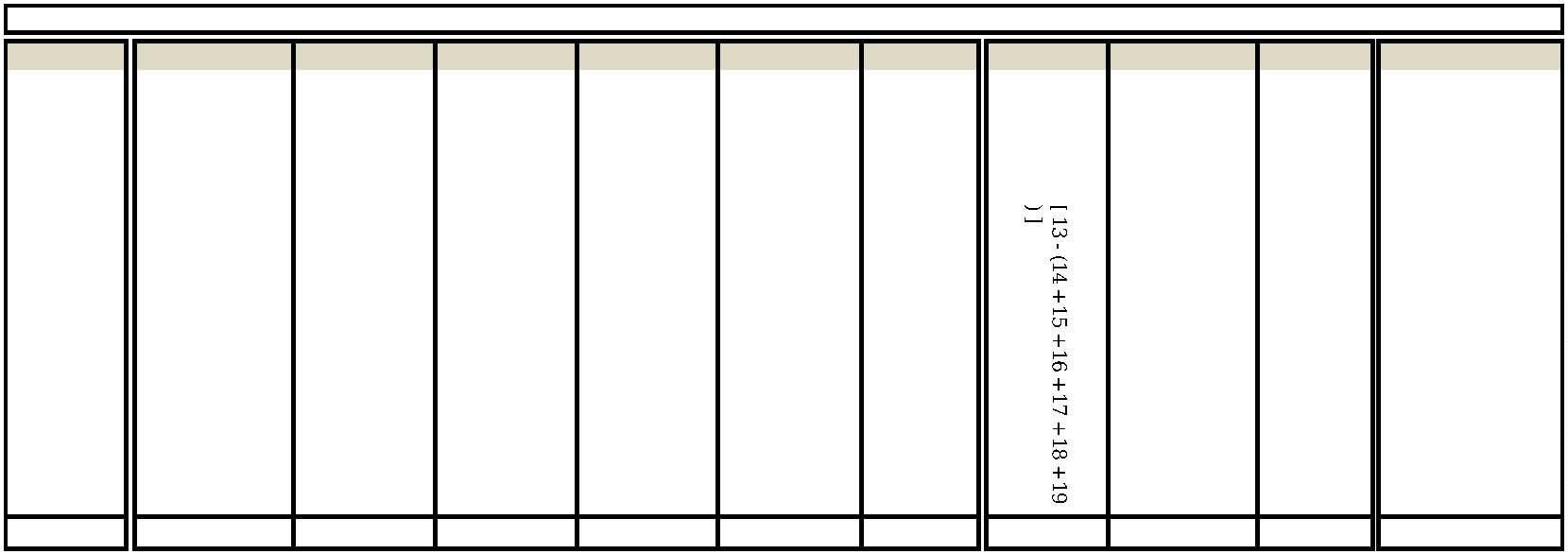

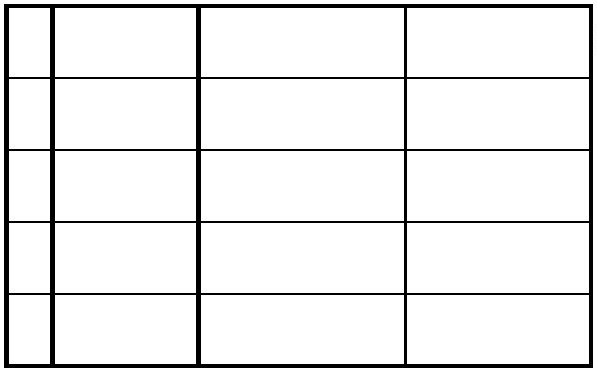

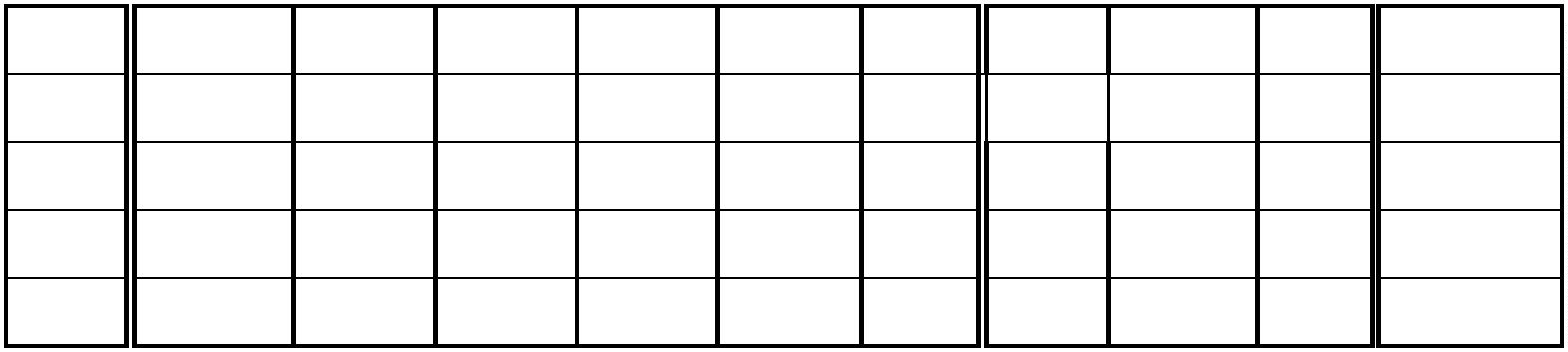

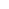


SECTION 6: HO US ING

PART A: HOUSING INFORMATION

RESPONDENT SHOULD BE HOUSEHOLD HEAD OR OTHER RESPONSIBLE HOUSEHOLD MEMB ER

HIES 2016

Has yourTubewell been tested for arsenic?

1 ID code of the respondent

13

1 Yes

2 No

>>Q 16

2 How many rooms does yourhousehold occupy?

(Excluding rooms forbusiness)

Number of rooms:

14 Was arsenic found?

1 Yes

2 No

>>Q 16

3 Does your dwelling posses a separate dining room?

1

Yes

2

No

15 If Yes, what is the alternative source of drinking water?

1

2

3

Supply

Contaminated Tubewell 5

Non contaminated

tubewell

4

Pond/river

Well

Waterfall string

Other(specify)

4 Does your dwelling posses a separate kitchen?

Yes

1

2

No >>Q 6

6

7

5 If Yes, does thedwelling share this facility with others who are not members

of this household?

1

Yes

2 No

16 What is the main source of water for other use?

6 What type of stove do you have?

1

2

3

Supply water

Tubewell

Pond/river

4

5

6

Well

Waterfall/string

Other(specify)

1

2

3

4

Electric

5 Concrete stove purchased /received

from NGO

Gas

Own built traditional mud

Improved stove (mud stove

purchased/received from

6 Pre-fabricated steel stoves (non-electric

&non-gas)

17 Does the household have an electricity connection?

1 Yes 2 No

7 None

>>Q 19

7 What is the construction material of the walls of the main room?

hrs.

1

Straw/Bamboo/

4 Wood

Polythene/Plastic/ Canvas

5 Brick/Cement

6 Other(specify)

19 Does the household have a landline telephone connection?

1 Yes 2 No

2

3

Mud/Unburnt brick

Tin (CI sheet)

20 Does yourhousehold own a computer/Laptop/Notebook/Tablet?

1 Yes 2 No

8 What is the construction material of the roof of the main room?

1

Straw/Bamboo/

3 Tally

Polythene/Plastic/ Canvas

4 Brick/Cement

5 Other(specify)

21 Does this household have access to internet/e-mail facilities?

1 Yes 2 No >>Q 23

2

Tin (CI sheet)

9 What is the total usable space/area of covered rooms?

SQ FT

22 How does yourhousehold's members most often access the internet?

1 Mobile phone

4 Community information Center

5 Other(specify)

10 What kind of toilet facility do members of yourhousehold usually use?

2 Home computer

1

2

3

Sanitary

Pacca latrine (water seal)

Pacca latrine (pit)

4 Kacha latrine (perm)

5 Kacha latrine (temp)

6 Open space/no latrine

3 Cybercafe

23 What is your present occupancy status?

1 Own

11 Does the household share this toilet facility with otherhouseholds?

Yes 2 No

12 What is the main source of drinking water?

2 Rented

3 Rent-free

1

24 If you want to buy or construct a dwelling just like this

TAKA

today, how much money would you have to pay?

1

2

3

Supply

Tubewell

Pond/river

4 Well

5 Waterfall/string

6 Other(specify)

25 Is this a slum household?

1 Yes

2 No

If source of drinking water is other than 2 (Tubewell) >>Q 16

6A

Page 16


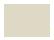


HIES 2016

6A

Page 16

SECTION 6: HO US ING

HIES 2016

PART B: SHOCKS AND COPING

1

2

3

4

5

6

Description of distress events

Did you experience

When did the shock How long did As a result of the [SHOCK], was there a decline in

How did your household

[shock] during the

past 12 months?

first occur?

the shock

last? (If

Shocks

continued till

now write

"99")

yourhouseholds?

cope with this [shock]?

Up to three answers

with rank for each shock

experienced, use code

below:

1-J an

2-Feb

3- Mar

4-Apr

7-J ul

Code

8-Aug

9-Sep

10-Oct

1 Yes >>Q 3

Income

Assets

Food

Production

Food

purchased

2 No >> Next

shock

5- May

6-J un

11-Nov

12-Dec

NUMBER OF 1 Yes

DAYS

1 Yes

2 No

1 Yes

2 No

1 Yes

2 No

1st

2nd

3rd

CODE CODE CODE

2 No

101 Drought / Irregular rains

102 Floods

103 Landslides/ Erosion

104 Unusually High Level of Crop Pests and Disease

105 Unusually High Level of Livestock Disease

106 Unusually High Prices of Agricultural Inputs

107 Unusually Low Prices of Agricultural Output

108 Reduction in the Earnings of currently (Off-Farm)

Employed Household Member(s)

109 Loss of Employment of Previously Employed

Household member(s) (not due to illness/accident)

111 Serious Illness or Accident of income Earner(s)

112 Serious Illness or Accident of other HH Member(s)

113 Death of Income Earner(s)

114 Death of other HH Member(s)

115 Theft of Money/Valuables/Non Agricultural Assets

116 Theft of Agricultural Assets/Output (Crop or Livestock)

117 Conflict/Violence

118 Fire/Tornedo/Earthquake, etc

119 Other(Specify)

CODES FOR Q6- COPING STRATEGIES

01 Unconditional help provided by relatives/friends

09 Obtained credit

02 Unconditional help provided by local government

03 Change dietary patterns involuntarily

10 Sold durable household assets

11 Sold land or building

04 Change cropping practices (crop choices ortechnology)

12 Rented out land/building

13 Distress sales of animal stock

05 Household member(s) took on more non-farm (wage or self employment)

06 Household members took on more farm ways employment

07 Household members migrated

14 Send children to live elsewhere

15 Reduce expenditure on health and education

16 Other(specify)

08 Relied on savings

6B

Page 17

HIES 2016

SECTION 7: AGRICULTURE

PART A: LANDHOLDING

WRITE '0' IF NONE.

Acres

Decimal

1 Total cultivable agricultural land owned:

Acres

Decimal

2 Totaldwelling-house/Homestead land owned:

Acres

Acres

Decimal

Decimal

3 Total Non-cultivated Land

4 Total cultivable agricultural land rented/ share-cropped/mortgaged in:

Acres

Acres

Decimal

Decimal

5 Total cultivable agricultural land rented/ share-cropped/mortgaged out:

6 Total operating land (1+2+3+4- 5)

7A

Page 18

HIES 2016

SECTION 7: AGRICULTURE

PART B: CROP PRODUCTION

1

2

3

4

5

6

7

8

Did you or anyone in your household cultivate Did you cultivate

How much land

How much in total of crop How much did your How much did your How much did your How much of it was used for the following purposes in the last 12 months?

household sell in the household stock in

last 12 months? the last 12 months?

any following crops in the last 12 months?

(crop) in the last 12 did you cultivate did you produce in the last household

months?

under this crop in 12 months?

the last 12

months?

consumed in the

last 12 months?

WRITE TOTAL QUANTITY USED (IN KG)

4=5+6+7+8

1

2

Yes

1

2

Yes

No

>>Ne x t Crop

PRODUCED (IN KG) AND

THE UNIT PRICE (INTK)

A

B

C

D

E

Waste

F

Other

G

Total

No >>page 20

Given to Given for Used as

landlord

Feed for

animals

Wages

Seed

Crop/by product

Code

Code

Acres Decimal

kg

Taka/kg

kg

kg

kg

kg

kg

kg

kg

kg

kg

kg

Aus

01

Aman

02

Boro

03

04

05

06

07

Wheat

Maize

J ute

Sugarcane

Pulses

08

09

Oil Seed

By Product

By product of paddy

By product of wheat

11

12

By product of jute

13

14

By product of sugarcane

Continued…………

Page 19

7B1

SECTION 7: AGRICULTURE

PART B: CROP PRODUCTION

HIES 2016

Other Crops: Write the name of the other crops Cultivated by the household

1

2

3

4

5

6

7

8

Did you or any one in your

household membercultivate any

crop in the last 12 months?

Did you

How much land How much in

How much

How much

did your

household

How much

did your

household

stock in the

last 12

How much of it was used for the following purposes in the last 12

months? (in kg)

cultivate (crop) did you cultivate total of crop did did your

in the last 12

months?

under this crop in you produce in household

the last 12

months?

the last 12

months?

consume in sell in the

the last 12

months?

last 12

months?

4=5+6+7+8

months?

1 Yes

1 Yes

2 No >> Part C: Non-Crop

2 No

A

B

C

D

E

F

G

Activites

>>Next crop

given in given

landlord for

wages

Used as Feed for Waste Other Total

seed animals

Vegetable

Potato

Code

Code

Acres Decimal Kg

Taka/kg

Kg

Kg

Kg

15

16

17

18

19

21

22

23

Onion

Garlic

Ginger

Turmeric

Tomato

Brinjal

Green Banana/Green

Papaya

Cauliflower/Cabbage

Pumpkin

24

25

26

27

28

29

31

32

33

Radish

Green Chili

Bean

Patal

Lady's Finger

Puisak

Others (specify)

Fruits

Mango

41

42

43

44

45

46

47

48

49

J ackfruit

Ripe Banana

Ripe Papaya

Pineapple

Leechee

Melon/Bangi

Guava

Others (specify)

Page 20

7B2

HIES 2016

SECTION 7:

PART C: NON-CROP ACTIVITIES (Livestock and poultry)

1

Did you or anyone in your household raise any livestock or poultry birds in the last 12 months?

1

2

Yes

No >>Q9

LIVESTOCK AND POULTRY (Do you or any member of yourhousehold)

LIVESTOCK PRODUCTS

2

3

4

5

6

7

8

Name of domestic animals

How many ..[NAME How many were born/did

How many died/did your

How many did your

householdconsume in the

12 months?

Animal production and by-

product

How much did you How much did you

produce in the last sell in the last 12

How much did you

consume in the last 12

months?

OF THE ANIMAL]..

own? What is their

total value?

your household purchase in household sell in the last 12

the last 12 months?

months?

12 months?

months?

WRITE TOTAL NO. AND

THE TOTAL VALUE IN

TAKA

WRITE TOTAL NO. AND THE WRITE TOTAL NO. AND

TOTAL VALUE INTAKA

WRITE TOTAL

QUANTITY AND

VALUE INTAKA

WRITE TOTAL

QUANTITY AND

VALUE INTAKA

THE TOTAL VALUE IN

TAKA

IF NONE, WRITE '0'.

Animal

Code

Number

Taka

Number

Taka

Number

Taka

Number

Taka

Animal product Code Unit

Meat (cattle,

Quantity Taka

Quantity

Taka

Quantity

Taka

Cattle

Goat

201

202

203

204

205

206

207

208

209

210

211

212

213

214

215

216

217

218

220

Kg

Kg

Kg

Lt

buffalo)

Poultry

Sheep

Buffalo

Chicken

Duck

Mutton, sheep

Milk

Eggs

No

No

Kg

Animal skins

Cow dung

Other

Pigeon

Otherdomestic Birds

Other(specify)

Total value

Total value

Page 21

7C1

HIES2016

SECTION 7: AGRICULTURE

PART C: NON-CROP ACTIVITIES (Fish farming, Fish capture and Other farm related)

9

Did you or anyone in your household engage in any fishing or fish farming in the

last 12 months?

13

Did you or anyone in your household engage in any farm forestry in the last 12

months?

1

2

Yes

No

1

2

Yes

No

>> Q13

>>PART D

FISH FARMING AND FISH CAPTURE

FARMFORESTRY

10

11

12

14

15

16

Source

How much did you

How much did your

How much did your

Forest Product

How many trees do you How much did your How much did your household

produce (catch) in the past household sell in the past householdconsume in the

12 months? 12 months? 12 months?

presently own? What is

their total value?

household sell in the consume in the 12 months?

last 12 months?

WRITE TOTAL

QUANTITY AND VALUE QUANTITY AND VALUE QUANTITY AND VALUE

INTAKA INTAKA INTAKA

WRITE TOTAL

WRITE TOTAL

WRITE THE TOTAL WRITE THE TOTAL VALUE

VALUE

Code

Kg

Taka

kg

Taka

kg

Taka

Tree

Code

No of trees

Taka

Taka

Taka

Fish farm

221

222

223

224

225

226

227

228

230

Bamboo

231

232

233

234

235

236

237

238

239

240

Fish Hatchery

Timbertrees

Fruit trees

Marine fishing

Canal/riverfishing

Swampland/marsh/fenfishing

Pond/sinkfishing

Fish fry, crab frog and Other, specify

Dry fish

Firewood trees

Honey

Sapling/seedlingsale

Flower sale

Flower /fruit seed sale

Other, specify

Total value

Total value

Page 22

7C2

HIES2016

SECTION 7: AGRICULTURE

PART D: EXPENSES ON AGRICULTURAL INPUTS

1

2

Did yourhousehold spend any money on the (item) in How much did yourhousehold spend on the

last 12 months?

(item) in the last 12 months?

Expenditure item and code

1

2

Yes

No

>> NEXT ITEM

Items of expenditure

Code

Code

Kg

Taka

Seed seedling (crop seedling)

Seed (forest seedling)

301

302

Fertilizer(chemical)

303

304

Fertilizer(composed/mixed)

Food of livestock/ draft animal

305

306

307

308

309

311

312

313

314

315

316

317

318

319

321

322

330

Tractor/ tiller/ power tiller (rental)

Irrigation expenses

Insecticides

Land revenue (agricultural land)

Rent (agricultural land)

Carrying cost of goods and transportation expenses (agricultural goods)

Salary/wages of laboreremployed in agriculture

Insurance expenses (agriculture related)

Interest of the Agriculture loan

Electricity and fuel cost

Bees culture expenses

Fish production expenses

Livestock rearing expenses (Treatment cost etc.)

Poultry rearing expenses (Treatment cost etc.)

Other(specify)

Total value

7D

Page 23

HIES2016

SECTION 7: AGRICULTURE

PART E: AGRICULTURAL ASSETS

1

2

3

4

Equipment&assets used in

agriculture

How many ..[NAME OF ASSET].. do you

presently own? What is their total value?

How many did yourhousehold buy in the last 12 How many did yourhousehold sell in the last 12

months? months?

How much did yourhousehold earn from rental of

this item in the last 12 months?

TOTAL NUMBER AND THE TOTAL VALUE IN TOTAL NUMBER AND THE TOTAL VALUE IN TAKA

TAKA

WRITE TOTAL VALUE IN TAKA

Farming asset

Code

Number

Taka

Number

Taka

Number

Taka

Taka

Tractor

401

402

403

404

405

406

407

408

409

411

412

413

414

415

416

417

418

419

420

Thresher

Power tiller

Powerpump

Hand pump

Plough and yoke

Deep tube-well

Shallowtube-well

Sprayer

Husking machine

Ginning machine

Country boat

Engine boat

Fishing net

Cage incubator

Brooder

Bees-box

Other(specify)

Total value

Page 24

7E

SECTION 8: OTHER ASSETS AND INC O ME

HIES2016

PART A: OTHER PROPERTY AND ASSETS

1 Is there any land or property which yourhousehold owns but doesn't operate?

(don't include property already reported in sections 5, 6 &7)

1

2

Yes

No

Acres

Decimals

>>Q3

AMOUNT

TAKA

2 How much would it cost to buy land or property owned by yourhousehold?

3 Did yourhousehold purchase any land or property during the last 12 months?

1

Yes

2

No

>>Q5

4 How much did yourhousehold spend on purchasing this land or property?

TAKA

TAKA

5 Did yourhousehold purchase any house or flat over the past 12 months?

1

2

Yes

No

>>Q7

6 How much did yourhousehold spend on purchasing that house or flat?

7 Does yourhousehold own any other assets (e.g. stocks, bonds, otherfinancial

assets, jewelry, etc. not reported earlier)

1

2

Yes

No

>>Q9

8 How much in total are these worth?

TAKA

TAKA

9 Did yourhousehold purchase any assets other than land over the past 12 months?

1

2

Yes

No

>> Q11

10 How much did yourhousehold spend on purchasing these assets?

11 Did yourhousehold sell any assets over the past 12 months?

1 Yes

2

No

>>Part B

12 How much did yourhousehold get from selling these assets?

TAKA

8A

Page 25

PART B: OTHER INCOME

HIES2016

SECTION 8: OTHER ASSETS AND INC O ME

1 Income from rent of land received during the past 12 months:

8 Remittances received from relatives during the past 12

TAKA

TAKA

TAKA

TAKA

months:

(CASH AND IN-KIND FROM WITHIN THE COUNTRY)

2 Income from rent of other property received during the past 12

9 Remittances received from relatives during the past 12

months:

months:

(CASH AND IN-KIND FROMOUTSIDE THE COUNTRY)

3 Social and insurance (life and non-life) income received

during the past 12 months:

10 where did you mainly invest/spend the received money?

Code

Life Insurance TAKA

Health Insurance TAKA

General Insurance TAKA

1 Construction

2 Business

3 Education

4 Marriage

5 Consumption

6 Treatment

7 Other(Specify)

4 Profit and dividend received as partner/share holder during

the past 12 months:

TAKA

11 Gratuity, separation payment, retirement benefit received

TAKA

TAKA

during the past 12 months:

(DO NOT INCLUDE INCOME ALREADY REPORTED IN SECTION 5)

5 Lottery/prize bond/ other similar income received in cash or in-

12 Interest received during the past 12 months:

TAKA

TAKA

kind during the past 12 months:

(FROMBANKS AND OTHER SOURCES)

6 Charity, gift, royalty, help, zakat, fitra or other such assistance,

etc. received during the past 12 months: IN CASH

13 Other cash or in-kind receipts during the past 12 months:

TAKA

(DO NOT INCLUDE RECEIPTS REPORTED

7 Charity, gift, royalty, help, zakat, fitra or other such assistance,

TAKA

etc. received during the past 12 months: IN-KIND

Page 26

8B

HIES 2016

SECTION 8: OTHER ASSETS AND INC O ME

PART C: MIGRATION AND REMITTA NCE

1 Has any member of yourhousehold migrated, either within the country or abroad, during the last 5 years?

1 Yes >>Q 3 2 No

2 Has this household received any remittances from anyone living outside the household, either within Bangladesh or abroad, in the last 2 years?

1 Yes

2 No

>>Part D

3

4

5

6

7

If in-

country, write

write zila country

8

9

10

11

12

13

14

15

How did

(name)

16

17

Name

Relation- When did (name) Where is

ship to

head of

the H.H.

If abroad, Age Sex

Level of

education

Literacy

Occupation How many What is

Write the code What is

of any goods/

things that

migrate?

(name)

working?

times

(name)

the total

amount of send

the total

value of

SEE

SECTION 4

FOR CODE

P

E

R

S

O

N

code.

code.

send money money that money to (name) has sent all the

during the (name) the house- to the goods/

hold? household in the things

last 2

years?

has sent

over the

last 2

last 2 years.

that

(name)

sent in

the last 2

years?

I

LIST UP TO 3 years?

D

IN ORDER OF

IMPORTANCE

1 In country

>>Q 9

1 Ma le

2 Female

2

Abroad

CODE

>>Q 8

CODE

MO NTH YEAR

CODE

CODE

CODE

TAKA

CODE

1

2

3

TAKA

91

92

93

94

95

96

97

98

99

CODES FOR Q4 - RELATIONSHIP TO HOUSEHOLD HEAD

CODES FOR Q8 - COUNTRY CODE

CODES FOR Q16 - ITEM

01 Head

08 Niece/Nephew

09 Father/Mother-in-law

10 Brother/Sister-in-law

11 Other relative

12 Servant

01 Saudi Arabia

02 Qatar

03 Kuwait

04 Oman

05 Malaysia

06 Singapore

07 Iraq

10 United Arab Emirates

1 Food

02 Husband/Wife

03 Son/Daughter

04 Spouse of Son/Daughter

05 Grandchild

06 Father/Mother

07 Brother/Sister

11 Canada

12 Australia

13 U.K.

14 USA

15 Korea (South)

16 J apan

19 Sweden

20 Federation of Russia

21 Italy

22 Other European Country

23 Brunai

24 Mauritius

2 Electronic Goods (TV, VCD,

etc.)

3 Mobile Phone

4 Computer

5 Vehicle

6 Clothing

13 Employee

14 Other(specify)

08 Iran

09 Libya

17 Turkey

18 Germany

25 South Africa

26 Other(specify)

7 Household Appliance

8 Other(specify)

CODES FOR Q11 - EDUCATIONCODES

00 No class passed/pre-schooling

10 SSC/equivalent

01 Class 1

02 Class 2

06 Class6

07 Class 7

11 HSC/equivalent

12 Vocational

16 Medical

17 Engineering

CODES FOR Q13 - FREQUENCY

CODES FOR Q15 - METHOD

1 Once

5 Mo re than five times

1 Western Union

5 Through Friend/Relatives

03 Class 3

08 JSC/equivalent

09 Class 9

13 Nursing

18 Post graduate/

equivalent

2 Twice

6 Never send

2 Money Gram

6 Through Travel Agency

04 Class 4

14 Technical Education

3 Three times

4 Four times

7 Other(Specify)

3 Postal Money Order 7 Agent/Broker

4 Through Bank 8 Other(specify)

05 PEC/equivalent

15 Graduate/equivalent 19 Other(specify)

Page 27

8C

HIES 2016

SECTION 8: OTHER ASSETS AND INC O ME

PART C: MIGRATION AND REMITTA NCE

3

18

P

E

R

S

Name

How did you spend the remittance received during last two years?

A

B

C

D

O

N

Current Expenditure

(in last year)

Investment

Expenditure (in Consumer durables

last 2 years) (in last 2 years)

Expenditure for

Savings (in last 2 years)

I

D

CODE Amount (Tk) CODE Amount (Tk) CODE

Amount (Tk)

CODE

Amount (Tk)

91

92

93

94

95

96

97

98

99

Codes for A of Q18 Codes for B of Q18

Codes for C of Q18

1 Vehicles

Codes for D of Q18

1 Purchasing shares/

bond/equity

2 Deposit in bank

3 Purchase of valuables

(gold, diamond etc)

4 Cash in hand

1 Food and clothing

2 Education

3 Health

1 Construction of residential/

non- residential structures

2 Major repair and maintenance

work of residential/non-

residential structures

2 Refrigerator

3 Air cooler

4 Television

5 Other equipment for

household

4 Other (specify)

3 Purchase of land

4 Purchase of residential/

commercial space

use (specify)

5 Other savings (specify)

5 Purchase of agricultural

machineries and equipment

6 Purchase of transport

machineries and equipment

(for commercial purposes)

7 Repayment for past borrowing

8 Major repair and maintenance

of machineries

and equipment

9 Other investment expenditures

(specify)

Page 28

8C2

HIES 2016

SECTION 8: OTHER ASSETS AND INC O ME

PART D: MICRO CREDIT

Respondent: Head of household

1

2

3

4

Have you or any member of your

Have you or any member of yourhousehold deposit money in the

Have you or any member of your

Have you or anyone in yourhousehold

borrowed money from a family member,

household open a bank account during the credit or microfinance institution (BRAC, Grammeen Bank, etc) in the household deposit money in any

last 12 months?

last 12 months?

informal depositor organization in the friend, micro finance institution, bank or

last 12 months?

other source in the last 12 months?

1 Yes

2 No

1 Yes

2 No

1 Yes

2 No

1 Yes

2 No

>>S ection 9

INTERVIEWER: Use a separate line for every line, put in the ID code of the person who borrowed the money.

5

6

7

8

9

10

11

12

13

14

What was

How much How long is Interest rate applied on loan (%) How often do you make payments Have you

Amount of What was the Would you like to If yes,

the source money was the

of this loan borrowed (in repayment

and what is the amount of each

payment?

completed

repayment of loan (in

your loan?

unpaid

purpose of

receiving

loan?

have borrowed

more money at

the same rate of

interest?

how

much?

L

O

A

N

or credit?

taka)?

period (in

months)?

taka)?

FILL IN ONLY MONTH OR ONLY

YEAR

1 Y es>>Q12

2 No

USE CODES

1 Yes

USE

CODES

2 No

>>Next person

NUMBER

OF

MO NT HS

MONTHLY RATE YEARLY RATE FREQUENCY CODE AMOUNT

(%) (%) TAKA

CODE

TAKA

CODE

TAKA

CODE

CODE

TAKA

20

21

22

23

24

25

26

27

28

CODES FOR Q9 -

FREQUENCY OF PAYMENT

CODES FOR Q5 - SOURCE OF LOANOR CREDIT

CODES FOR Q12 - PURPOSE OF LOAN

01 Private Commercial Bank

02 Public Commercial Bank

03 Krishi Bank/Rajshahi Krishi Bank

04 Co-operative Bank

09 BRAC

10 BRDB

17 Money Lender

18 Land Lord

19 Employer

20 Friends

1 Once

1 Education

2 Daily

2 Health

11 Other Govt. Department

12 ASA

13 Proshika

3 Weekly

4 Fortnightly

5 Monthly

3 Agriculture

4 Business

5 Housing

05 Co-operative association

21 Relatives

06 BSIC

07 Youth Development

14 Other NGO

15 Other Micro Finance Establishment

22 Grocery Store

23 Other(specify)

6 Yearly

7 Other(specify)

6 Food Purchase

7 Marriage

08 Grameen Bank

16 Input supplier

8 Other(specify)

Page 29

8D

HIES 2016

SECTION 9: CONS UMP TION

PART A: DAILY CONSUMPTION

A

Less than 10 years old

No. of boys

B 10 years and older

No. of men

Date (Day/ Month/

Year)

No. girls

No. of women

1. Purchase

2. Wage in-kind

3. Self Prod.

4. Gift

1. Purchase

2. Wage in-kind

3. Self-prod

4. Gift

Quantity

Value

Value

DAY 1

Quantity

DAY 1

Taka

Ps

Major Source

Taka

Ps

Major Source

1

Food grains

010

Dried fish

Eel fish

sea fish

Baila

054

055

056

057

gm

gm

gm

gm

gm

Rice - Fine

Rice - Medium

Rice - Coarse

Beaten rice

Pop rice

011

012

013

014

015

016

017

018

019

021

022

023

gm

gm

gm

gm

gm

gm

gm

gm

gm

gm

gm

gm

gm

Other(specify)

058

4

5

Eggs

060

Puffed rice

Wheat(Atta)

Flour

Hen egg

061

062

No

No

No

Duck egg

Other(specify)

063

Vermicelli/Suji

Bread/Bonroti

Biscuits

Meat

070

Beef

071

072

073

074

075

076

gm

gm

gm

gm

gm

gm

gm

Buffalo

Mutton

Sheep

Hen

Cake

Other(specify)

024

2

Pulses

030

Lentil (musur)

031

032

033

034

035

gm

gm

gm

gm

gm

gm

Duck

Chickling-Vetch(mug)

Green gram (boot)

Pea gram (kheshari)

Mashkalai

Other(specify)

077

6

Vegetables

080

Potato

Brinjal

081

082

083

084

085

086

087

088

089

091

092

093

094

095

096

097

gm

gm

gm

gm

gm

gm

gm

gm

gm

gm

gm

gm

gm

gm

gm

gm

Other(specify)

036

White gourd/ Pumpkin

Watergourd

3

Fish

040

Hilsa

041

042

043

044

045

046

047

048

049

051

052

053

gm

gm

gm

gm

gm

gm

gm

gm

gm

gm

gm

gm

Balsam apple

Rhui/Katla/ Mrigel/ Kali baush

Perbol(Patal)

Pangash

Boal/Air

Magur/Shing

koi

Snake gourd/ Ribbed gourd

Greenbanana/ Greenpapaya

Arum/Ol-kachu/Kachur-mukhi

Cauliflower/Cabbage

Bean/Lobey

Silvercarp/Grass carp/ Miror carp

Shoal/Gajar/Taki

Tomato

Puti/BigPuti/Telapia/Nilotica

Mala-kachi/Chala-chapila/Khalsha

Other small fishes (with tangra)

Shrimp

Radish

Ladies' finger

(All types of leafy veg.(Spinach/ Amaranta/ Basil)

Other(specify)

Page 30

9A1-1

HIES 2016

SECTION 9: CONS UMP TION

DAY 1

PART A: DAILY CONSUMPTION

1. Purchase

Value

1. Purchase

2. Wage in-kind

3. Self-prod

4. Gift

Quantity

2. Wage in-kind

3. Self-prod

4. Gift

Quantity

Value

DAY 1

Taka

Ps

Major Source

Taka

Ps

Major Source

7

Milk &Dairy

Liquid milk

100

101

102

11 Drinks

150

151

152

Softdrinks(peepsi/RC/Mojo/Coke, Sherbat, etc.

ml

ml

Powdermilk

gm

Ovaltine/ Horlicks

gm

Curd

103

104

gm

gm

Tea/ Coffee leaf

Liquid (Ros) of Sugarcane/ Date/Palm

153

154

gm

ml

Casein(ponir)/ Butter

Milk drinks

105

106

110

ml

Green coconut water

Other(specify)

155

156

160

ml

Other(specify)

Sweetmeat

8

9

12 Sugar&molasses

Rasogolla/ Chamcham/ Shandash

Jilapi/ Bundia/ Amriti

111

112

gm

gm

gm

gm

Sugar/ Misri

161

162

163

164

gm

gm

no

Molasses (Sugarcane/ Date/ Palm)

Khaja/ Logenze/ Toffee

Chocolate

Halua/ Batasha/ Kadma

Other(specify)

Oil &Fats

113

114

120

no

Ice-cream

165

166

170

no

Mustard oil

121

gm

Other(specify)

13 Miscellaneous Food

Soybeanoil

Palm oil

122

123

124

125

gm

gm

gm

gm

gm

Pickles

171

172

173

174

gm

gm

gm

gm

Dalda/ Vanashpati

Ghee

Jelly/ Jam

Amshatta

Sauce/Sirka

Other(specify)

126

10 Fruits

Ripe banana

130

Other(specify)

175

14 Dining out (Food outside)

180

131

132

133

134

135

136

137

138

139

141

142

143

144

145

146

147

148

gm

gm

gm

gm

gm

gm

gm

gm

gm

gm

gm

gm

gm

gm

gm

gm

gm

Mango

Meals (Rice/Biriani)

181

182

183

184

185

186

187

188

189

191

192

193

194

195

200

201

gm

gm

gm

gm

gm

gm

gm

gm

gm

no

Melon/Bangi

J ack fruit

Leeches

Fried chicken

Fish

Meat

Ripe papaya

Guava

Patties/Cake

Sandwich

Pineapple

Safeda

Burger

Hotdog

Palm

Pizza

Bedana

Samucha/Singara/Puri

Tea

Apple

cup

cup

ml

Orange

Coffee

Grape

Softdrinks/bottle water

Other(specify)

15 Tobacco &tobacco products

Cigarette

Black berry

Amra/Kamranga

Others (specify)

no

Tobacco leaf

Biries

Gul and Other(specify)

202

203

204

gm

no

gm

9A1-2

Page 31

HIES 2016

SECTION 9: CONS UMP TION

DAY 1-7

PART B: WEEKLY CONS UMP TION

Date (Day/ Month/ Year)

1

2

3

4

Purchase

Wage in-kind

Self-prod

Gift

Quantity

Value

TAKA

PAISA

Major

16 Spices

Dried chili

210

211

212

213

214

215

216

217

218

219

221

222

223

230

231

232

233

234

235

236

237

gm

Green chili

gm

gm

gm

gm

gm

gm

gm

gm

gm

gm

gm

Onion

Garlic

Turmeric

Salt

Ginger

Cummin

Coriander-seed

Aromatic-seed

Clove/ Black pepper/ Cassia-leaf

Other(specify)

17 Betel leaf &Chewgoods

Betel leaf

gm

gm

gm

gm

gm

no

Betel nut

Zorda/ tobacco leaf

Lime

Khoer

Rolled betel leaf

Other(specify)

gm

9B1

Page 44

HIES 2016

SECTION 9: CONS UMP TION

PART B: WEEKLY CONSUMPTION

Date (Day/ Month/ Year)

1

2

3

4

Purchase

Wage in-kind

Self-prod

Gift

Quantity

Value

DAY 8-14

TAKA

PAISA

Major

16 Spices

210

Dried chili

211

212

213

214

215

216

217

218

219

221

222

gm

gm

gm

gm

gm

gm

gm

gm

gm

gm

gm

gm

Green chili

Onion

Garlic

Turmeric

Salt

Ginger

Cummins

Coriander-seed

Aromatic-seed

Clove/ Black pepper/ Cassia-leaf

Other(specify)

223

17 Betel leaf &Chewgoods

230

Betel leaf

231

232

233

234

235

236

237

gm

gm

gm

gm

Betel nut

Zorda/ tobacco leaf

Lime

Khoer

gm

no

Rolled betel leaf

Other(specify)

gm

9B2

Page 59

HIES 2016

SECTION 9: CONSUMP TION

PART C: MONTHLY NON-FOOD EXPENDITURE

1

2

3

What was the value of goods

consumed that were bought in

cash/ credit or wages in-kind?

What was the value of goods

consumed that were produced

by the household or received?

SUMOF COLUMN 1 AND 2

Taka

Taka

Taka

18 FUEL AND LIGHTING

240

Firewood

241

Cowdung/cakes/bhushi/wood-powder

242

243

244

J ute stick

Kerosene

Agri by products fuel: paddy, hag, pressed sugarcane and dried com

plants, etc.

245

Gas (natural, bio-gas)/LPG

Electricity

246

247

248

249

Pit coal/char coal/wood coal

Otherfuels and light (e.g. matches and candles etc.)

19 COSMETICS AND OTHER EXPENSES

250

Snow/cream/ powder

251

Perfume etc.

252

253

254

Haircutting, styling, shaving, etc.

Hair oil, hair cream, combs, clips, etc.

Razor, razorblades, shaving cream and lotions, etc.

Lipstick, nailpolish, make-up box etc.

255

256

257

Beautifying items (hairribbon, churi, kajal, etc.)

20 HOUSEHOLD HYGIENE (WASHING AND CLEANING) EXPENSES

Bath soap, shampoo, conditioner, toothpaste, etc.

Washing soap, powder for cloths

260

261

262

263

264

265

Washing/laundryexpenses

Bleaching powder, soda etc.

Vim/dish cleaning supplies

Page 60

9C1

SECTION 9: CONS UMP TION

HIES 2016

PART C: MONTHLY NON-FOOD EXPENDITURE

1

2

3

What was the value of goods

consumed that were bought in

cash/ credit or wages in-kind?

What was the value of goods

consumed that were produced by the

household or received

SUMOF COLUMN 1 AND 2

Taka

Taka

Taka

Finis/ phenyl/ airfreshener/ other household cleaning supplies

Toiletpapers/Tissue papers

266

267

268

269

271

272

273

280

281

282

Sanitary pad/Sanitary napkin/diaper etc

Charge for household solid waste disposal

Expenses for pit emptying

Mosquito coil

Mosquito spray, Insect spray (Aerosol)

21 TRANSPORT/ TRAVEL AND OTHER MISC. CHARGES

Bus fare

Rickshaw/ van fare

Taxi/ tempoo/mishuk fare

Boat/ launch fare

283

284

Train fare

285

286

287

288

289

Other transport fare (specify)

Bicycle maintenance, tyres, tubes repairs etc.

Motor-cycle maintenance, repairs, etc.

Car maintenance, repairs, etc.

Boatmaintenance, repairs, etc.

291

292

293

294

Petrol

Diesel

Motor oil/CNG. etc

Othertransport, repair and maintenance.

Telephone bill/ charges/mobile/internetbill

295

296

Telegram, postal and courier service expenses, etc.

Salaries and wages of servants

297

298

299

Salaries and wages of drivers

Salaries and wages of others including guards, gardeners, housekeepers

etc.

301

Over Night at hotel, boarding house, mess for travel purposes

Othercontegencies expenses (specify)

302

303

Page 61

9C2

HIES 2016

SECTION 9: CONS UMP TION

PART D: A NNUA L NON-FOOD EXPENDITURE

1

2

1

2

Expenditure/consumption during the past 12 month

How many did you

What was the

Expenditure/ consumption during the past 12 month

How many did you

What was the value

buy/produce at

home/receive as gift?

value of these

goods?

buy/produce at

home/receive as gift?

of these goods?

No.

Taka

No.

Taka

22 READY-MADE GA RMENTS

310

23 CLOTHING MATERIAL AND TAILORING

340

For adult:

Lungi/dhuti

Mill-made cloth/ fabric

341

311

312

Shirts

Hand loom cloth/ fabric

Drill and other cloth for trousers

Woolen cloth

342

343

344

345

346

347

348

349

Pant

313

314

315

316

317

318

319

321

Saree

Blouse/ peticoat

Salwar kameez/ Orna

Punjabi/ Pajamas

Suit, overcoat, ashkan, etc.

Sweaters, J acket, pullovers, mufflers, etc.

Underwear etc.

Silk

Artificial silk etc.

Otherartificial-yarn made cloths

Tailoring expenses

Other clothing related expenses

Socks, handkerchiefs, scarves, caps,

neckties etc.

322

323

24 FOOTWEAR

350

Other(specify)

For children:

Full pant

Leather shoes

351

352

353

354

355

356

357

Leathersandal-shoes

Leathersandal

324

325

326

327

328

Half pant

Plastic shoes

Shirts

Plastic sandal-shoes

Other sandal

T-shirt

Frocks, dresses, babysuit etc.

Canvas shoes, sports shoes, etc.

Socks, handkerchiefs, scarves, caps,

neckties etc.

329

Wooden sandals

358

359

Sweaters, J acket, pullovers, mufflers, etc.

Other for children (specify)

331

332

Shoe brush, polish, cleaning supplies, etc.

Maintenance and repairexpenses of foot wear 361

Otherexpenses regarding footwear 362

Both

Towel, Gamcha

Chador, shawl, etc.

Other(specify)

333

334

335

Page 62

9D1

HIES 2016

SECTION 9: CONSUMP TION

PART D: ANNUAL NON-FOOD EXPENDITURE

1

1

Expenditure/ consumption during the past 12 months

What was the value of goods bought/

Expenditure/ consumption during the past 12 month

What was the value of goods

produced at home/ received as gift?

bought/ produced at home/ received

as gift?

Taka

Taka

25 HOUSEHOLD-USE TEXTILES, ETC.

370

371

372

373

26 HOUSING RELATEDEXPENSES

390

Quilt/blanket/Katha

Toshok

391

House rent (rented house without water & electricity services)

J ajim

Imputed rent (owner-occupied or other)

392

393

Waterexpenditure (boughtbottle or delivered by water

truck/water/sewagre services)

Foamsheet

374

Bedsheets

375

376

377

378

379

381

382

383

Home additions/ improvements

Painting

394

395

396

397

398

399

Bed cover

Pillows, cushions

Pillowcover, cushioncover

Table cover

Disaster-relatedmaintenance/ repair

Otherroutine maintenance/ repair

Municipal tax

Curtain

Otherrelatedservices/ expenses

Mosquito netting

Other(specify)

28 MEDICAL TREATMENT EXPENSES (FEMALE)

Doctor's fees

420

421

422

423

424

425

426

427

728

429

431

432

433

434

27 MEDICAL TREATMENT EXPENSES (MALE)

Doctor's fees

400

401

402

403

404

405

406

407

408

409

411

412

413

Otherpractitioner's fees (homeopathetc.)

Medicines

Otherpractitioner's fees (homeopathetc.)

Medicines

Ayurvedic/ Kabiraji

Ayurvedic/ Kabiraji

Medical Tests (X-ray, blood, urine etc.)

Hospitalization, clinic charges, etc.

Dentalrelatedexpenses

Spectacles

Medical Tests (X-ray, blood, urine etc.)

Hospitalization, clinic charges, etc.

Dentalrelatedexpenses

Spectacles

Hearing aids

Hearing aids

Crutches

Crutches

Maternityexpenses

Contraceptive (condom, vesactomyetc)

Health-relatedtravel/ incidentalexpenses

Contraceptive (pill, ligationetc)

Health-relatedtravel/ incidentalexpenses

Page 63

9D2

HIES 2016

SECTION 9: CONS UMP TION

PART D: A NNUA L NON-FOOD EXPENDITURE

1

1

Expenditure/ consumption during the past 12 months

What was the value of goods

bought/ produced at home/

received as gift?

Expenditure/ consumption during the past 12 month

What was the value of goods bought/

produced at home/ received as gift?

Taka

Taka

29 EDUCATIONAL EXPENSES (MALE)

Registration fees

440

441

442

443

444

445

446

447

448

30 EDUCATIONAL EXPENSES (FEMALE)

Registration fees

450

451

452

453

454

455

456

457

458

Examination fees

Examination fees

Annual fees

Annual fees

School fees

School fees

Personal Teaching expenses

Text book/ note books/ stationary

Hostel Expenses

Personal Teaching expenses

Text book/ note books/ stationary

Hostel Expenses

Other(specify)

Other(specify)

31 REMITTANCES, CEREMONIES, GIFTS, ETC. 460

32 RECREATION&LEISURE, ETC.

Books, newspaper, magazines, story books

Cinema

480

481

482

483

484

485

486

487

488

489

Remittances to others living separately

Zakat

461

462

463

464

465

466

467

468

469

Fitra

Theater

Donation/ Sadqa

Variety shows, concerts, etc.

Sporting expenses, club membership fees, etc.

Video cassette purchases and rental etc.

Audio cassette purchases etc.

Photography

Qurbani

Religious functions (milad etc.)

Expenditure on Hajj

Expenditure on Pilgrimage

Expenditure on marriage

TV/ video/ satelite license fees etc.

Otherrecreation, marriage day/birthday, tourisum &

leisure related expenses

Expenditure on births

471

491

Expenditure on deaths

Other(specific)

472

473

Page 64

9D3

HIES 2016

SECTION 9: CONS UMP TION

PART D: A NNUA L NON-FOOD EXPENDITURE

1

1

Expenditure/ consumption during the past 12 months

What was the value of goods

bought/ produced at home/

received as gift?

Expenditure/ consumption during the past 12 month

What was the value of goods bought/

produced at home/ received as gift?

Taka

Taka

Page 64

9D3

HIES 2016

SECTION 9: CONSUMP TION

PART D: ANNUAL NON-FOOD EXPENDITURE

1

1

Expenditure/ consumption during the past 12 months

What was the value of goods bought/

produced at home/ received as gift?

Expenditure/ consumption during the past 12 Month

What was the value of goods

bought/ produced at home/ received

as gift?

33 TAXES, INTEREST, FINES, ETC.

500

36 PERSONAL ARTICLES

530

Income tax

501

Gold J welry

531

Bank interest charge, Payment of banking charge

Fines

502

503

504

505

506

507

508

Silver J welry

532

533

534

535

536

537

Imitation J welry

Legalpractitionerfees

Otherlegalexpenses

Property registration fees

Vehicle registrationfees

Other(specify)

Purses/ money bags

Vanity bags

Umbrella, walking stick

Tie-pin, cigarette cases, lighteretc.

Wrist watch/ clocketc.

538

539

Other personal use items (belts, etc.)

34 COOKING EQUIPMENT

510

Glass/china/clayplates and dishes etc.

511

37 MISC. HOUSEHOLDDURABLE

540

Refrigerators

512

513

514

515

516

Radio

541

542

543

544

545

Stove (electric/ gas/ kerosene)

Pots/ pans

Two-in-one

Black & White Television

ColorTelevision

VDO game set

Other kitchen ware and utensils

spoons/ forks/ knives etc.

VCD/ VCR/dishantenna/cable membershipfees/cable

subcriptionetc

Micro oven/Pressure cooker

Other(specify)

517

518

546

547

Mobile phone

Washingmachine, iron, etc.

548

549

551

35 FURNITURE &RELATEDPARAPHERNALIA

520

Guitar/ orchestra/ harmoniumetc

Khat/ chokey

521

Typewriter, personalcomputer, laptop, tablet, etc.

Chair/ table/ dresing table /computertable etc.

Sofa

522

523

524

Lenten/ chimney lamp etc.

552

553

554

Electric fans, air-conditioners, coolers, etc.

Gas burner, rice cooker, blander, oven etc

Wooden/ steel almira/ wardrobe/ bookself

Trunks, suitcases, etc.

Furniture repair/ polish etc.

Otherfurniture (specify)

525

526

527

Sewing machine

555

556

Cameras/ camcorders/vedio camera etc

38 INSURANCE EXPENDITURE

Life insurance

560

561

562

563

Healthinsurance

Generalinsurance

Page 65

9D4

HIES 2016

SECTION 9: CONS UMP TION

PART E: INVENTORY OF CONSUMER DURABLE GOODS

1

2

3

4

Does you household own any of the following items?

How many ..[ITEM].. Does your If you were to sellthis/these ..[ITEM].. If this … [ITEM] … was purchased

household own?

today, how much money would you

receive?

in the last 12 months, how much

itdid you pay for it?

PUT A CROSS MARK (X) IN THE APPROPRIATE BOX FOR ALL ITEMS IF THE

ANSWER IS YES OR NO

NUMBER OF THE ITEMOWNED

Item

Yes

No

Code

Number

Taka

Taka

Radio

571

572

573

574

575

576

577

578

579

581

582

583

584

585

586

587

588

589

591

592

593

594

595

596

597

598

599

600

Two-in-one, Cassette player

Camera/ camcorder

Bicycle

Motorcycle/ scooter

Motor car etc.

Refrigeratororfreezer

Washing machine

Fans

Heaters

Television

VCR/ VCP/DVD

Dish antena/ decoder

Pressure lamps/ petromax

Sewing machine

Bedroom Furniture

Drawing room Furniture

Dining room Furniture

Carpet

Kitchen Items - Cutlery

Kitchen Items - Crockery

Mocrooven/Kitchen Items - Cooking

Tubewell(fordrinking wateronly)

Wristwatch/Wallclock

Mobile

Computer/TV Card

Boat/Others

Total

Page 66

9E

HIES 2016

SECTION 1: HOUS EHOLD INFORMATION ROSTER

1

LIST THE NAME OF EACH AND EVERY HOUSEHOLD

MEMB ERS

THEN GO TO SECTION 1 PART A AND ASK THE

QUESTIONS

SEX

AGE

01

02

03

04

05

06

07

08

09

10

11

12

13

14

15
