# Supplementary material for: The burden of chronic diseases and patients' preference for healthcare services among adult patients suffering from chronic diseases in Bangladesh
Source: Health Expect. 2022 Oct 20;25(6):3259–73. doi: 10.1111/hex.13634 (PMC9700186; doi:10.1111/hex.13634)
Supplement: Supplementary file 2 — Supporting information. [file HEX-25--s002.docx]

Appendix Table A2. Study variable constructions

| Variable | Core question | Response options and recording |
| --- | --- | --- |
| Outcome variable | | |
| Patients’ preferences for chronic disease healthcare utilisation | Why did you choose this provider? Response options:  Nearby, Acceptable cost, Availability of doctor, Availability of female doctor, Availability of equipment, Quality of treatment, Referred by other providers, Referred by relatives/friends, Reputation, Other (specify) | Responses were categorised as ‘the quality of healthcare services’, ‘availability of medical doctors (e.g., availability of male or female doctors in a health facility)’, ‘affordable healthcare services (i.e., acceptable costs)’, ‘short distance to health facility (i.e., nearby)’, and ‘others (e.g., referred by other providers, Referred by other provider, Referred by relatives/friends, reputation, others). |
| Covariates | | |
| Illness related factors | | |
| Chronic illness | *Have you suffered from any chronic illness/disability in the last 12 months or more?*  *What chronic illness/ disability are you suffering from? with response options: 1) chronic fever, 2) Injuries/disability, 3) Chronic heart disease, 4) Respiratory Diseases/ Asthma/Bronchitis, 5) Diarrhoea/dysentery, 6) Gastric/ulcer, 7) Blood pressure, 8) Arthritis/Rheumatism, 9) Skin problem, 10) Diabetes, 11) Cancer, 12) Kidney diseases, 13) Liver Diseases, 14) Mental Health, 15) Paralysis, 16) Ear/ENT problem, 17) Eye problem, or 18 other (specify).* | *The prevalence of chronic diseases was assessed based on this question. The most reported prevalence of chronic diseases or conditions such as chronic heart disease, respiratory diseases, chronic gastric or ulcers, high blood pressure, arthritis or rheumatism, diabetes, chronic fever, and other diseases. In addition, the principal diagnosis was defined based on patients diagnosed with first chronic illness. Chronic comorbid conditions (principal diagnosis plus one, two, or three or more comorbid conditions) were assessed in this study.* |
| Healthcare services related factors | | |
| Type of healthcare services | *Where you admitted to hospital and stayed overnight during the last 12 months?*  *What was the total cost of outpatient treatment during the past 30 days?*  *What was the total cost of inpatient treatment during the past 12 months?* | *Type of healthcare services was recoded as ‘inpatient care’(i.e., patients received inpatient healthcare services) or ‘outpatient care’. These responses categorised were also valued with patients ‘reported inpatient and outpatient healthcare expenses at the last 30 days.* |
| Type of health care facilities | *Which of the following were consulted for this illness/injury (in the order in which they were consulted)? 1) Govt. Health Worker, 2) Govt. Satellite Clinic/EPI Outreach Center, 3) Community Clinic, 4) Union Health & Family Welfare Centrer/Union Sub Center, 5) Upazila Health Complex, 6) Maternal & Child Welfare Center (MCWC), 7) Govt. District/Sadar/General Hospital, 8) Govt. Medical College/Specialized Hospital, 9) Other Govt. Specify, 10) NGO Health Worker/Satellite Clinic, 11) NGO Clinic/Hospitals, 12) Govt. Medical College/Specialized Hospital,*  *13) Private Clinic/Hospitals, 14) Private Medical College/Specialized Hospital, 15) Qualified Doctor's Chamber, 16) Non-qualified Doctor's Chamber, 17) Pharmacy/Dispensary, 18) Homeopath, 19) Ayurbed/Kabiraj/Hekim, 20) Other Traditional/Spiritual, 21) Family/Self Treatment, 22) Other (Specify)* | *These responses were coded as ‘Pharmacy/Dispensary’, ‘Doctor's chamber’, ‘Public facility’, ‘Private facility’, or ‘Others’.* |
| *Location of consulted health care provider* | *Was the consulted provider urban or rural?* | *Location of consulted health care provider was recoded as ‘urban’ or ‘rural’.* |
| *Waiting time for receiving healthcare services, average days (sd)* | *How many days after symptoms began did you first consult this health provider? No. of days* | *We calculated average days after symptoms began to first consult with this provider?* |
| *Healthcare expenses* | | |
| *Average out-of-pocket payment, BDT* | *What was the total cost of outpatient treatment during the past 30 days?*  *Consultation fees (visit), cost of medicines, Routine medicines for chronic illness, Routine medical check up, cost of test/investigation, transport cost, total outpatient cost.*  *What was the total cost of inpatient treatment during the past 12 months?, including operational cost, Consultation/Doctor fees, Cost of investigations, Transport cost, Informal Tips, Bed/cabin charges, Cost of medicines, Other formal charges* | *An individual’s total OOP healthcare expenditure was derived by summing up direct medical costs and direct non-medical costs.* |
| Socio-demographic factors | | |
| Age | Write age in full years | Age was categorised into five groups: <18 years, 18-35 years, 36-45 years, 46-64 years, or 65 or more. |
| Sex | Sex: 1. Male or 2. Female | 1 = male or 2 = female. |
| Educational status | What was the highest class that you completed? | The participant’s educational status was recoded as ‘No education’, ‘Up to primary’, ‘Secondary education’ or ‘Higher’. |
| Marital status | Marital status: 1) Currently married,  2) Never Married, 3) Widowed, 4) Divorced, 5) Separated | Marital status was recoded as currently married, never married, or others (widowed/divorced/separated) |
| Religion status | Religion: 1) Islam, 2) Hinduism, 3)Buddhism  4) Christianity, 5) other (specify) | Religious status was regrouped as ‘Islam’, ‘Hinduism’ or ‘others’. |
| Employment status | Earner: Yes or No | 1= ‘Yes’ or 2 = ‘No’. |
