# Supplementary material for: The burden of chronic diseases and patients' preference for healthcare services among adult patients suffering from chronic diseases in Bangladesh
Source: Health Expect. 2022 Oct 20;25(6):3259–73. doi: 10.1111/hex.13634 (PMC9700186; doi:10.1111/hex.13634)
Supplement: Supplementary file 3 — Supporting information. [file HEX-25--s003.docx]

Supplementary document 1

We developed this document based on the Household Income and Expenditure Survey (HIES) 2016-17 and the Bangladesh Bureau of Statistics (BBS)’s data catalog application.

Source 1: <http://data.bbs.gov.bd/index.php/catalog/182>

Source 2: <http://www.bbs.gov.bd/site/page/648dd9f5-067b-4bcc-ba38-45bfb9b12394/Income,-Expenditure-&-Poverty>

**Data source of this study:**

We analysed secondary data from the nationally representative Household Income and Expenditure Survey (HIES) 2016-17 conducted by Bangladesh Bureau of Statistics (BBS). This survey was the sixteenth round of the HIES and was carried out from April 2016 to March 2017.

**Significance of HIES survey:**

HIES is one of the core activities of the Bangladesh Bureau of Statistics (BBS), it contains a wide range of socio-economic information at the household level that has strong bearing in the decision making process for the government. It is the standalone survey in Bangladesh to provide reliable and credible estimate of poverty and its correlates. It is widely used across the world, particularly in the low income developing countries, for assessing poverty level and the living standard of the people at large. Considering its importance, Government of Bangladesh, Particularly Bangladesh Bureau of Statistics (BBS) and Statistics and Informatics Division (SID) and the international agencies have been striving for the improvement of survey methodology and enhancement of HIES technical standard.

This survey provides valuable data on household income, expenditure, consumption, savings, housing condition, household's access to water supply and electricity, education, employment, health and sanitation, social safety nets, remittance, micro-credit, crisis coping, disability etc. The survey data can also be used for compilation of national accounts of the household sectors, analysis of poverty situation and other information on household related characteristics. It also provides the weights for computation of Current Price Index (CPI). It becomes the main data source for preparation of the Poverty Reduction Strategy (PRS) and Five Year Plan (FYP). It is also used for monitoring the progress of poverty reduction and the Sustainable Development Goals (SDGs) indicators.

**Objectives of the HIES 2016-17 survey:**

The main objectives of HIES 2016-17 (directly copied from the published report) are to:

- Obtain detailed data on household income, expenditure and consumption
- Determine poverty profile with urban and rural breakdown
- Provide reliable annual poverty estimates at 64 districts of the country along with national quarterly estimates
- Provide information about standard of living and nutritional status of population
- Provide data to determine the weights of consumer price indices
- Provide household level consumption data used in compiling national accounts estimate
- Provide detailed information on health status and educational level of the population
- Determine poverty estimates by administrative divisions and detailed socio-economic characteristics of the population and households
- Provide benchmark data for formulation of appropriate policy for poverty reduction, improvement in standard of living and nutritional status of the population
- Provide relevant data for monitoring of the Progress of 7 FYP and SDGs
- Provide data on nature, volume and distribution of resources under different Social Safety Nets programme
- Collect data related to calculation of demand function and elasticity
- Generate data for formulating appropriate fiscal policies
- Provide data on migration and remittances
- Collect detailed data on credit and repayment situation and practices
- Collect data on crises at household level, its impact and strategy for management

**Survey design:**

The HIES 2016 deviates from the sampling design used in the previous round of HIES 2010 in several ways. The objectives of HIES 2016 have changed significantly from HIES 2010. In HIES 2010, sample was designed to provide reliable annual estimates at division level with urban & rural break down. But in HIES 2016, the sample was designed to achieve three objectives:

1. reliable annual estimates at 64 district level
2. reliable quarterly estimates at the national level and
3. reliable annual estimates at the division level for urban and rural areas.

To achieve this multiple objectives, BBS needed to change the sample design of HIES 2016 significantly from HIES 2010. The first significant change was to increase the sample size to almost four times compare to HIES 2010. This substantial increase in the sample size also forced to use a new sampling frame instead of the previous Integrated Multi-Purpose Sample (IMPS). The IMPS is a master sample updated after each Census of Population and Housing. This IMPS was used as sampling frame for the selection of Primary Sampling Units (PSUs) for HIES 2010 and also for other surveys in BBS.

**Sampling frame:**

The frame used in the selection of Primary Sample Units (PS Us) for HIES 2016 was based on the Census of Population and Housing 2011. PSUs for HIES 2016 are the Enumeration Areas (EAs) used for the Census of Population and Housing in Bangladesh. Each EA is a cluster of 110 households on an average. The sampling frame for the selection of PSUs consists in the list of all EAs covering people residing in dwelling households (non-institutional households) in Bangladesh.

**Stratification:**

In the sample design of HIES 2016, two different levels of Stratification were followed:

i) As of HIES 2016, Bangladesh had eight administrative divisions. These were Barishal, Chattogram, Dhaka, Khulna, Mymensingh, Rajshahi, Rangpur and Sylhet. First, these 8 divisions of the country were stratified by 3 basic localities viz. Rural, Urban and City Corporation. Thus, there should have been 8×3=24 strata. But as the sampling frame (Population Census 2011) does not contain Rangpur city corporation and other two city corporations viz. Barishal & Sylhet are not much different from urban characteristics of these two city corporations, BBS included only the four main city corporations (Dhaka, Chattogram, Khulna and Rajshahi) in the city corporation locality. This brought the number of main strata to 20 (8 rural divisions + 8 urban divisions+ 4 main city corporations).

ii) Secondly, as the PSUs of HIES 2016 will be allocated at district (zila) level, the sample was implicitly sub-stratified at the district level. Since there are a total of 64 districts in Bangladesh, the sample design includes a total of 132 sub-strata: (64 rural, 64 urban and 4 city corporations).

**Sample size:**

Sample size was calculated using the prevalence rate of the main indicator (poverty rate) or the coefficient of variation of per capita consumption or household consumption, which are the core indicators of the HIES 2016-2017. Each one was treated as a target variable for determining the sample size. The required sample size was calculated for each district and explained elsewhere (BBS, 2017), using the following formula

$n=\left( \frac{Z_{\frac{\alpha}{2}}\times{CV}_{SRS\left( \bar{y} \right)}}{r\left( \bar{Y} \right)} \right)^{2}\times DEFF$ (1)

Where, n was the required sample for allocation to each district in order to achieve a certain level in the accuracy statistic ($r\left( \bar{Y} \right)$= 10% relative standard error desired for the mean total household expenditure estimated at the district level associated with the targeted variable $\left( \bar{y} \right);$ ${CV}_{SRS\left( \bar{y} \right)}$ was the coefficient of variation of the targeted variable (i.e., total household expenditure estimated at the national level) estimated under the assumption of simple random sampling; DEFF was the design effect of the target variable (i.e., the average design effect of the target variable across all districts); and $Z_{\frac{\alpha}{2}}$ ( = 1.96) was the critical value of a standard normal distribution with a α% (5%) significance level. Substituting all values in equation (1), the required sample was 715 households for each district, nonetheless, 720 households were allocated to each district for practical consideration and to facilitate field work and survey implementation management. A stratified, two-stage cluster sampling technique was used in this survey. At the first stage, a total of 36 PSUs were drawn from each district applying probability proportional to size (PPS) systematic sampling technique, using number of households in each PSU as the measure of size. The 36 PSUs were randomly selected from rural, urban and city corporation sub-stratum. The total number of PSUs included in the analysis were 2,304 (64 districts × 36 PSU per district). At the second stage, 20 households were selected per PSU. Using this sampling technique, 46,076 households (2,304 PUSs × 20 households per PSU) were included in study analysis of HIES 2016-2017 data. Among the selected households, a total of 186,076 individuals were included. Data collection was performed between early April 2016 and late March 2017. The survey objectives, sampling technique, survey design, survey instruments, measuring system, and quality control measures are described elsewhere (BBS, 2017).

Sample Allocation:

As one of our goals here is to estimate and compare Zila level means, equal allocation of PSUs to Zila may be a better choice. That is 36 PSUs will be assigned to each zila. Secondly these 36 PSUs will be allocated across rural, urban and city corporation sub-strata using modified Neyman's allocation technique which not only takes into account both the size and variability (standard deviation) of interest but also uses square root of design effect (deft) to modify the standard deviation. Although, the sample design is not supposed to provide Zila level estimates by rural, urban or city corporation, the Neyman's allocation taking into account the variability of the locality (rural/urban/city corporation) will greatly improve the precision of estimates at Zila as well as aggregate (National or Divisional) level.

Sample selection:

The HIES 2016 followed a stratified two stage cluster sampling design. At the first stage, a total of 36 PSUs (EAs) was drawn from each Zila (Domain) applying PPS systematic sampling technique, number of households in each PSU being the measure of size. These 36 PSUs were selected independently from rural, urban and city corporation sub-stratum. Therefore, in total, there will be 64x36=2,304 sample PSUs for the survey. Enumeration Area, a cluster of around 110 households of population census 2011, was treated as PSU for this sample design. The sampling frame for this purpose was developed from the population census 2011 data. A file containing all the EAs of the population census 2011 was created. This file contains all the unique geographic codes from division down to EA and also locality code (rural, urban and city corporation). In order to select the sample PSUs independently by stratum and Zila, the sampling frame was properly sorted by stratum and geo-codes. Then, at the first stage, the required number of PSUs (as shown in table-1, final HIES 2016-17 report) was selected using probability proportional to size (PPS) systematic sampling, size measure being the number of households in each PSU. After selection of the PSUs, a complete household listing in these selected PSUs was done in the field. Subsequently, this was computerized and used to draw the 20 households along with 5 reserved households from each of the selected PSUs at the second stage. Thus, total sample size for the survey stands at 2304x20=46,080 households.

**Training and Field Operation**

Before starting actual survey operation, detailed training was given to the enumerators and the supervisors. There were 128 enumeration teams for the survey. Each enumeration team comprised of 1 supervising officer, 2 interviewers cum data entry operators and 2 female facilitators. This team of five members was assigned to 1 PSU to work for a continuous period of 20 days: 14 days for collection of data and 6 days for data entry tasks and probable revisits and for the movements between PSUs.

For collection of information on food consumption, the households were divided into two groups each consisting of 10 households. Each enumerator, with the help of the female facilitator, continuously collected information on food consumption of the households for 14 days without break. After completion of data collection and data entry tasks for 3 terms, all the enumerators and the supervising officers were again trained in the headquarters, especially on those matters where some deficiencies or discrepancies were initially observed. This last training greatly enhanced the quality of data collection and data entry in the subsequent period.

**Supervision and Quality Control**

Strong supervision and quality control measures were adopted in HIES 2016. As mentioned earlier, there were 128 teams, each team comprising 2 enumerators cum data entry operators and 2 female facilitators. In order to ensure smooth collection of data and their quality, 64 supervising officers were appointed to supervise the work of 128 teams. The Deputy Directors of District Statistical Offices and officers form the HQs were engaged as supervising officers. In addition, 2 supervising officers were kept as reserve for meeting any emergency and 4 enumerators were also kept as reserve for the same purpose. Thus, the number of enumerators and supervising officers were 260 and 66 respectively. There were also senior officials from HQs who frequently visited the sample areas randomly to ensure the quality of survey data. The supervising officers were required to examine all the questionnaires completed by the field staff and also verify that each interview had been carried out in time and the questionnaires were completed correctly. They also ensured that the seasonal variations in income and expenditure pattern have been reflected in the collected data sets. In cases where further corrections were needed, the respective enumerators were instructed to do the same. The enumerators and the female facilitators used to inform the supervising officers of any problem they faced during the period and the supervising officers, in tum, helped the enumerators in solving their problems. The enumerators, soon after completion of data collection and data entry, sent the soft copy of the data sets to the headquarters through Dropbox and simultaneously sent the filled-in questionnaires either through special messengers or through courier service to the headquarters. These data sets were promptly verified in the headquarters. In case any error or inconsistency was found, it was immediately communicated to the concerned enumerator and the supervising officers. These control and supervising measures as mentioned above enhanced the quality of data and the data entry system to a great extent.

**Survey Instruments covering modules of the HIES 2016-17 survey:**

In the survey, participants were asked several questions under nine modules: 1) household information, 2) education, 3) health illnesses and injuries, 4) economic activities and wage employment, 5) non-agricultural enterprises, 6) housing, 7) agriculture, 8) other assets and income and 9) consumption. Please see the order of modules, which was directly copied from the original HIES 2016-17 questionnaire (See Appendix Table A2).

| **#** | **Order of modules** | **Page No** (HIES 2016-17 original questionnaire, Appendix Table A2) |
| --- | --- | --- |
| 1 | **Household Information Roster**  Part A: Household Information (with disability)  Part B: Employment Information  Part C: Social Safety Nets Programme | 1  2  3-4 |
| 2 | **Education**  Part A: Literacy and Educational Attainment  Part B: Current Enrollment | 5  6-7 |
| 3 | **Health**  Part A: Illness and Injuries | 8-11 |
| 4 | **Economic Activities and Wage Employment**  Part A: Activities  Part B: Wage Employment | 12  13 |
| 5 | Non-Agricultural Enterprises | 14-15 |
| 6 | **Housing**  Part A: Housing information  Part B: Shocks and Coping | 16  17 |
| 7 | **Agriculture**  Part A: Landholding  Part B: Crop Production  Part C: Non-Crop Activities   - Livestock and Poultry - Livestock Products - Fish Farming and Fish Capture - Farm Forestry   Part D: Expenses and Agricultural Inputs  Part E: Agricultural Assets | 18  19-20  21  21  22  22  23  24 |
| 8 | **Other Assets and Income**  Part A: Other Property and Assets  Part B: Other Income  Part C: Migration and Remittances  Part D: Micro Credit | 25  26  27-28  29 |
| 9 | **Consumption**  Part A: Daily Consumption   - Day 1 - Day 2 - Day 3 - Day 4 - Day 5 - Day 6 - Day 7   Part B: Weekly Consumption (Day 1-7)  Part B.A:   - Day 8 - Day 9 - Day 10 - Day 11 - Day 12 - Day 13 - Day 14   Part B.B: Weekly Consumption (Day 8-14)  Part B.C: Monthy Non-food expenditure  Part B.D: Annual Non-food expenditure  Part B.E: Inventory of Consumer Durable Goods | 30-31  32-33  34-35  36-37  38-39  40-41  42-43  44  45-46  47-48  49-50  51-52  53-54  55-56  57-58  59  60-61  62-65  66 |

**Data Entry, Processing and Validation**

The data collection, entry and data transferring process for the HIES 2016 was developed using Paper and Pencil Interviewing (PAPI) combined with Computer Assisted Field Entry (CAFE). With this method, the interviewers regularly collected all the information during the interview using PAPI and entered the data into Laptop Computers at the end of the day. If they found any inconsistencies in the data, they went back to the relevant households of the PSU and made required changes or corrections to remove the discrepancies while they were still in that locality. Once they had completed and checked the information, they also ensured that the data entered through data entry programme were accurate and consistent. Thus, the data were substantially cleaned and validated right at the field level.

The data entry programme was developed in CSPro and contained with a cloud based data transferring system, which allowed enumerators to transfer data from the field almost in real time using mobile internet connection. After the data was transferred to BBS headquarters, this was compiled and exported to a readable version by standard statistical software using a built-in routine in the data entry programme.

After the data entry was completed in the field, the filled-in questionnaires were also sent to the BBS headquarters. The transferred data were then promptly examined and verified with the questionnaires if necessary to ensure that the errors and inconsistencies that were required to be removed by the enumerators were done properly. The data sets then re-examined by programmers and senior officials. It may be mentioned that the software for the data entry task was developed in such a manner as to detect most of the errors, omissions or inconsistencies right at the data entry level. However, some more editing, specially inter record consistencies, were required to be done by the senior officials at BBS headquarters.

From the data sets thus produced, dta files were created through specially designed software. Finally, tables were generated from the cleaned data sets using data analysis software like STATA, FoxPro and SPSS.

**References:**

BBS, 2017. Preliminary Report on Bangladesh Household Income and Expenditure Survey 2016-2017. Bangladesh Bureau of Statistics, Statistics Division, Ministry of Planning. Government of the People’s Republic of Bangladesh.
